# Synthesis of the Hexahydropyrrolo-[3,2-c]-quinoline Core Structure and Strategies for Further Elaboration to Martinelline, Martinellic Acid, Incargranine B, and Seneciobipyrrolidine

**DOI:** 10.3390/molecules26020341

**Published:** 2021-01-11

**Authors:** Marianne B. Haarr, Magne O. Sydnes

**Affiliations:** Department of Chemistry, Bioscience and Environmental Engineering, Faculty of Science and Technology, University of Stavanger, NO-4036 Stavanger, Norway; marianne.b.haarr@uis.no

**Keywords:** natural product synthesis, hexahydropyrrolo-[3,2-c]-quinoline, scaffold, martinelline, martinellic acid, incargranine B, seneciobipyrrolidine

## Abstract

Natural products are rich sources of interesting scaffolds possessing a plethora of biological activity. With the isolation of the martinella alkaloids in 1995, namely martinelline and martinellic acid, the pyrrolo[3,2-c]quinoline scaffold was discovered. Since then, this scaffold has been found in two additional natural products, viz. incargranine B and seneciobipyrrolidine. These natural products have attracted attention from synthetic chemists both due to the interesting scaffold they contain, but also due to the biological activity they possess. This review highlights the synthetic efforts made for the preparation of these alkaloids and formation of analogues with interesting biological activity.

## 1. Introduction

Natural products have been and continue to be an immense source of inspiration for organic chemists looking for challenging synthetic targets to test new synthetic strategies and methodologies [1,2,3,4,5,6,7,8,9]. In addition, natural products are good sources for discovery of novel scaffolds, which is important inspiration for new structural motifs in medicinal chemistry and eventually for drug discovery [10,11,12]. As of today, approximately 50% of all drugs approved by the US Food and Drug Administration (FDA) are based on natural products or derived from a natural product scaffold [13,14,15,16]. Many natural products are rich in stereocenters and possess a high degree of unsaturated carbon bonds. These properties are highly desirable in the development of pharmaceutically active compounds, since the clinical success of drug candidates directly correlates to their three-dimensional structure [17,18].

**Figure 1:**
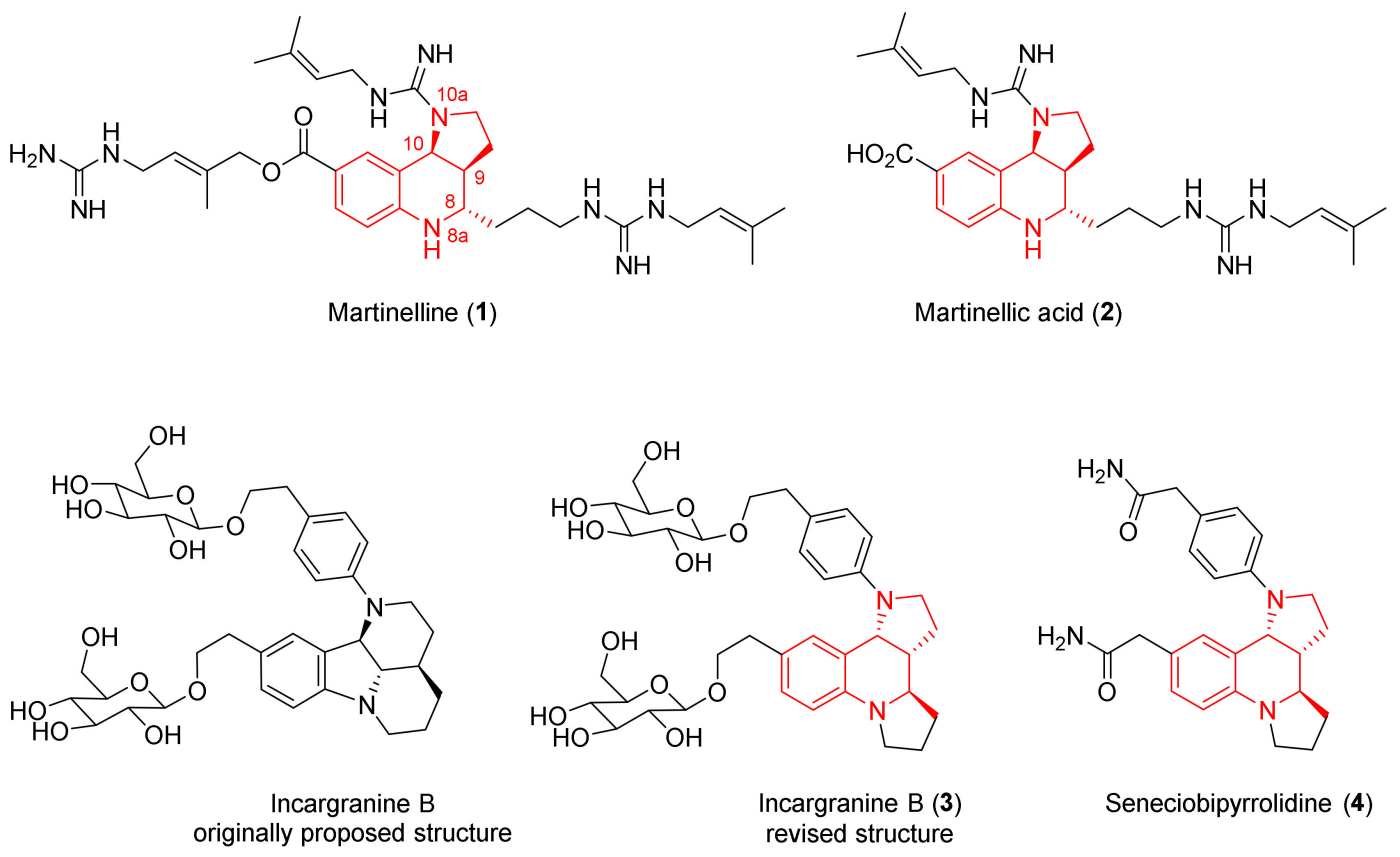
The structure of martinelline (**1**), martinellic acid (**2**), incargranine B (**3**) (originally proposed structure and revised structure), and seneciobipyrrolidine (**4**). The pyrrolo[3,2-c] scaffold is highlighted in red with atom numbering.

One such scaffold comprised of three stereocenters is the hexahydropyrrolo[3,2-c]quinoline scaffold, which was first discovered in the martinella alkaloids in 1995 (Figure 1) by Witherup and co-workers at the Merck laboratories [19]. They isolated martinelline (**1**) and martinellic acid (**2**) from the roots of the tropical plants *Martinella iquitosensis* and *Martinella obovata*. In 2016, martinelline (**1**) was also isolated from the leaves of *Emilia coccinea* (Sims) G Dons originating from Southern Nigeria [20]. The same year, martinellic acid (**2**) was identified in the Australian cane toad skin *Bufo marinus* [21], as well as in the flowering plant *Elephantopus scaber* [22]. The biological properties displayed by the martinella alkaloids included high binding affinity to bradykinin-, α*_1_*-adrenergic-, and muscarinic receptors, as well as antimicrobial activity [19]. In fact, the juice from the martinella and emilia plants have been used by South American natives and Nigerians, respectively, to treat eye infections [23,24]. The emilia leaves have also traditionally been used in Southern Nigeria for birth control [20].

Since the discovery of the martinella alkaloids, the partially reduced pyrrolo[3,2-c]quinoline scaffold has been found in two additional natural products, namely incargranine B (**3**) and seneciobipyrrolidine (**4**) (Figure 1). Incargranine B (**3**) was isolated from *Incarvillea mairei* var. *grandiflora* by the Zhang group in 2010. They incorrectly identified the alkaloid as comprising an indolo-[1,7]-naphthyridine core structure [25]. The Lawrence group suggested that incargranine B (**3**) contained a dipyrroloquinoline scaffold after structural revision of the alkaloid [26]. A second dipyrroloquinoline alkaloid was isolated in 2013 from *Senecio scandens* and termed seneciobipyrrolidine (**4**) [27]. Despite the fact that *Senecio scandens* is a common Chinese herbal medicine used for treatment of a variety of ailments [28], neither of the dipyrroloquinolines have been assessed for biological activity.

All four natural products (**1**–**4**) have been isolated with an *exo*-stereochemistry, i.e., a trans-relationship between protons H-8 and H-9 and a cis-relationship between protons H-9 and H-10 (see martinelline (**1**) in Figure 1). Witherup and co-workers reported optical rotation values of α = +9.4 (*c* = 0.02, MeOH) and α = −8.5 (*c* = 0.01, MeOH) for martinelline (**1**) and martinellic acid (**2**), respectively. From synthetic preparation of the enantiopure alkaloids accompanied by reports of their optical rotation values (Table 1), one may conclude that the isolated compounds consisted of epimeric mixtures [29,30,31,32]. After synthetic preparation of incargranine B ((±)**3**), the Lawrence group also concluded that the aglycon of the isolated alkaloid **3** was a mixture of epimers [26]. No values for optical rotation have been reported for synthetically prepared seneciobipyrrolidine (**4**). However, the α-value for the isolated seneciobipyrrolidine (**4**) reported by the Tan group (α = −72.9 (*c* = 0.10, MeOH)) may suggest that this alkaloid was of higher enantiopurity than the three other isolated natural products (**1**–**3**).

This review will highlight the synthetic efforts made for the preparation of these alkaloids, with particular focus on the assembly of the partially reduced pyrroloquinoline core structure, and formation of analogues with interesting biological activity. The review does not include synthetic approaches towards the structurally related hexahydroindolo-[3,2-c]-quinoline or the hexahydropyrrolo-[3,4-c]-pyrrole structures. Synthetic routes towards the martinella alkaloids have been reviewed in 2004 by Nyerges and in 2008 by Lovely and Bararinarayana [33,34]. Since 2008, new approaches towards the alkaloids have emerged, and medicinal applications of this core structure have been developed. Apart from specific prerequisite information, this review will not cover literature prior to the review in 2008.

## 2. Total and Formal Synthesis of Martinellic Acid (2)

**Scheme 1:**
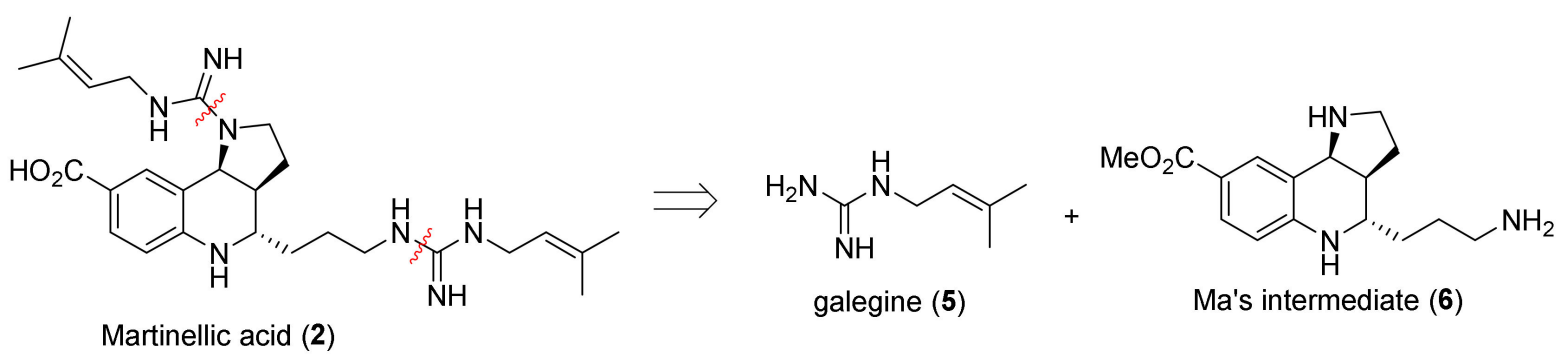
Retrosynthesis of the final assembly of Ma’s intermediate (**6**) and the galegine (**5**) side chains to form martinellic. acid (**2**).

**Scheme 2:**
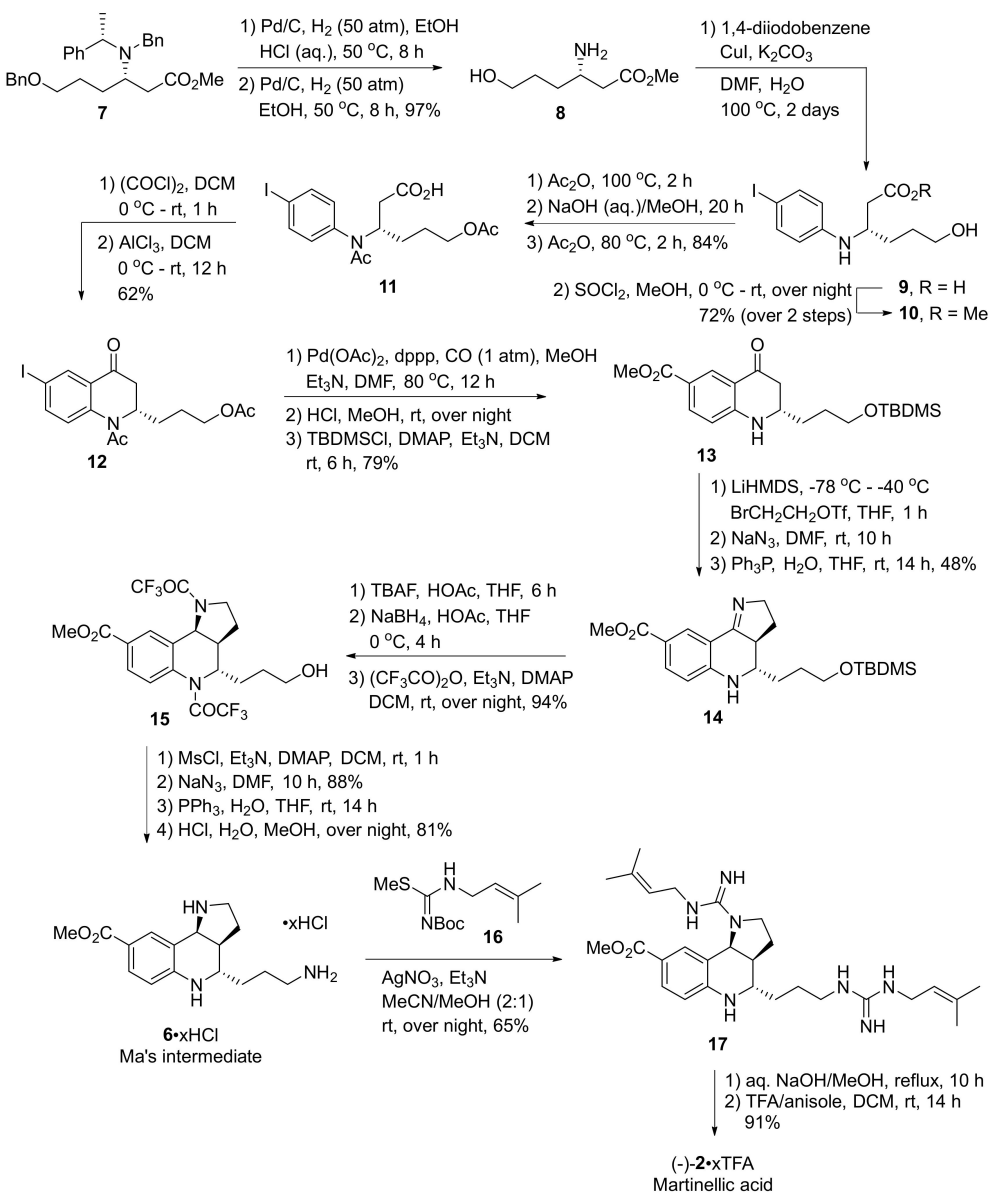
Ma and co-workers’ synthesis of Ma’s intermediate and the endgame to martinellic acid ((−)-**2**).

Synthesis of martinellic acid (**2**) has consistently been divided into two parts, namely assembly of the tricyclic scaffold and attachment of the galegine (**5**) side chains (Scheme 1). These guanidine-containing side chains were first isolated from *Verbesina encelioiodes* (Asteraceae) [35], and later identified as a toxic and antidiabetic component from *Galega officinalis* (Goat’s Rue) [36]. Guanylation of the martinella scaffold has been conducted under different reaction conditions [30,37,38,39], one of which is presented below in Ma and coworkers’ asymmetric synthesis of (−)-martinellic acid ((−)-**2**) from 2001 (Scheme 2) [29,37]. The precursor for the guanylation reaction in Ma and coworkers’ protocol, namely triamine **6**, has since 2001 been referred to as Ma’s intermediate (**6**) and has been a key target compound in a handful of total and formal total syntheses of the martinella alkaloids [30,32,38,39,40,41,42,43,44,45,46,47]. We have therefore included a description of Ma and coworkers’ asymmetric synthesis of (−)-martinellic acid ((−)-**2**) in this review.

In their 2001 publication, Ma and co-workers reported the first total synthesis of martinellic acid (**2**) [37]. The report was followed up in a full account in 2003 (Scheme 2) [29]. The synthesis was initiated from chiral β-amino ester **7**, which was obtained from 1,4-butandiol using a chiral auxiliary, following a procedure developed by Davies and co-workers [48]. Copper catalyzed coupling of 1,4-diiodobenzene with β-amino ester **8** provided acid **9**. In the following *N*-, *O*-acylation step, predominance of *O*-acylation over *N*-acylation of amino acid **9** was observed. It was thus found that the acid first had to be converted to the corresponding methyl ester **10** before treatment with acetic anhydride at elevated temperature facilitated acylation of both the amino- and hydroxy functionalities. The resulting *N*-, *O*-acylated methyl ester was then hydrolyzed to the corresponding acid followed by a re-acylation of the free hydroxyl group. This provided acid **11** in 84% yield from methyl ester **10**. Formation of the acyl chloride upon treatment with oxalyl chloride followed by an aluminum chloride (AlCl_3_) mediated intramolecular acylation provided ketone **12** in 62% yield.

Palladium-catalyzed carbonylation of the iodide moiety within compound **12** followed by deacetylation and silyl protection of the hydroxyl group provided ketone **13**. Alkylation of ketone **13** was conducted by enolization with lithium bis(trimethylsilyl)amide (LiHMDS) followed by addition of 2-bromoethyl triflate as the alkylating agent. Ma and co-workers found that the resulting ethyl bromide product was unstable and therefore converted the bromide directly to the corresponding azide with sodium azide. Reduction of the azide with triphenyl phosphine and water provided imine **14** in 48% yield from ketone **13**. Reduction of imine 14 was found to work selectively with sodium borohydride only after deprotection of the *O*-silyl group. Protection of the resulting diamine with trifluoroacetic anhydride provided compound **15** in 94% yield from imine **14**. A reaction sequence including mesylation, azidation, azide reduction, and *N*-deprotection gave triamine **6** (Ma’s intermediate) as the HCl salt.

To further convert the triamine 6 to martinellic acid, the primary amine and the hindered secondary amine functionality in the pyrrolidine ring was guanylated. Through experimentation, the Ma group found that the most efficient guanylation agent was *N*-Boc-protected methylisothiourea **16**. Since elevated temperatures led to decomposition of the triamine **6**, silver nitrate was used to promote the substitution of methanethiol in the guanylation reaction. By changing the solvent from acetonitrile to a mixture of acetonitrile and methanol (2:1), the yield for the reaction could be improved from 20% to 65%. This effect was most likely due to the poor solubility of triamine **6** in acetonitrile. The methyl ester **17** was finally converted to martinellic acid ((−)-**2**), as the TFA salt in 91% yield through base catalyzed hydrolysis of the ester and subsequent treatment with TFA.

**Scheme 3:**
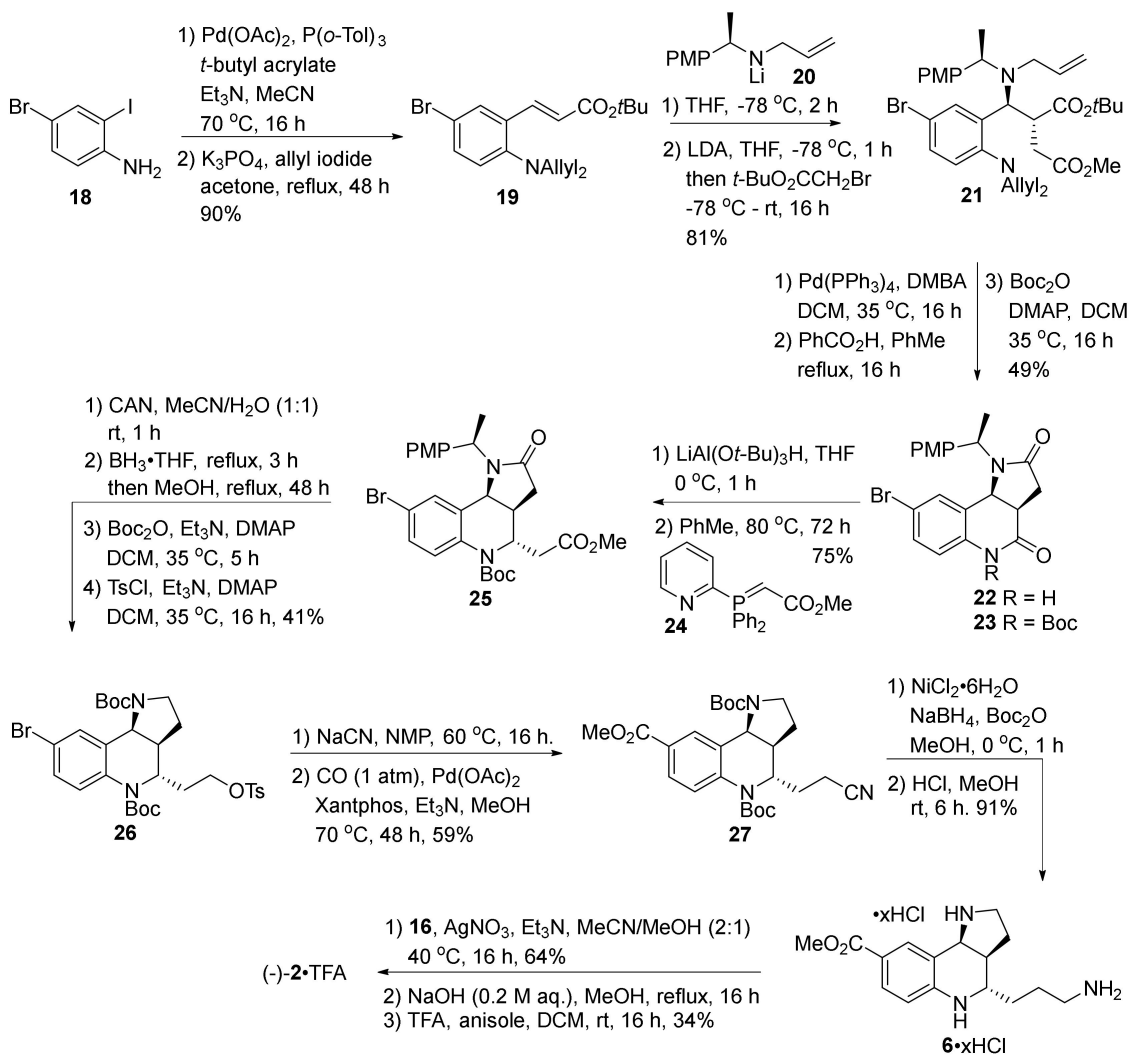
The Davies group’s synthesis of (−)-martinellic acid (**2**).

In 2013, Davies and co-workers published their own synthesis of (−)-martinellic acid ((−)-**2**) and (−)-*epi*-martinellic acid ((−)-*epi*-**2**) (Scheme 3) [32,40]. The synthesis began with a Heck cross-coupling between 2-iodo-4-bromoaniline **18** and *tert*-butyl acrylate, followed by bis-*N*-allyl protection, which provided ester **19** in 90% yield. Conjugate addition of (*R*)-lithium amide **20** to α,β-unsaturated ester **19** by means of a diastereoselective *aza*-Michael reaction was used to install the C-10 stereogenic center. Subsequent alkylation with methyl bromoacetate gave compound **21** as a single diastereomer.

In preliminary studies, the Davies group attempted to form the pyrrolidine ring through a free amine (N-10a) by conducting a hydrogenolysis of all *N*-protecting groups. Neither acid promoted reaction of the free amine with *tert*-butyl ester nor did the methyl ester provide the desired five-membered cyclized product. However, upon removal of only the *N*-allyl groups with Pd(PPh_3_)_4_ and *N*,*N*-dimethylbarbituric acid (DMBA), followed by benzoic acid promoted cyclization of both rings, simultaneously, pyrroloquinolinone **22** was obtained. In order to further regioselectively reduce the six-membered lactam over the five membered lactam moiety in compound **22**, the electron withdrawing Boc-protecting group was introduced onto the N-8a nitrogen. The activated carbonyl group in compound **23** was then reduced with lithium tri-*t*-butoxyaluminum hydride LiAl(O*t*-Bu)_3_H to form the hemiaminal. Treating the hemiaminal with phosphorane **24** allowed for olefination and an intramolecular *aza*-Michael addition to give compound **25** in 75% yield as a single diastereomer. Phosphorane **24** was used in the olefination reaction instead of the corresponding Wittig reagent due to issues with separating the reaction product from triphenylphosphine ylide residues.

The *p*-methoxyphenyl (PMP)-protection group within compound **25** could be removed upon treatment with ceric ammonium nitrate (CAN). The resulting substrate was then treated with borane (BH_3_) to reduce the amide and ester functionalities to the corresponding amine and alcohol moieties, respectively. The amine was Boc-protected, and the alcohol was tosylated, which provided pyrroloquinoline **26** in 41% yield over four steps. Treatment of compound **26** with sodium azide in *N*-methyl-2-pyrrolidinone (NMP) followed by methoxycarbonylation provided methyl ester **27**. The nitrile group in compound **27** was then reduced to the corresponding amine upon treatment with sodium borohydride, and the resulting product was immediately Boc-protected in order to avoid formation of a tetracycle. Deprotection of the three *N*-Boc groups with methanolic HCl provided Ma’s intermediate (**6**•*x*HCl) in 91% yield. (−)-Martinellic acid ((−)-2•TFA) was finally obtained from the salt 6•*x*HCl in 49% yield using Ma’s guanylation procedure [37].

**Scheme 4:**
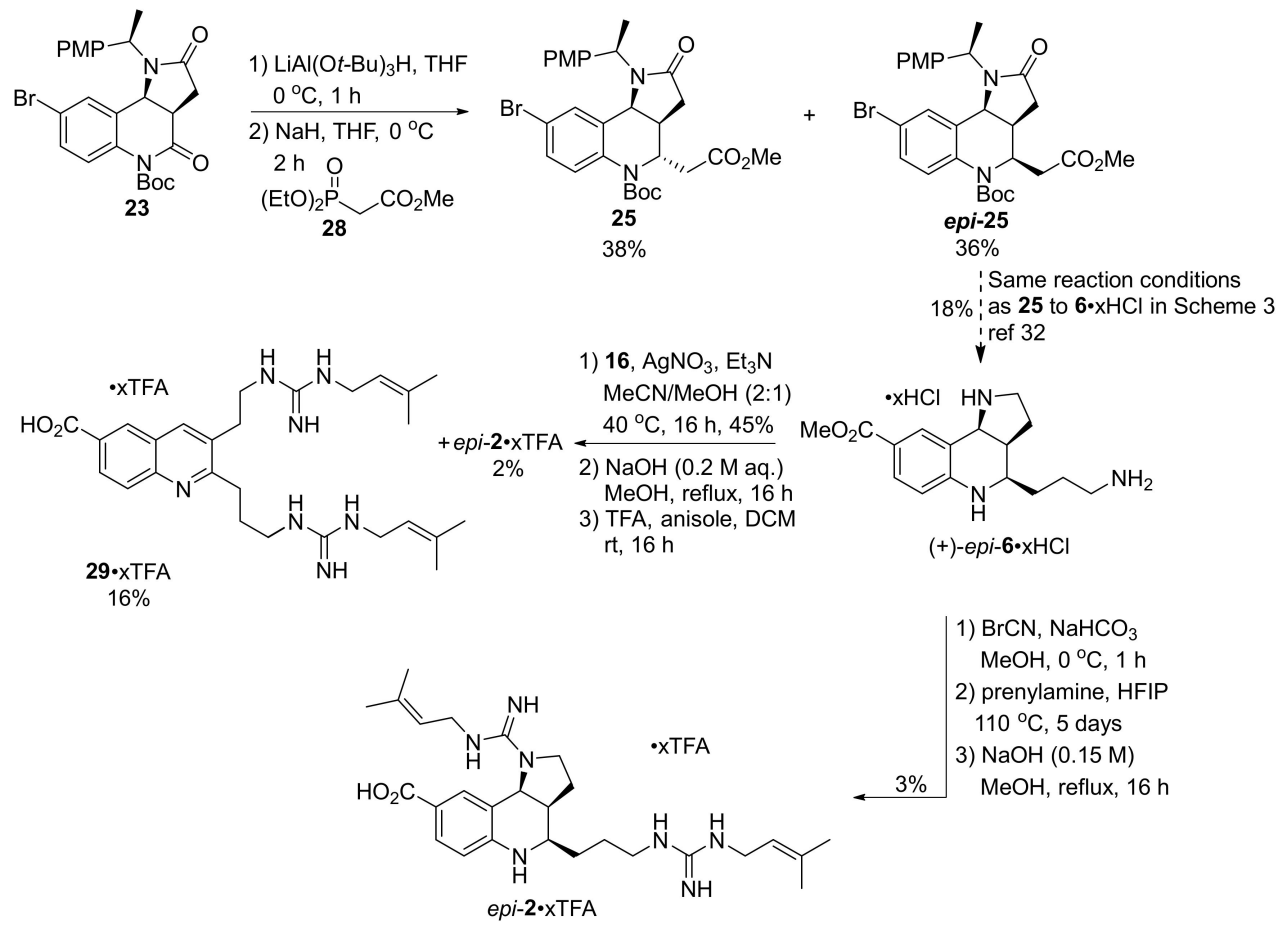
The Davies group’s synthesis of *epi*-(−)-martinellic acid (epi-(−)-**2**).

Initial studies by Davies and co-workers showed that a strong base, such as NaH, led to equilibration between diastereomers **25** and epi-**25** (Scheme 4). Since this would provide access to the C(4)-epimer of martinellic acid 2•*x*HCl, the Davies group also synthesized compound (+)-epi-6•*x*HCl by an analogous series of steps to the ones used for the synthesis of Ma’s intermediate (−)-6•*x*HCl from pyrroloquinoline **25**, in 18% overall yield (Scheme 3). The corresponding guanylated product was provided in 45% yield by following Ma’s protocol [37]. However, hydrolysis of the methoxy ester gave (−)-4-epi-Martinellic acid epi-2•*x*TFA in only 2% yield, accompanied by quinoline 29•*x*TFA in 16% yield. By using the procedure reported by Snider et al., epi-martinellic acid (epi-2•*x*TFA) was isolated in 3% yield over the three steps, and no formation of the quinoline 29•*x*TFA was observed [45].

**Scheme 5:**
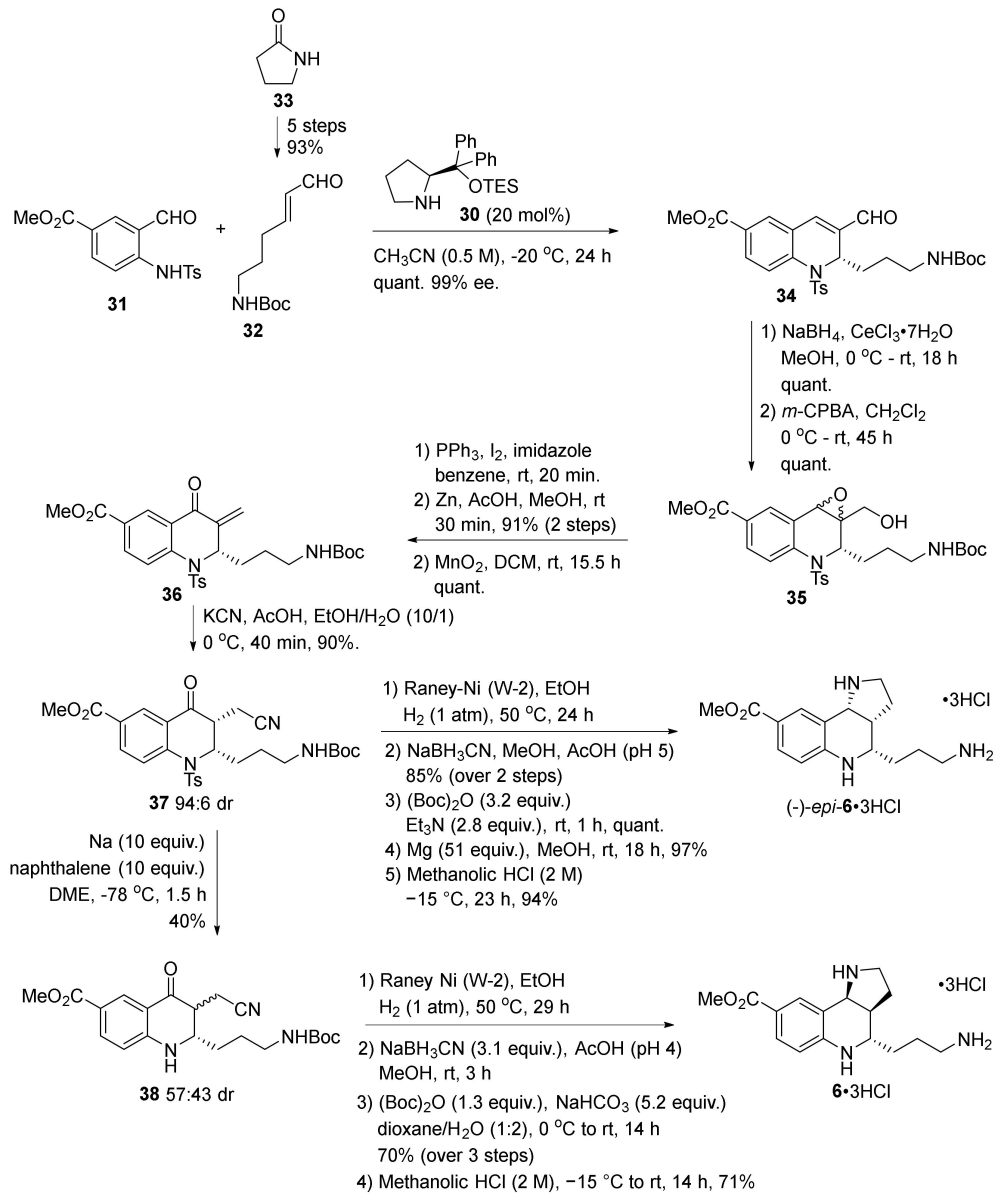
Hamada and coworkers’ synthesis of Ma’s intermediate (**6**).

The Hamada group employed a previously reported asymmetric tandem Michael–aldol reaction using (*S*)-diphenylprolinol triethyl silyl ether **30** as an organocatalyst in the presence of sodium acetate (NaOAc) and four Å molecular sieves (4 Å MS) as starting point for their synthesis [42,49]. During optimization studies for the reaction between substrates **31** and **32** (obtained in 5 steps from pyrrole **33**), the group found that when performed in acetonitrile at −20 °C, the reaction proceeded smoothly without NaOAc and 4 Å MS (Scheme 5). By using two equivalents of the alkene **32**, due to slow reaction rate, together with 20 mol% of organocatalyst **30**, quinoline **34** was obtained in quantitative yield from compound **31** and high enantiomeric excess. The aldehyde moiety in compound **34** was further reduced to the allyl alcohol with sodium borohydride and cerium chloride.

After several failed attempts to introduce the N-8a nitrogen to the allylic alcohol, Hamada employed a route to Ma’s intermediate (**6**) previously reported in their group [50]. The allylic alcohol was treated with *m*-chloroperbenzoic acid (*m*-CPBA) to form epoxide **35**. Iodination of compound **35** and subsequent treatment with zinc and acetic acid to cleave the epoxide ring gave an allylic alcohol that was further oxidized to ketone **36** with activated manganese dioxide. The α,β-unsaturated ketone **36** was subjected to hydrocyanation to provide quinolone **37** in 90% yield and 94:6 dr. Only after formation of (−)-epi-Ma’s intermediate ((−)-epi-**6**) through a series of reduction and *N*-deprotection reactions could Hamada and co-workers conclude that cyano-quinolone diastereomer **37** exhibited cis-configuration. In their 2004 publication the Hamada group reported that the “2,3-trans arrangement was ambiguously confirmed by NMR” [50].

Base-promoted racemization of the cyano-sidechain with sodium naphthalenide and simultaneous *N*-tosyl-deprotection produced compound **38** as a diastereomeric mixture. The mixture was subjected to hydrogenation and reductive amination of the resulting imine followed by *N*-Boc protection. A final deprotection with methanolic HCl formed (−)-Ma’s intermediate as its HCl salt ((−)-**6**•3HCl). Hamada and co-workers hypothesized that the *N*-protecting group (Ts) controls the *cis*/*trans* isomer formation during cyanation, through a pseudo-1,3-diaxial interaction between the *N*-toluenesulfonyl group and 3-cyanomethylone. Without the tosyl group, they imagined that the isomerization of the 3-cyanomethyl group takes place during the hydrogenation reaction, and that the trans-product **6** is more stable due to torsional strain between the 2-(3-*tert*-bytoxycarbonylaminopropyl) group and the pyrrolidine ring in a *cis*-product.

**Scheme 6:**
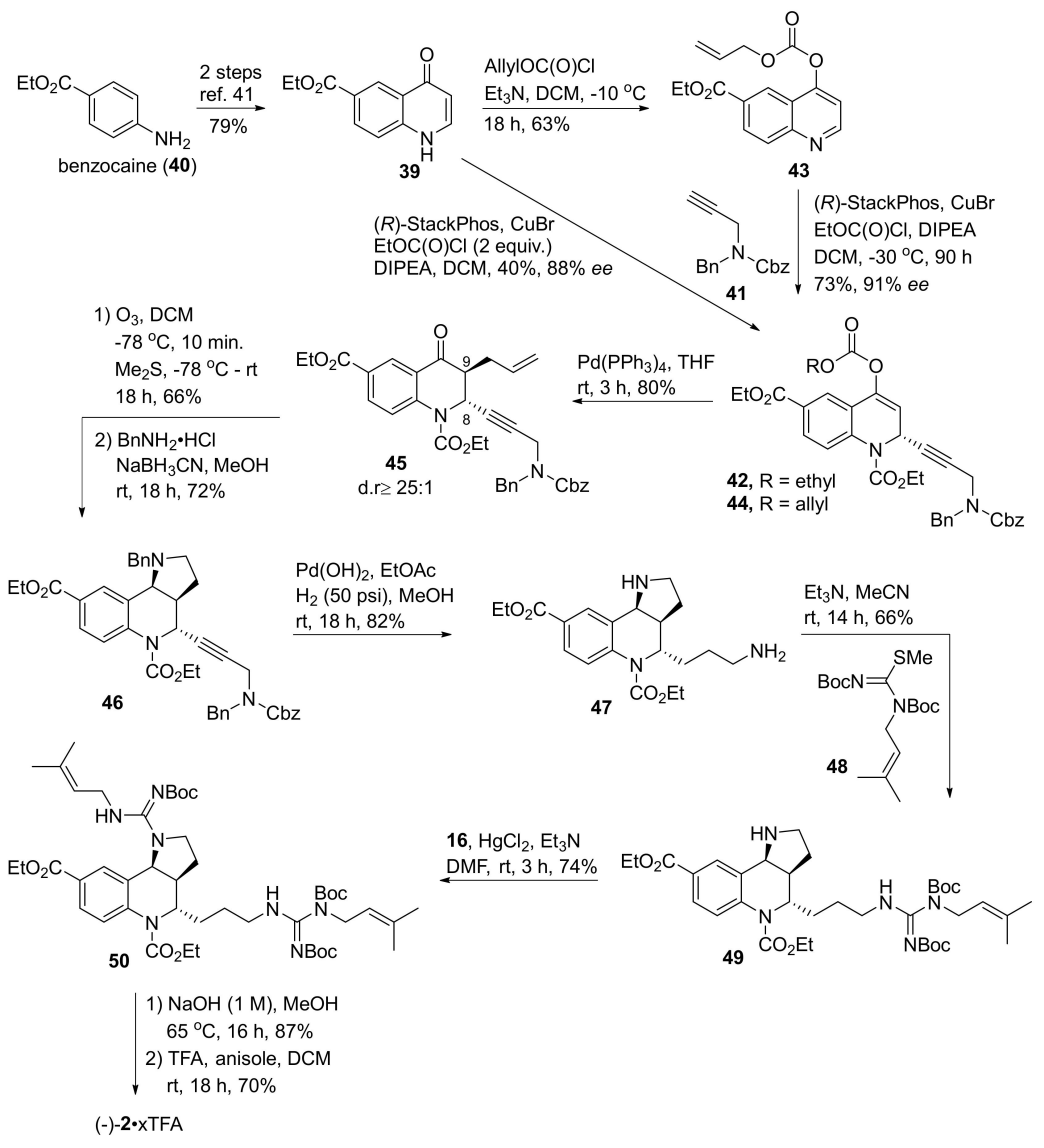
Synthesis of (−)-martinellic acid ((−)-**2**•*x*TFA) by Pappoppula and Aponick.

Another approach towards martinellic acid was presented by Pappoppula and Aponick in 2015 [51]. Their synthesis commenced from quinoline **39**, which was synthesized in two steps from benzocaine (40) in 79% yield following a literature procedure (Scheme 6) [52]. The key step in this synthesis was a copper-catalyzed alkynylation reaction with alkyne **41**, developed in the group, using (*R*)-StackPhos to set the C-8 stereochemistry. Their further strategy was to use an α-allylation reaction to diastereoselectively install the allyl group at C-9 [53]. To facilitate that chemistry, they therefore needed to convert ketone **39** to an aromatic enol.

In preliminary studies conducted by Pappoppula and Aponick, they wanted to see if it was possible to obtain alkyne **42** directly from quinoline **39**, by forming the corresponding carbonate in situ. This route gave the alkynylated product **42** in 40% yield and 88% ee. Attempts to increase the yield were, however, unsuccessful, because once the carbamate was formed, the quinolone did not undergo further alkynylation, resulting in the formation of an unreactive biproduct, viz. *N*-protected quinoline. Instead, the group prepared allylcarbonate **43** by treating quinolone **39** with alloc chloride. Stereoselective alkynylation of carbonate **43** at 0 °C for 15 h using (*R*)-StackPhos provided alkyne **44** in 95% yield and 86% ee. By running the reaction at −25 °C for 48 h and increasing the reaction concentration from 0.1 to 0.4 M, the title compound **44** was obtained in 90% ee, however at a lower conversion (70% yield). When scaling up the reaction, the copper bromide catalyst loading could be reduced from 5 to 2 mol% without affecting the yield and stereoselectivity, providing alkyne **44** in 73% yield and 91% ee. Diastereoselective decarboxylative α-allylation of allyl carbonate **44** further provided ketone 45 in 80% yield with a >25:1 diastereomeric ratio. Allyl **45** was then treated with ozone followed by a double reductive amination of the resulting 1,4-diacarbonyl using benzyl amine and sodium cyanoborohydride to provide the tricycle **46**. Reduction of the alkyne functionality upon treatment with Pd(OH)_2_ and hydrogen (50 psi) while simultaneously removing three *N*-protecting groups resulted in the formation of amine **47** in 82% yield. Treating amine 47 with the di-Boc protected reagent **48** resulted in guanidinylation of the primary amine and formation of compound **49**. The sterically hindered secondary amine was then guanidinylated with the more reactive guanidine **16** to provide compound **50** in 74% yield. Finally, the TFA salt of martinellic acid ((−)-2•*x*TFA) was obtained upon base catalyzed ethyl ester hydrolysis followed by deprotection of the *N*-Boc groups with TFA.

**Scheme 7:**
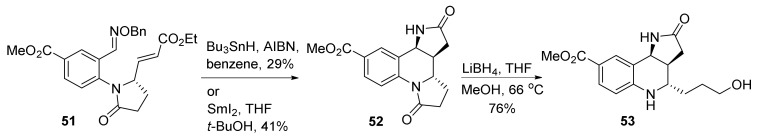
The key step in the Naito group’s first approach to (−)-martinellic acid ((−)-**2**), namely the radical addition-cyclization-elimination (RACE) reaction.

In 2008, Naito and co-workers presented the synthesis of (−)-martinellic acid ((−)-**2**), where the key reaction was a radical addition–cyclization–elimination (RACE) reaction with a chiral oxime ether **51** forming lactam **52**. Lithium borohydride reduction of lactam **52** afforded pyrroloquinoline **53** (Scheme 7), which was brought forward to (−)-martinellic acid ((−)-2) over seven steps [31]. A full report of this method has been covered in the review by Lovely and Bararinarayana [34]. The approach unfortunately experienced some unsatisfactory yields and stereoselectivity, which prompted a second approach to martinellic acid.

**Scheme 8:**
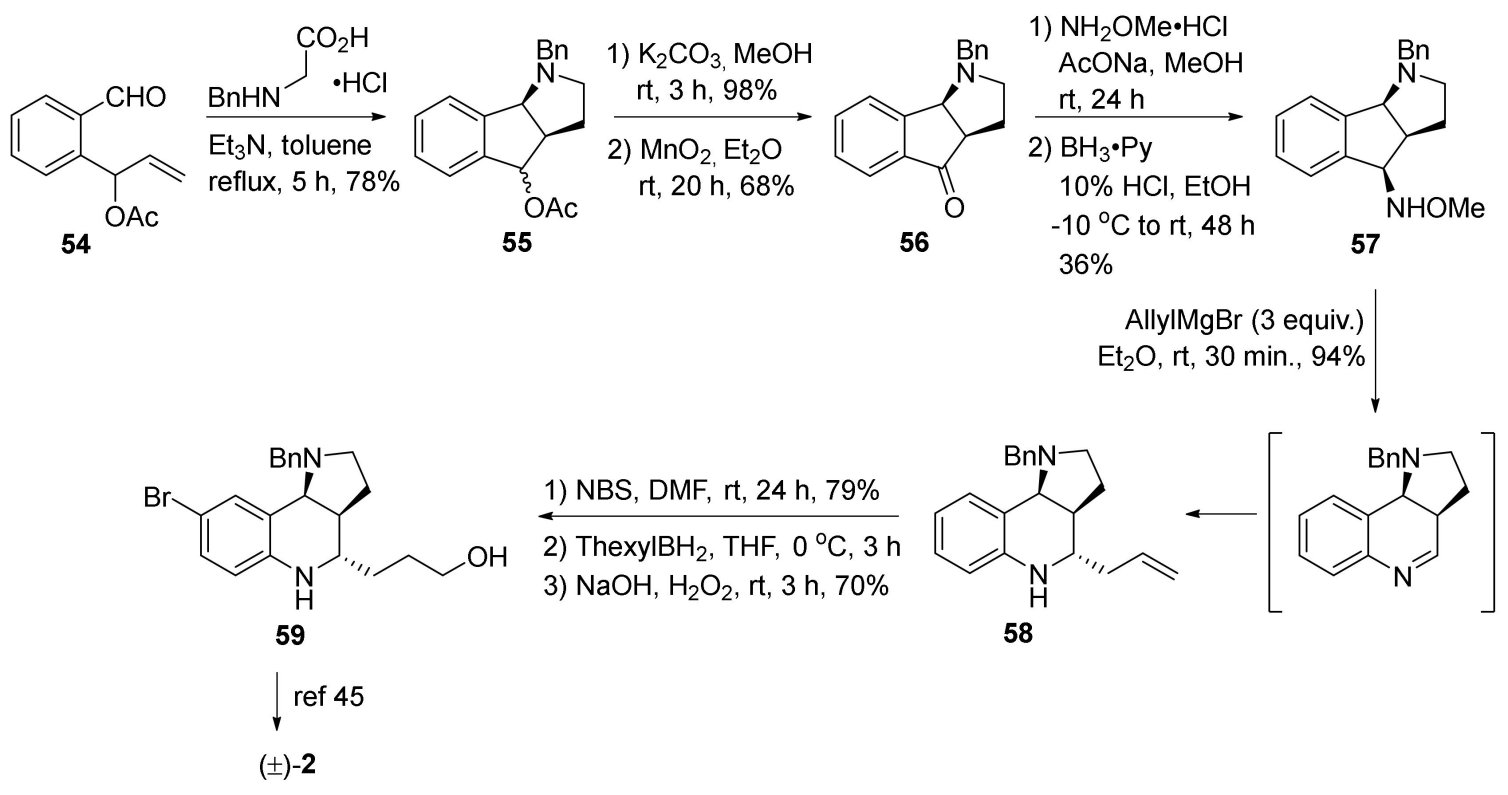
The Naito group’s second approach to martinellic acid in collaboration with Miyata.

A formal synthesis of (±)-martinellic acid was further reported by the Naito group together with Miyata in 2010 using a new approach based on ring expansion of an oxime ether through a domino reaction, including elimination of an alcohol, rearrangement of a metal aryl methylamide, and addition of an organomagnesium reagent (Scheme 8) [54].

The work commenced by the condensation of aldehyde **54** with *N*-benzylglycine hydrochloride followed by a spontaneous [3+2] cycloaddition of the resulting azomethine ylide gave indenopyrrolidine **55** in 78% yield [54]. Acetate hydrolysis and manganese dioxide (MnO_2_) oxidation of the resulting alcohol provided ketone **56**. Condensation with *O*-methylhydroxylamine hydrochloride followed by borane reduction gave oxime ether **57** in 36% yield. A domino reaction with allylmagnesium bromide proceeded stereoselectively from oxime ether **57** to afford amine **58** in 94% yield. Bromination was followed by hydroboration-oxidation of the alkene. The resulting pyrroloquinoline **59** is an intermediate in the synthesis of (±)-martinellic acid reported by Snider et al. [45], in which compound **59** was converted to (±)-martinellic acid over six steps.

**Scheme 9:**
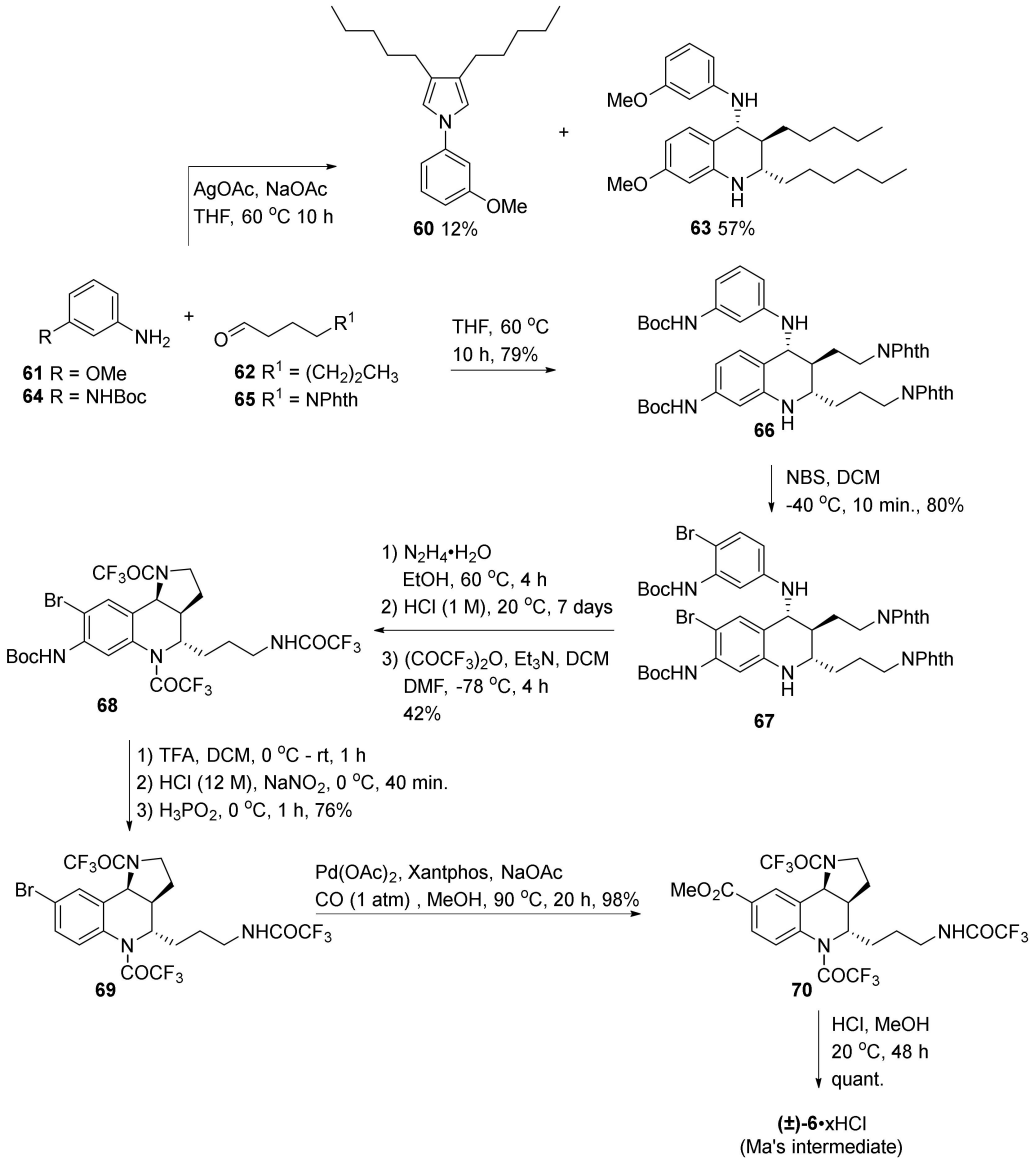
Jia and coworkers’ approach to (±)-Ma’s intermediate ((±)-6•*x*HCl).

During their synthetic studies of poly-substituted pyrroles such as pyrrole **60** from amine **61** and aldehyde **62** using silver acetate (AgOAc) as oxidant, Jia and co-workers unexpectedly observed formation of quinoline **63** (Scheme 9) [41]. They proposed that formation of the quinoline **63** occurred in a Povarov reaction.

On further inspection the Jia group found that the reaction proceeded in THF at elevated temperature without any additional reagents, in high yield and high stereoselectivity with various R groups. To utilize this reaction in the synthesis of Ma’s intermediate (**6**), *N*-Boc protected amine **64** was reacted with *N*-phthaloyl protected aldehyde **65** to give quinoline **66** in 79% yield. In order to attach a handle for installing the methyl ester in Ma’s intermediate (**6**), the quinoline **66** was treated with *N*-bromosuccinimide (NBS) for 10 min to form bromide **67** in 80% yield.

*N*-phthaloyl groups were removed from bromide **67** upon treatment with hydrazine hydrate followed by treatment with HCl to facilitate ring closure of the five-membered ring. The resulting pyrroloquinoline was *N*-protected with trifluoroacetic anhydride to provide compound **68**. The bromo quinoline **69** was obtained by *N*-Boc deprotection followed by reductive removal of the resulting amine. Carbonylation of the bromo quinoline **69** to form the corresponding methyl ester **70**, followed by *N*-COCF_3_ deprotection with HCl, afforded (±)-Ma’s intermediate ((±)-6•*x*HCl).

## 3. Tricyclic Core Scaffold Synthesis

**Scheme 10:**
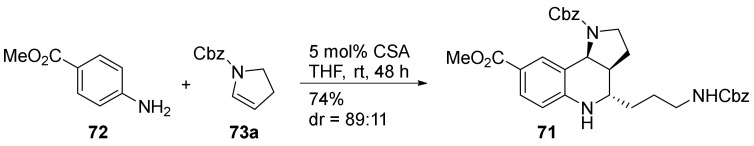
Scaffold assembly by Batey and Powell.

**Scheme 11:**
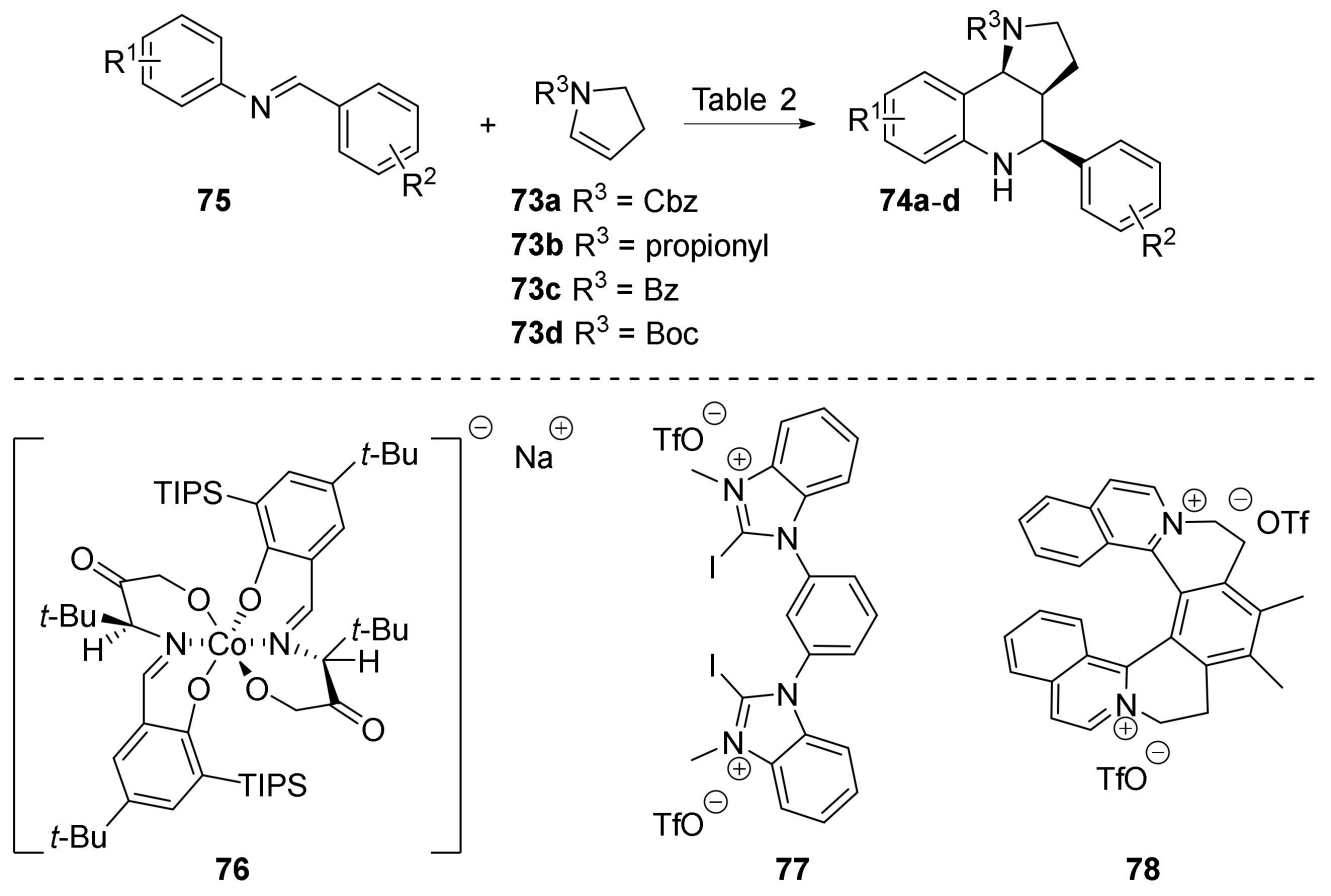
Formation of 8-phenyl pyrroloquinoline **74a**–**d** in the Povarov reaction between the aromatic imine **75** and enamide **73a**–**d**. For more information, see Table 2.

In addition to synthetic preparation of the martinella alkaloids the synthesis of the tricyclic core of the natural products has also attracted significant attention. To this day, no studies of how the hexahydropyrrolo[3,2-c]quinoline structure has arisen biosynthetically in nature have been published. However, in their synthesis of the martinella alkaloids, Batey and Powell put together the tricyclic core structure **71** in an acid catalyzed two-component Povarov reaction between aniline **72** and enamide **73a** (Scheme 10) and suggested that the martinella alkaloids may be naturally assembled in a related biosynthetic process [38,55]. The Povarov reaction (also known as the aza-Diels Alder reaction) has been a prevalent method for synthesizing *N*-heterocycles [56], including the pyrroloquinoline core structure [57]. Particularly, the assembly of the scaffold **74** from the aromatic imine **75** and the enamide **73** has been a well-studied reaction (Scheme 11) and will be firstly discussed in this section. However, other strategies have also been developed for the construction of the pyrroloquinoline scaffold, and these syntheses will further be presented in chronological order.

The formation of the 8-phenyl-pyrroloquinoline **74** from the Povarov reaction between aromatic imine **75** and *N*-protected enamide **73** has been a popular reaction for constructing the pyrroloquinoline scaffold (Scheme 11, Table 2). The reaction was first reported by Hadden and Stevenson in 1999 [58]. They enabled the reaction with indium chloride to obtain the pyrroloquinoline **74** in a 1:1 mixture of endo/exo isomers (Table 2, Entry 1). The use of a 2-nitro phenyl aromatic imine **75** in the reaction favored formation of the endo-product **74b** (Table 2, Entry 2). The endo-compound **74c** was also obtained from the zinc triflate catalyzed Povarov reaction conducted by Wang and co-workers (Table 2, Entry 3) [59], as well as from the cobalt catalyzed reaction employing catalyst **76,** presented by Gong and co-workers (Table 2, Entry 4) [60]. Other catalysts that have been explored for this reaction are the iodo catalyst **77** (Table 2, Entry 5) and helquat catalyst **78** (Table 2, Entry 6), both of which facilitated formation of approximately 1:1 mixtures of endo/exo isomers [61,62]. Synthesis of a DNA-barcoded library that included the pyrroloquinoline scaffold has also utilized the Povarov reaction to assemble the scaffold **74d**, using a micellar sulfonic acid to catalyze the reaction [63].

**Scheme 12:**
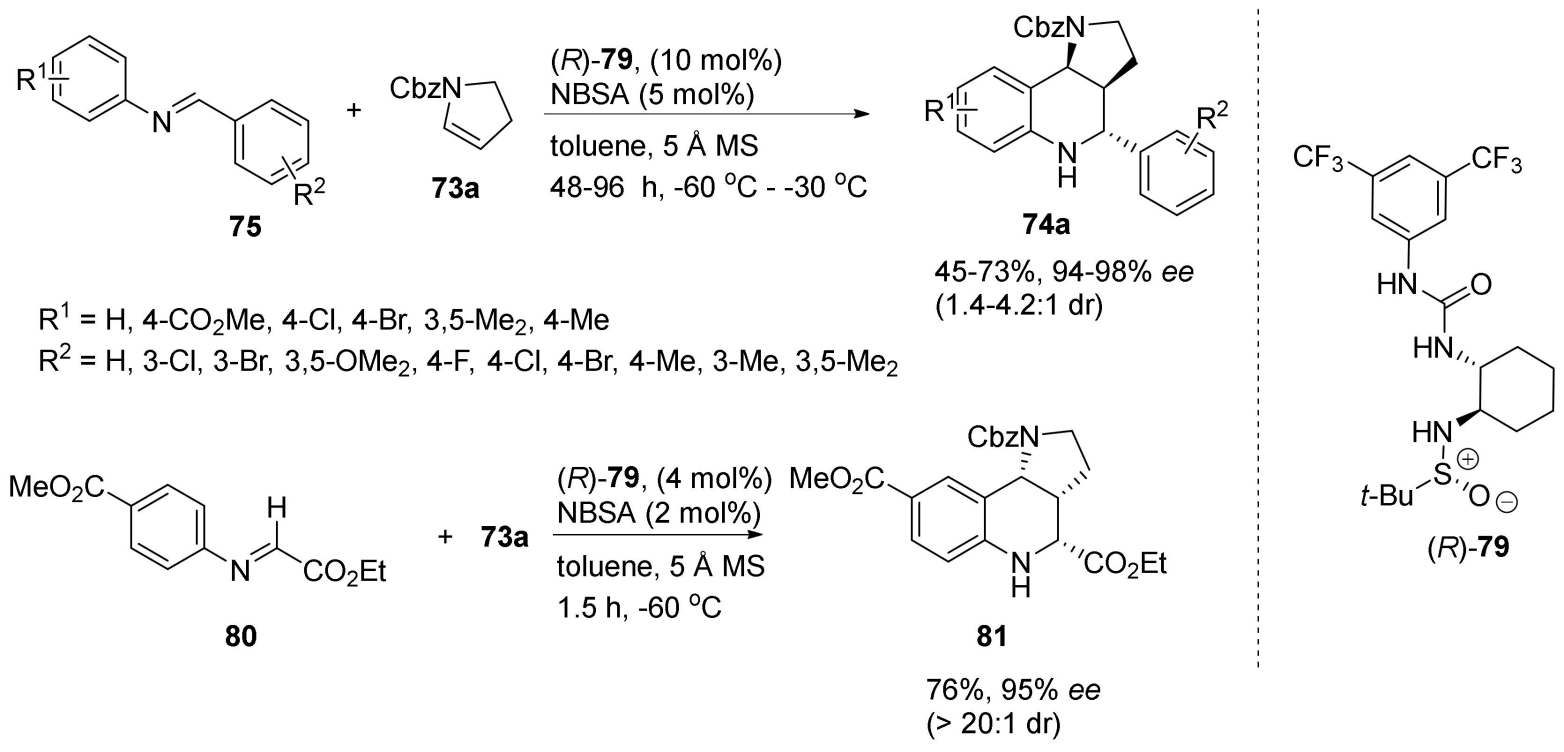
Organocatalytic scaffold synthesis developed in the Jacobsen group.

The Jacobsen group produced the martinella scaffold in their experimental and computational study of Brønsted-acid promoted asymmetric Povarov reactions between *N*-aryl imines and electron rich alkenes [64]. They explored an anion-pathway for catalysts, such as urea (*R*)-**79**, in which the catalyst binds to the positively charged substrate through its counterion. They further showed that both the urea and sulfonyl functionalities where required for the catalyst **79** to facilitate the Povarov reaction. The exo-8-phenyl pyrroloquinoline **74a** was obtained as the major stereoisomer (94–98% ee) in the reaction between *N*-aryl imines **75** and enamide **73a** in the presence of urea (*R*)-**79** and ortho-nitrobenzene sulfonic acid (NBSA) (Scheme 12). Stereoselectivity was flipped when reacting glyoxylate imine **80** with the enamide **73a** in analogous conditions. In this case, the endo-isomer **81** was obtained as the major product in 95% enantiomeric excess.

**Scheme 13:**
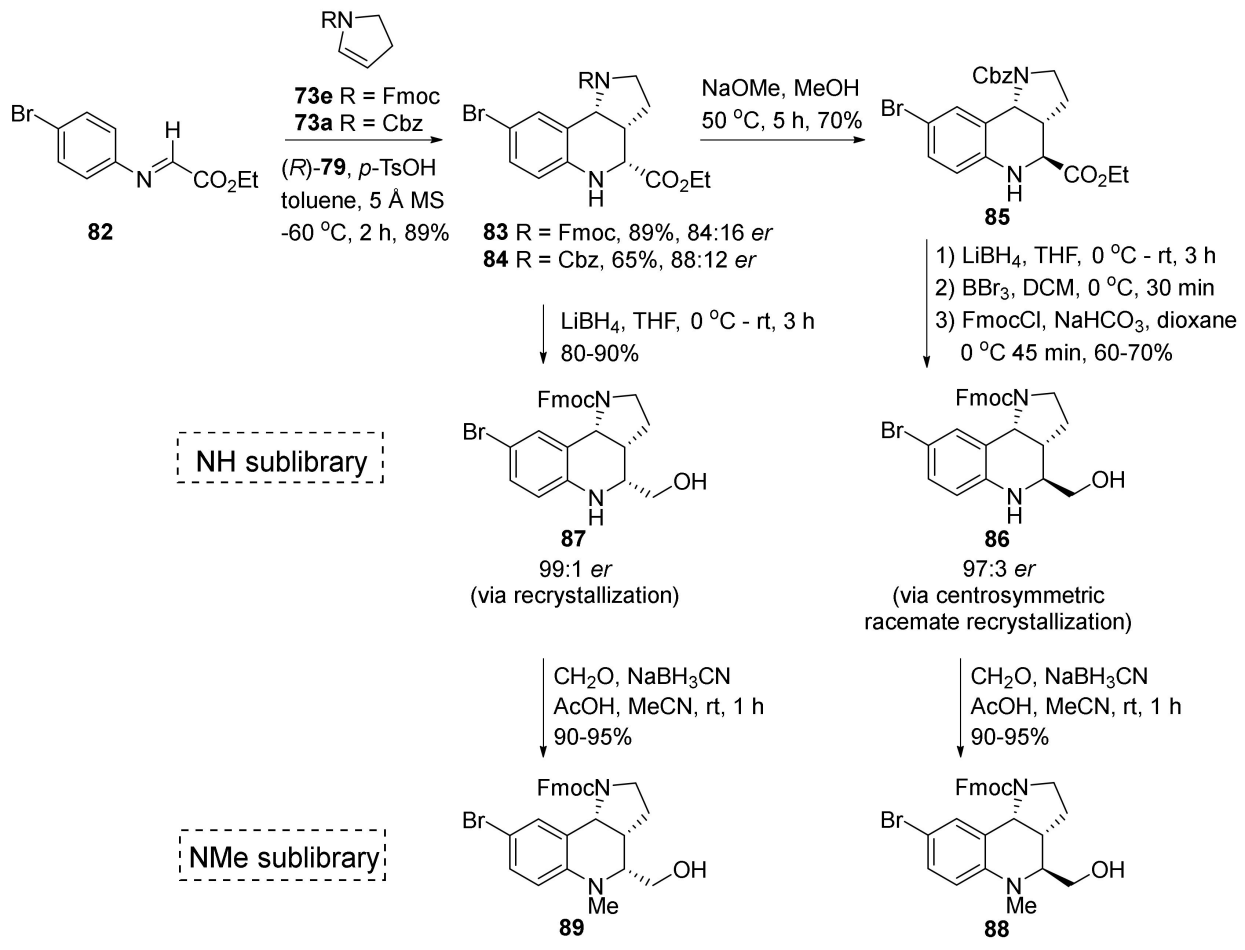
Large scale scaffold synthesis by Marcaurelle and co-workers.

**Scheme 14:**
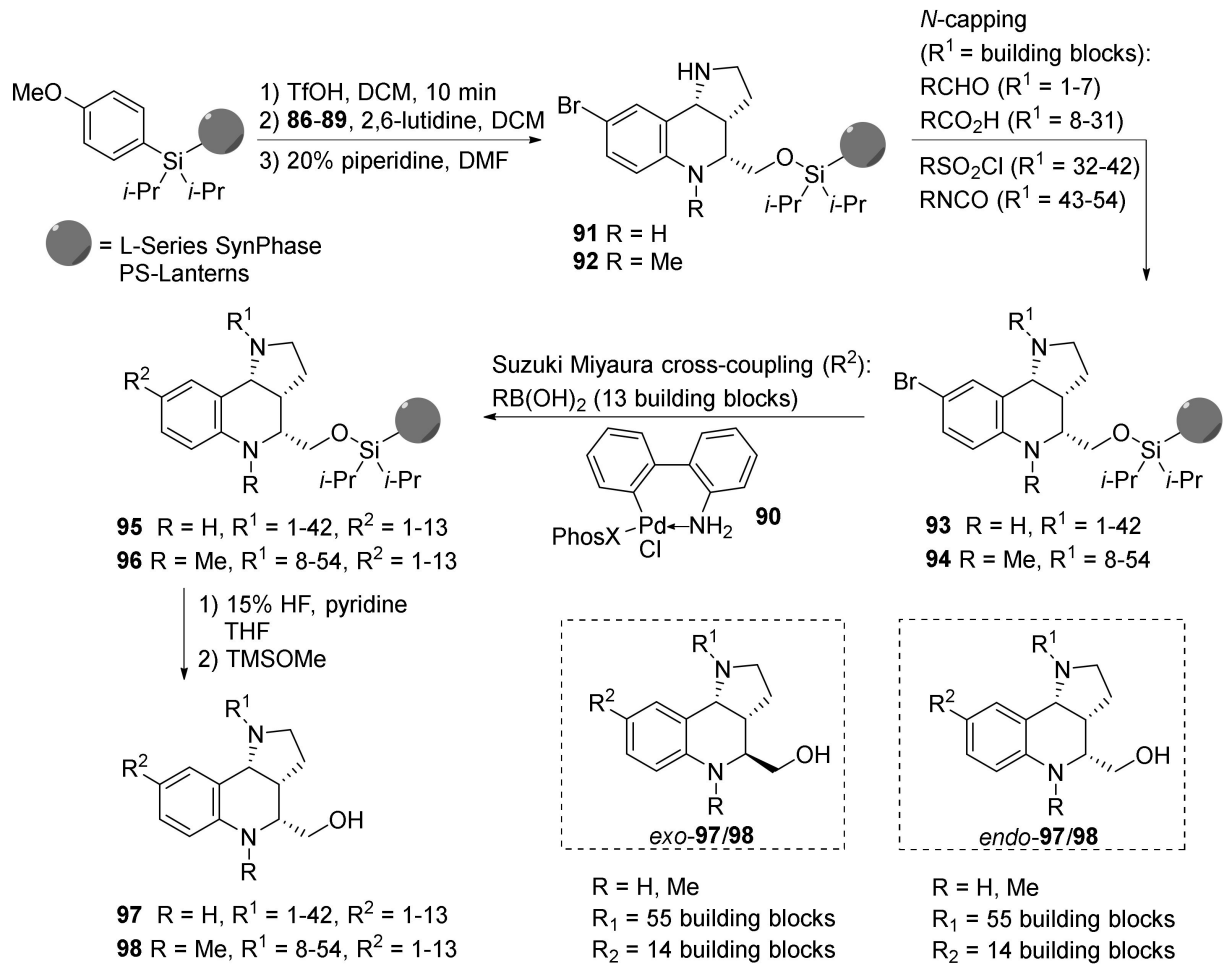
Solid support scaffold diversification by Marcaurelle and co-workers.

The synthetic method developed in the Jacobsen laboratory (Scheme 12) was further utilized together with Marcaurelle and co-workers to set up a 2328-membered library consisting of four pyrroloquinoline stereoisomers flanked with a variety of side chains and functional groups for a Stereo/Structure–Activity–Relationship (SSAR) study [65]. The library was assembled in two consecutive parts, namely, asymmetric synthesis of the four possible pyrroloquinoline stereoisomers (Scheme 13) followed by a solid phase synthesis for further diversification of the pyrroloquinoline scaffold (Scheme 14).

The pyrroloquinoline scaffold was synthesized via the urea **79** catalyzed Povarov reaction between aromatic imine **82** and dihydropyrrolidine **73a** or **73e** (Scheme 13). Fmoc- and Cbz-protected pyrroloquinolines **83** and **84** were obtained in high yield and enantiomeric ratio. The imine **82** tended to hydrolyze when the reaction was run at larger scale. To solve this hydrolysis issue, the Brønsted acid NBSA initially used in the Jacobsen group was replaced by anhydrous *p*-toluene sulfonic acid (*p*-TsOH) when the reaction was conducted on gram scale.

The tricycle mirror images ent-**83** and ent-**84** were obtained in the presence of urea catalyst (*S*)-**79**. To include all possible stereoisomers in the study, the group conducted an epimerization of the *N*-Cbz protected endo-isomers **84** and ent-**84**, employing sodium methoxide in methanol to afford the more thermodynamically stable exo-products **85** and ent-**85**. Reduction of the ester group in compound **85** with lithium borohydride followed by protecting group exchange from Cbz to the base labile Fmoc, a protecting group that was compatible with the following solid phase library synthesis, resulted in the formation of alcohol **86**. The endo-pyrroloquinoline **83** was reduced with lithium borohydride to afford the corresponding alcohol **87**. The *N*-Fmoc protected pyrroloquinolines **86** and **87** could be recrystallized to afford the enantiopure compounds in up to 97:3 and 99:1 er, respectively.

The quinoline amine in compounds **86** and **87** was the first site for the planned diversification of the scaffold. Marcaurelle and co-workers found that this secondary amine could be alkylated with only a limited number of aldehydes. The four enantiomers **86**/ent-**86** and **87**/ent-**87** were therefore *N*-alkylated prior to the solid phase diversification (Scheme 14), in a reductive amination reaction with formaldehyde, to afford *N*-methyl pyrroloquinolines **88/**ent-**88** and **89**/ent-**89**. This first step of diversification afforded two groups of compounds that consisted of amines **86**/ent-**86** and **87**/ent-**87** and methylamines **88**/ent-**88** and **89**/ent-**89**, which were referred to as the NH and NMe sublibraries, respectively.

All four stereoisomers of the NH and NMe sublibraries were further diversified by amine capping of the secondary pyrrolidine amine and cross-coupling of the aryl bromide moiety with selected building blocks (Scheme 14). To find appropriate building blocks for the amine capping, Marcaurelle and co-workers firstly conducted a feasibility study with 54 pre-selected substituents, including sulfonyl chlorides, isocyanates, carboxylic acids, and aldehydes. They then observed bis-capping of the NH sublibrary in the presence of isocyanates, and decomposition in the NMe sublibrary during reductive amination with aldehydes. Therefore, isocyanates (building blocks 43–54) were removed from the NH sublibrary design and aldehydes (building blocks 1–7) were removed from the design of the NMe sublibrary. This was followed by a feasibility study of palladium cross-coupling reactions. Since the Sonogashira cross-coupling conditions led to poor conversion, Suzuki-Miyaura cross-coupling conditions were further explored. A variety of ligands ((P(*t*-Bu)_3_), PEPPSI, and *S*-Phos), palladium-catalysts (PdCl_2_(PPh_3_)_2_ and Pd(dba)_2_), bases (TEA, K_3_PO_4_, and CsCO_3_), and solvents (EtOH, PhMe, 1,4-dioxane, and THF) were tested in order to minimize byproduct formation in the Suzuki–Miyaura cross-coupling reaction. Finally, appreciable conversion to the coupling products was achieved by using the Buchwald precatalyst **90** [66].

Based on the feasibility study, a virtual library including all possible combinations of a master list of building blocks was constructed, generating approximately 2400 compounds for each stereoisomer. A filter that excluded building block combinations with undesirable physicochemical properties (desirable properties: MW ≤ 500, ALogP −1 to 5, H-bond acceptors + donors ≤10, rotatable bonds ≤10 and TPSA ≤ 140) was then applied to the dataset. This finally brought together a library of 274 compounds for each of the NH scaffolds and 310 compounds for each of the NMe scaffolds.

The 2328-membered library was finalized in a solid support synthesis (Scheme 14). The SynPhase Lanterns were activated by triflic acid (TfOH), and alcohols 86–89 were then loaded onto the solid support in the presence of 2,6-lutidine. Removal of the *N*-Fmoc group with piperidine in DMF afforded amines **91** and **92**, which were subjected to *N*-capping with the 54 pre-selected building blocks. Suzuki–Miyaura cross-coupling of the resulting bromoanilines **93** and **94** with 14 different boronic acid building blocks in the presence of the Buchwald catalyst **90** afforded coupling products **95** and **96** [66]. The diversified products **95** and **96** were subsequently liberated from the solid support upon treatment with HF and pyridine to afford compounds **97** and **98**. Analysis of the finalized library confirmed that the compounds exhibited suitable physicochemical properties for further biological testing. For this purpose, Marcaurelle and co-workers submitted the diversified pyrroloquinoline library to the NIH molecular library small molecule repository (NIH MLSMR).

**Scheme 15:**
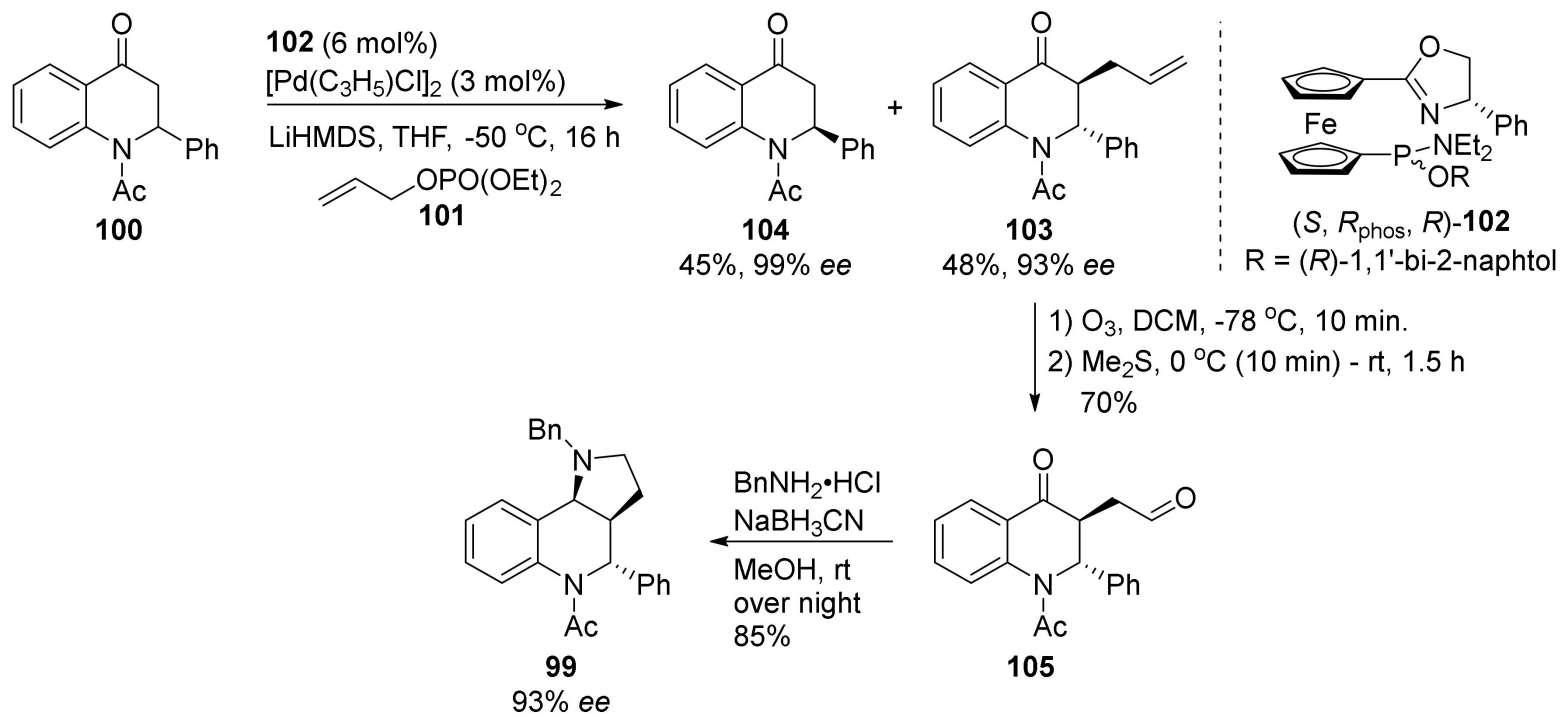
Scaffold synthesis by Hou and co-workers.

Hou and co-workers obtained the exo-diastereomer of phenyl-pyrroloquinoline **99** in 93% ee by kinetic resolution (Scheme 15) [67]. Quinoline **100** was subjected to a palladium catalyzed asymmetric allylic alkylation reaction with 50 mol% of allyl reagent **101** and 6 mol% of catalyst **102** in the presence of lithium bis(trimethylsilyl)amide (LiHMDS). These conditions favored formation of the *trans*-enantiomer **103**, which at the same time enabled recovery of the close-to enantiopure hydroquinolone **104**. Alkene **103** was further converted to the pyrroloquinoline **99** by first performing an ozonolysis to afford aldehyde **105**, which was followed by a double reductive amination reaction with benzylamine and sodium cyanoborohydride as reducing agent to provide the tricyclic scaffold **99** in 85% yield.

**Scheme 16:**
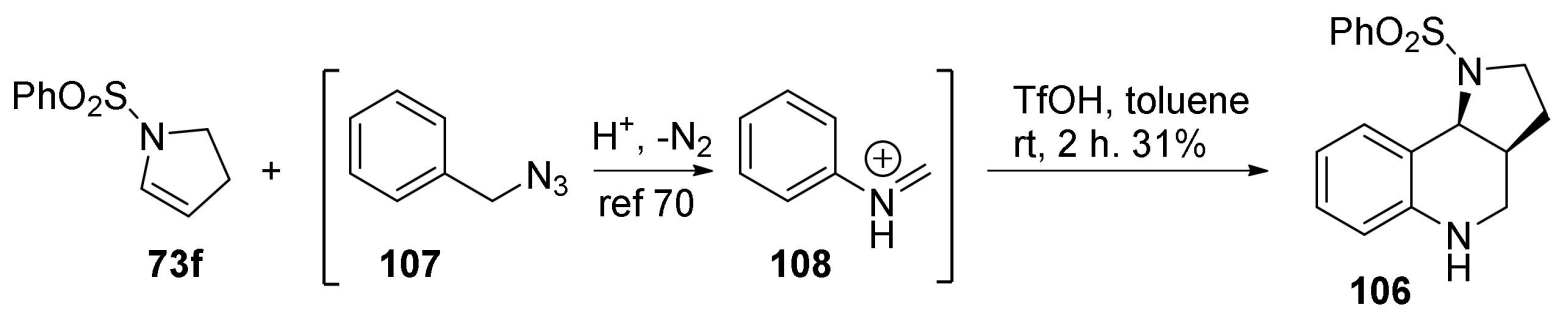
Scaffold synthesis by Gilliaizeau and Gigant.

Gilliaizeau and Gigant synthesized a variety of nitrogen-fused tetrahydroquinolines, such as pyrroloquinoline **106** (Scheme 16), from benzyl azide **107** and cyclic non-aromatic enamides [68]. A similar assemble of the corresponding indoloquinoline had previously been reported by the Zhai group [69]. The pyrroloquinoline **106** was obtained with a *cis* relationship between the two chiral centers, by reacting benzyl azide **107** with pyrrole **73f** in the presence of triflic acid in toluene. This synthesis, based on Aubè and co-workers’ discovery of acid, promoted benzyl azide to imine rearrangement [70], included two sequential Pictet–Spengler reactions upon in situ formation of an iminium intermediate **108** from benzyl azide **107**.

**Scheme 17:**
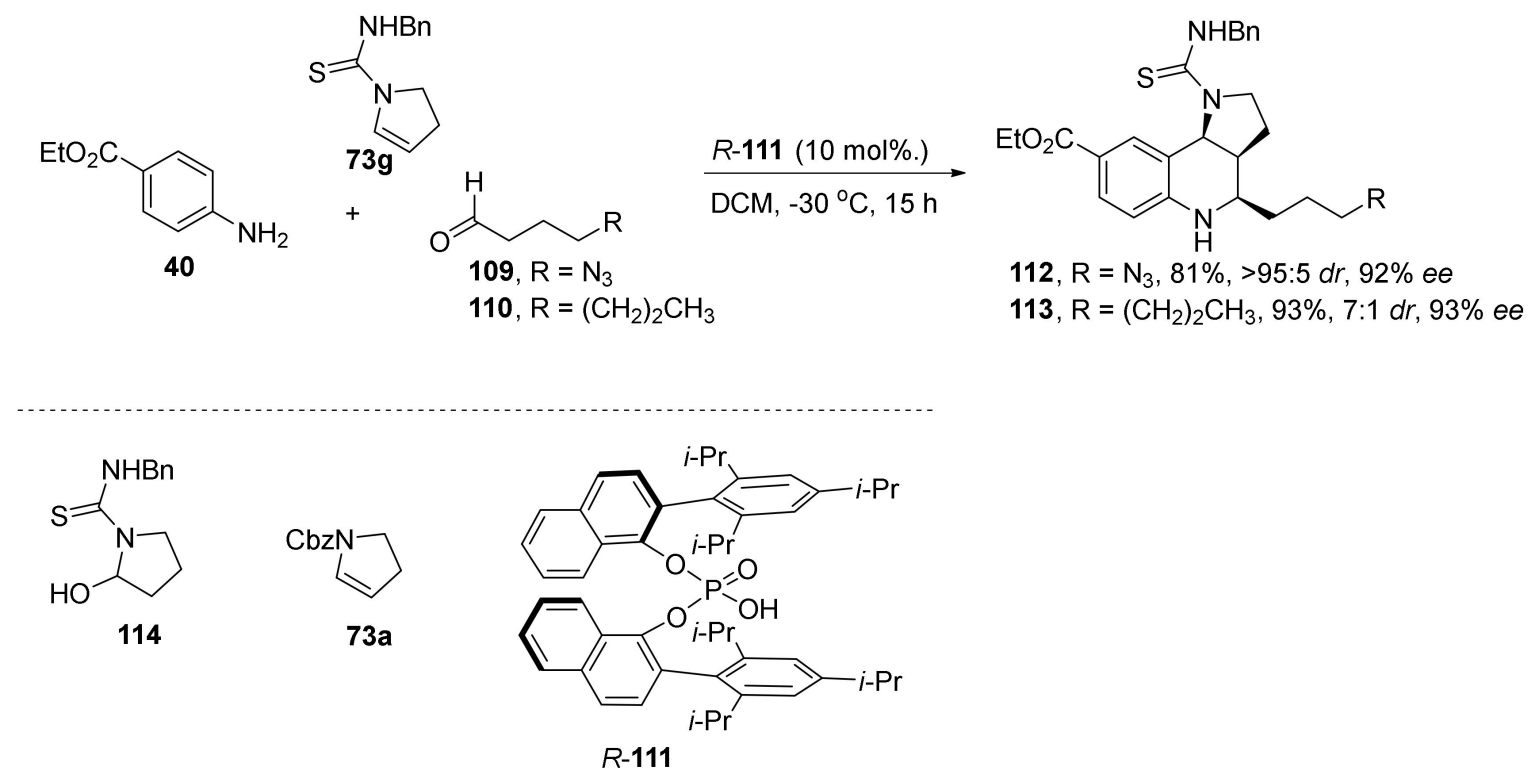
Scaffold synthesis by Masson and co-workers.

Masson and co-workers synthesized the endo-martinella scaffold in a chiral phosphoric acid-catalyzed enantioselective three-component Povarov reaction (Scheme 17) [71]. Aniline **40**, enamine **73g**, and aldehyde **109** or **110** were stirred together in the presence of phosphoric acid catalyst (*R*)-**111** to provide endo-martinella scaffolds **112** and **113** in high enantiomeric excess. A similar multicomponent reaction between aniline, benzaldehyde, and pyrrolidine **114**, catalyzed by lanthanide triflate to assemble the martinella scaffold was reported by Batey and Powell in 2001 [47]. Batey and Powell’s incentive for using the thiourea-protected pyrrolidine 114 was to provide the martinella scaffold with the guanidine precursor moiety pre-installed on the sterically hindered pyrrolidine nitrogen. In addition to this, Batey and Powell found that using thiourea **114** as the dienophile, instead of *N*-CBz enamide **73a**, favored formation of the *exo*-pyrroloquinoline scaffold.

In the phosphoric acid (*R*)-**111** catalyzed reaction, Masson and co-workers hypothesized that the benzyl-NH function of thiourea **73g** participated together with the in situ formed imine in hydrogen bonding interactions with the phosphoric acid (*R*)-**111**. This dual activation thereby enhanced enantioselectivity of this reaction to provide endo-stereoisomers **112** and **113** in high ee.

**Scheme 18:**
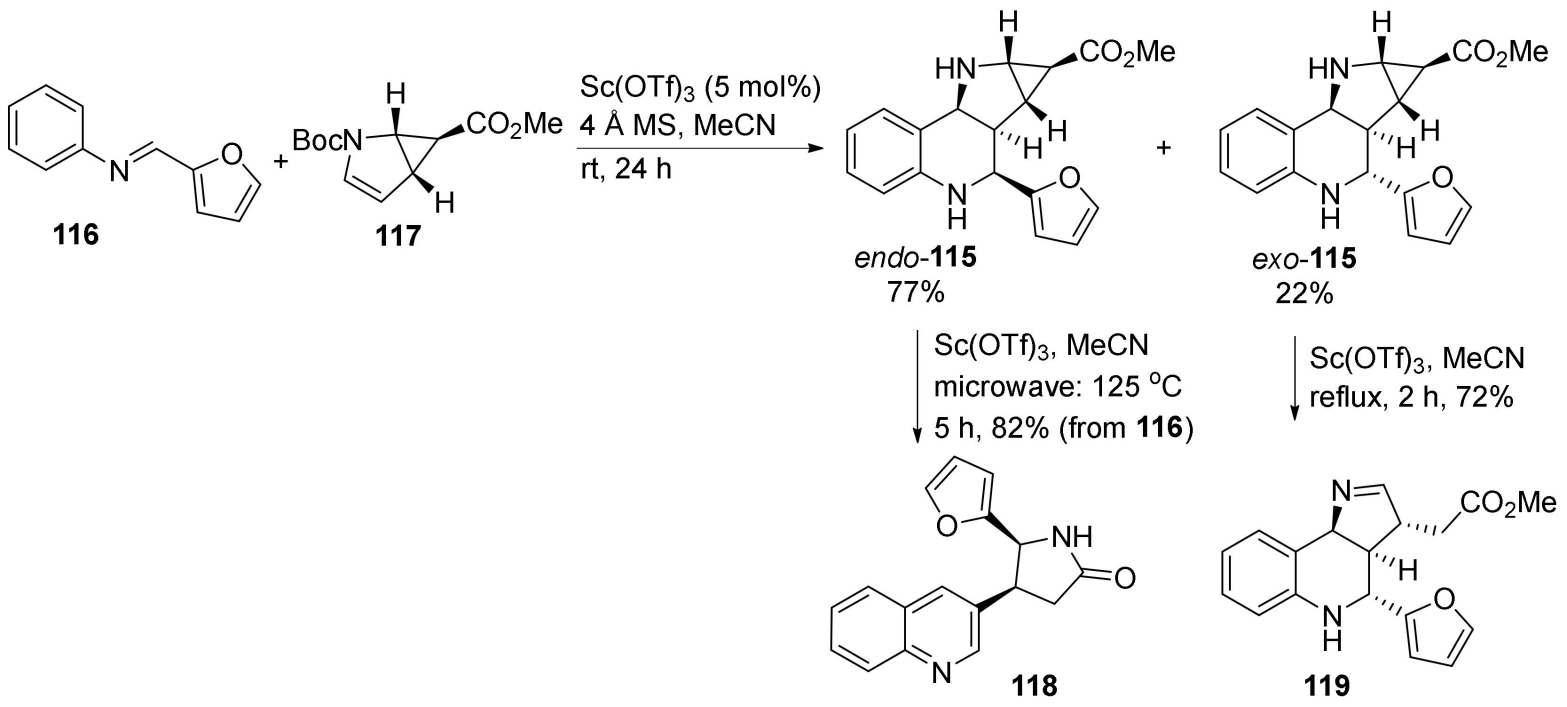
Assembly of the pyrroloquinoline scaffold by Roy and Reiser.

Roy and Reiser assembled the pyraoloquinoline scaffold **115** in a scandium triflate catalyzed Povarov reaction between aldimine **116** and enamide **117** at ambient temperature (Scheme 18) [72]. Upon applying heat to the reaction, they obtained 4,5-*cis*-disubstituted pyrrolidinone **118**, a lead structure for pharmaceutical compounds that target diseases such as osteoporosis [73], in 82% yield. They further proposed a reaction mechanism for the formation of the pyrrolidinone **118** that included assembly of the tetracycle **115** in a Povarov reaction**,** which, upon ring opening of the cyclopropane, provided an iminium compound (such as imine **119**). Further migration of the furan group, followed by aromatization of the quinoline structure and lactamization of the pyrrolidine moiety upon Boc-deprotection, could then provide pyrrolidinone **118**. In the proposed mechanism, the cis-relationship between substituents on the pyrrolidinone **118** arose from the Povarov product endo-**115**. This proposal was confirmed when heating of tetracycle exo-**115** in the presence of scandium triflate provided pyrroloquinoline **119**, and not the pyrrolidinone **118**.

**Scheme 19:**
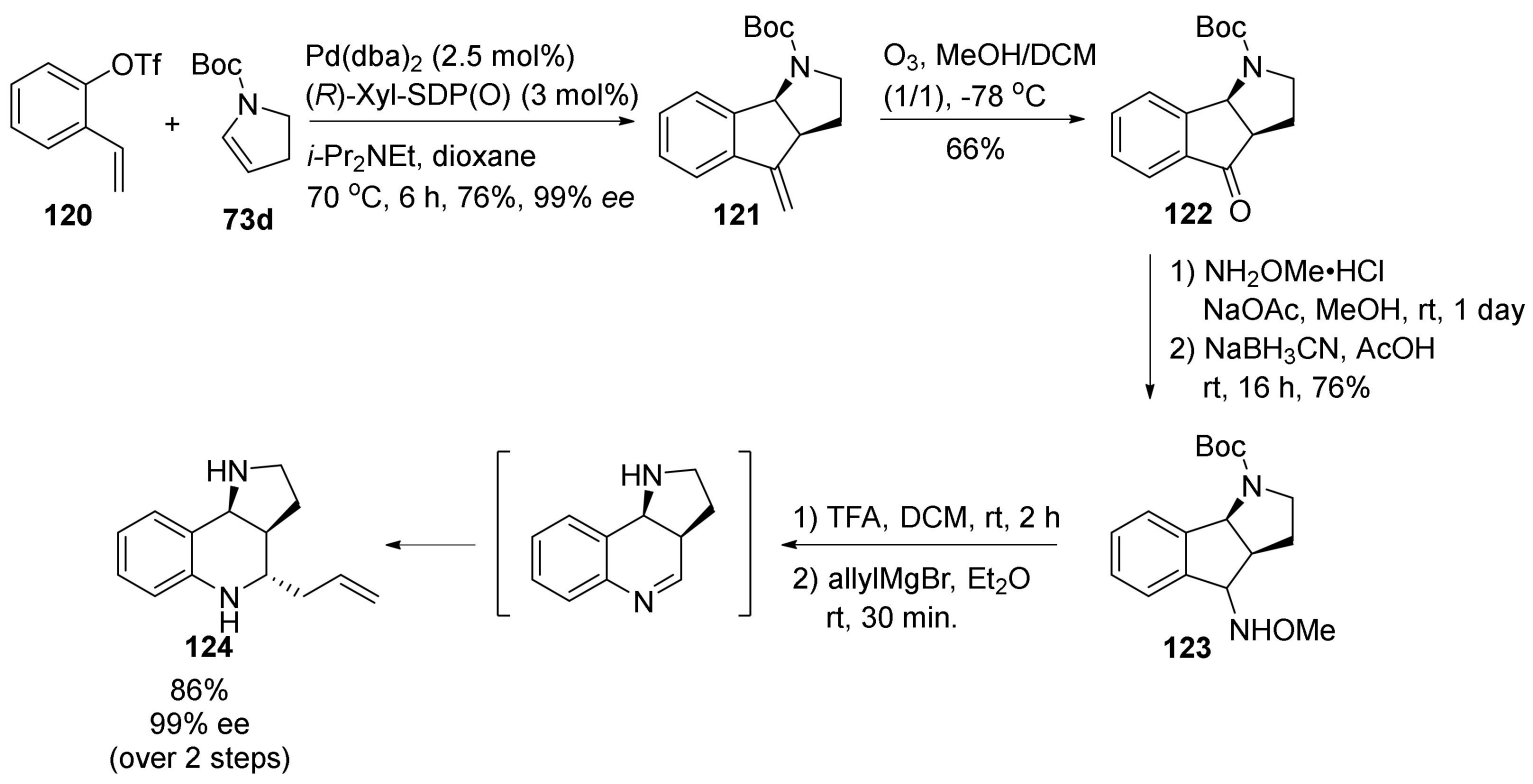
Scaffold synthesis by Zhou and co-workers.

Another asymmetric approach towards the pyrroloquinolines was presented by Zhou and co-workers in 2013 [74]. The stereochemistry was installed in a palladium catalyzed asymmetric intermolecular cyclization reaction between triflate **120** and Boc-protected pyrrolidine **73d** to obtain the tricyclic alkene **121** in 76% yield and excellent ee (Scheme 19). The following treatment with ozone afforded ketone **122**. Condensation of *O*-methyl hydroxylamine with the carbonyl moiety in ketone **122** followed by reduction of the resulting *O*-methyl oxime with sodium cyanoborohydride afforded amine **123**. With compound **123** in hand, the further plan was to use Miyata and co-workers’ strategy (Scheme 8) for ring expansion to finalize the tricyclic scaffold **124** [54]. However, only upon *N*-Boc deprotection did ring expansion occur under Miyata and co-workers’ reaction conditions. Thus, after removing the Boc group by treatment with trifluoroacetic acid (TFA), pyrroloquinoline **124** was obtained in 86% yield with retained enantioselectivity.

**Scheme 20:**
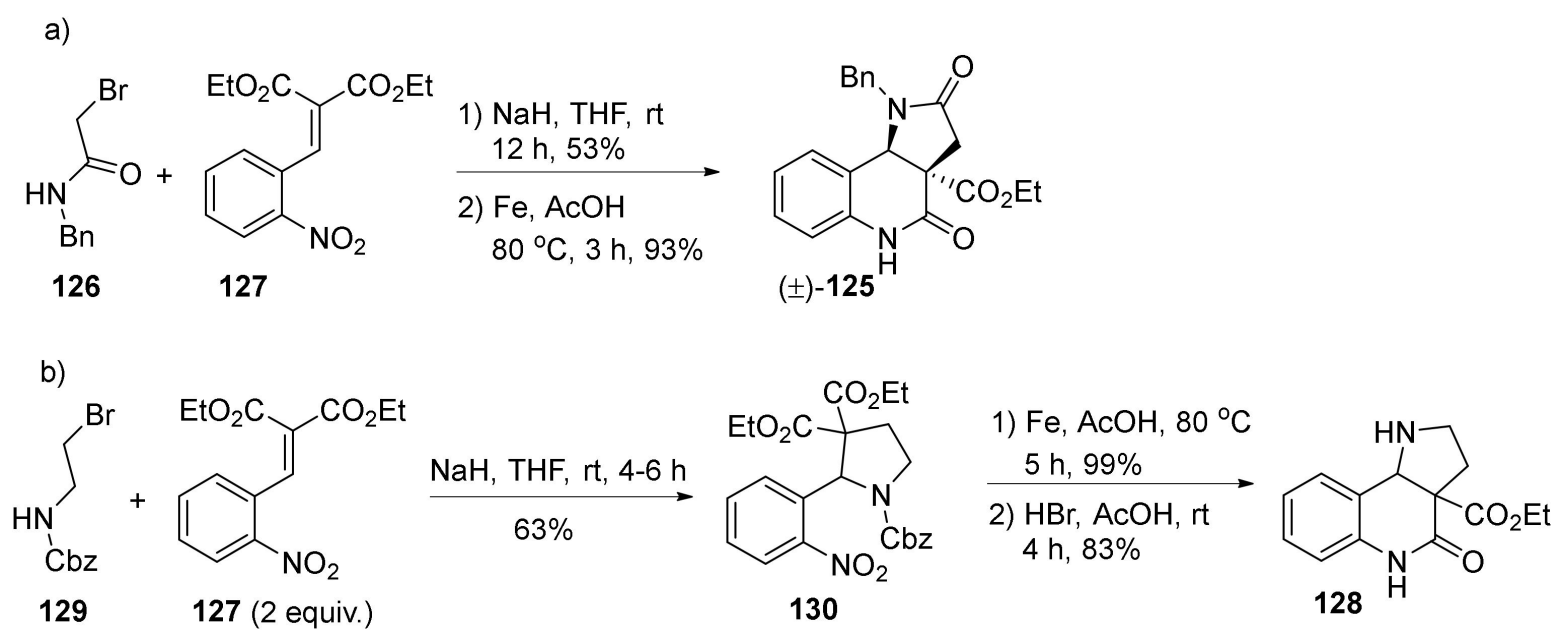
Scaffold synthesis by Daïch and co-workers: (**a**) 2008 approach; (**b**) 2013 approach.

In a previously reported strategy, included in the 2008 review by Lovely and Bararinarayana [34], Daïch and co-workers reported the synthesis of the pyrroloquinoline scaffold **125** from α-bromoacetamide **126** and Michael acceptor **127** (Scheme 20a) [75]. In 2013, Daïch and co-workers reported an extension of this method in the synthesis of pyrroloquinoline **128** from bromoethyl carbamate **129** and diethyl malonate **127** (Scheme 20b) [76]. The reaction between carbamate **129** and Michael acceptor **127** in the presence of sodium hydride provided pyrrolidine **130** through an aza-Michael/intramolecular substitution reaction tandem sequence. The group experienced that all attempts to hydrogenate pyrrolidine **130** failed. They thus obtained the martinella scaffold **128** by iron-catalyzed reduction of nitrobenzyl-pyrrolidine **130** followed by acidic deprotection of the *N*-Cbz group.

**Scheme 21:**
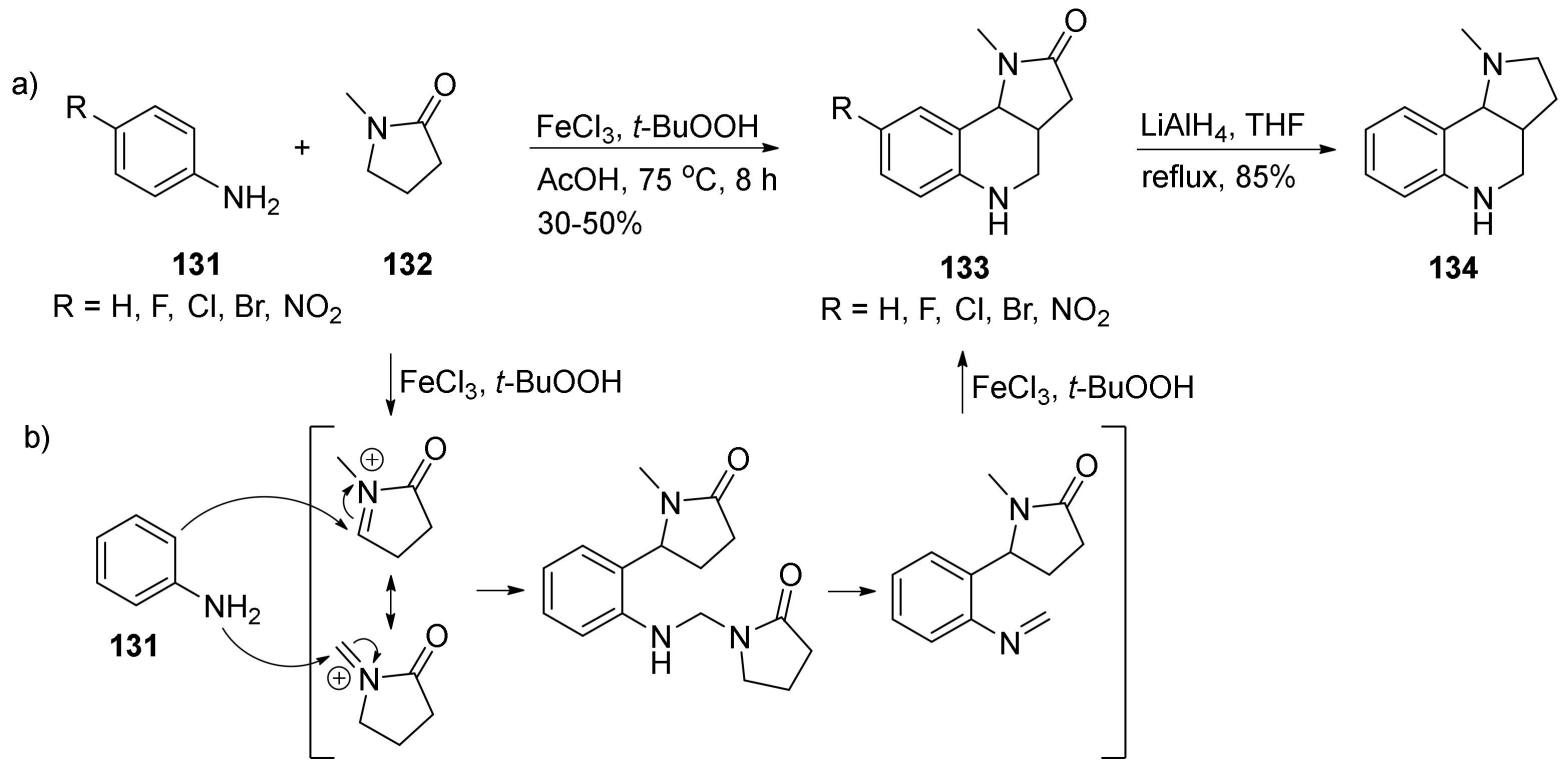
(**a**) Reaction conditions and (**b**) the proposed mechanism for scaffold assembly by Bao and co-workers.

Bao and co-workers applied oxidative conditions to aniline and electron deficient anilines **131** dissolved in lactam **132** to form the tricyclic scaffold **133** (Scheme 21a) [77]. By using iron trichloride (FeCl_3_) as catalyst and tert-butylperoxide (*t*-BuOOH) as oxidant in the presence of acetic acid (AcOH), the pyrroquinoline **133** was formed in 30–50% yield. Bao and co-workers further presented a plausible mechanism for the formation of the tricyclic scaffold **133** (Scheme 21b). The mechanism commenced with a single electron transfer (SET) oxidation of lactam **132** to the corresponding iminium ions. One C–C and one C–N bond was further formed upon addition of aniline **131**. Elimination of a 2-pyrrolidone followed by an SET oxidation provided the pyrroloquinoline **133**. Further reduction of the lactam moiety, using lithium aluminum hydride, afforded the hexahydropyrroloquinoline structure **134** in 85% yield.

**Scheme 22:**
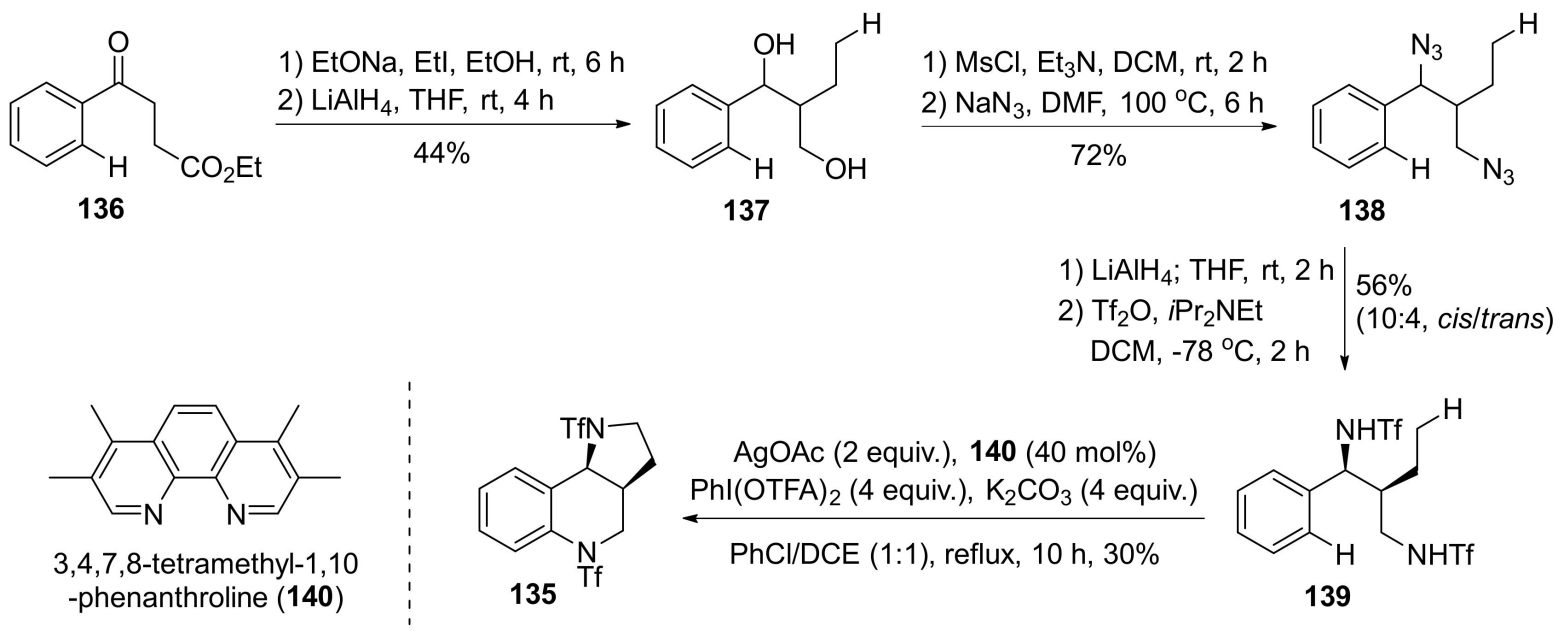
Scaffold synthesis by Zhang and co-workers.

Shi and co-workers obtained the tricyclic scaffold **135** from silver catalyzed amination of inert CH-bonds (Scheme 22) [78]. The synthesis commenced from ketone **136**, which was alkylated with ethyl iodide, followed by a lithium aluminum hydride (LiAlH_4_) reduction of the ketone and ester moieties. The resulting diol **137** was converted to the corresponding azide **138** via mesylation with mesyl chloride followed by treatment with sodium azide. Reduction of the diazide **138** with LiAlH_4_ followed by *N*-triflation wth triflic anhydride (Tf_2_O) afforded diamine **139** with a 10:4 *cis*/*trans* relationship between the benzylic amine and the adjacent ethyl chain. The purity of the *cis*-isomer *cis*-**139** was further enhanced to approximately 90% by crystallizing out its isomer *trans*-**139**. Pyrroloquinoline **135** was finally obtained from diamine **139** by a silver catalyzed amination reaction employing silver acetate (AgOAc) as the silver source, phenathroline **140** as the ligand, PhI(OTFA)_2_ as the oxidant, and potassium carbonate as the base in a 1:1 solvent mixture of chlorobenzene (PhCl) and dichloroethane (DCE). The reaction was run at reflux for 10 h to afford the tricyclic scaffold 135 in 30% yield.

**Scheme 23:**
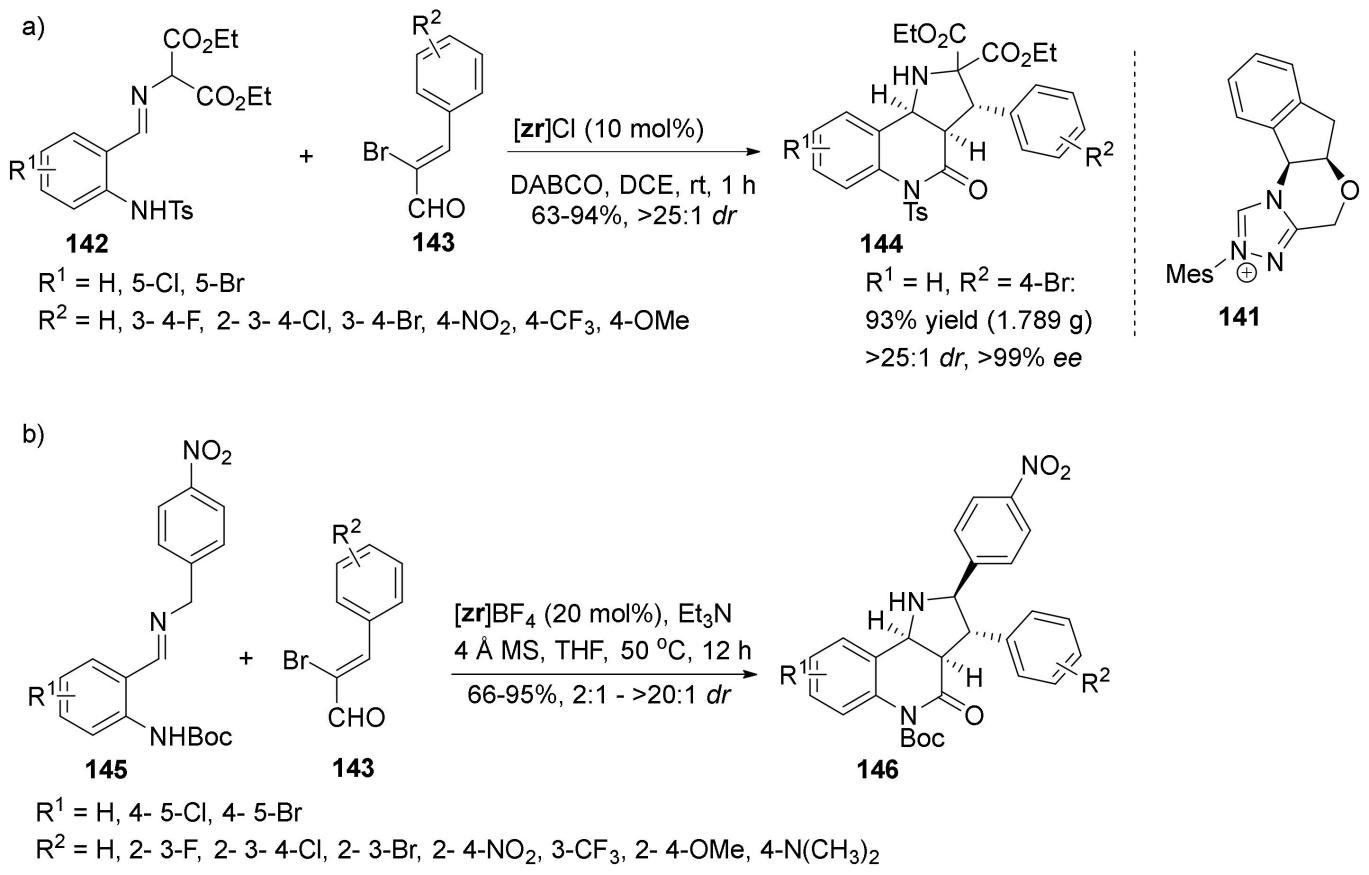
Scaffold synthesis by (**a**) Hui and co-workers and (**b**) Jin, Chi, and co-workers.

In 2014, Hui and co-workers employed a stereoselective *N*-heterocyclic carbene **141** catalyzed cascade reaction between ortho-aromatic aldimines (such as **142**) and 2-bromoenals (such as **143**) to form the martinella scaffold in high yield and excellent enantioselectivity (Scheme 23a) [79]. They hypothesized that this one-pot cascade reaction includes a Michael addition followed by a Mannich reaction and a lactamization to produce the pyrroloquinoline scaffold with the formation of three stereocenters in the sequence. In their optimization studies, the group found that superior stereoselectivity of the reaction was observed when using DABCO as the base rather than 1,8-diazabicyclic[5.4.0]undec-7-ene (DBU), triethyl amine, or cesium carbonate. They also experienced that the choice of solvent was crucial for the stereoselective outcome of the reaction, with 1,2-dichloroethane (DCE) favoring the reaction with highest diastereoselectivity. A scope study revealed the generality of the reaction. The applicability of the reaction was finally demonstrated with a gram scale synthesis of the bromo-phenyl pyrroloquinoline **144** in 93% yield and >25:1 *dr*. The carbene catalyzed reaction was also explored by Jin, Chi, and co-workers in 2018 (Scheme 23b) [80]. Instead of the diester imine **142** employed by Hui and co-workers, the *p*-nitro benzyl imine **145** was reacted with bromoenal **143** to form the pyrroloquinoline scaffold **146** in moderate to high yield and diastereomeric ratio.

**Scheme 24:**
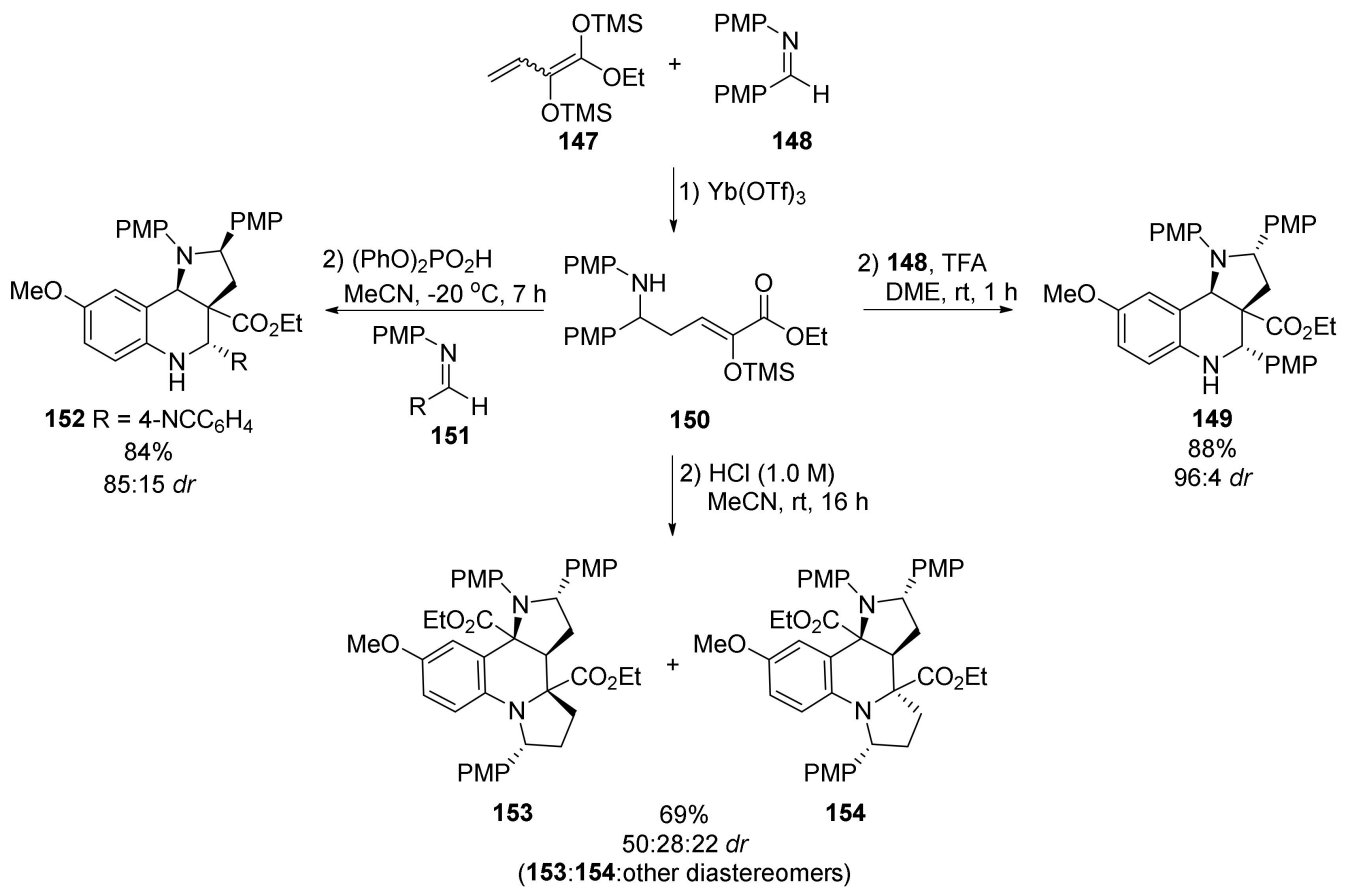
Synthesis of the pyrroloquinoline and the dipyrroloquinoline scaffolds by Schneider and co-workers.

Schneider and co-workers assembled the martinella scaffold in a sequential vinylogous Mannich–Mannich–Pictet–Spengler reaction [81]. Addition of bis-silyl dienediolate **147** to imine **148** in a vinologous Mannich reaction followed by a second addition of imine **148** provided pyrroloquinoline **149** in high yield and high diastereoselectivity (Scheme 24). In their proposed mechanism, the imine was added to silyl enol **150** in a Mannich reaction to form a diamino-α-keto ester. This reactive intermediate could then spontaneously cyclize to an imine followed by a Pictet–Spengler reaction with the neighbouring anisidine to form pyrroloquinoline **149**.

In a scope study, they observed that low diastereoselectivity was induced in the reactions between intermediate **150** and the electron poor imines, such as imine **151**. Through further work, they found that the diastereomeric outcome of the reaction could be flipped by altering reaction conditions. When acetonitrile was used as solvent, instead of 1,2-dimethoxyethane, and by altering the Brønsted acid from trifluoroacetic acid (TFA) to diphenyl phosphate, in addition to running the reaction at low temperature (−20 °C), they obtained diastereomer **152** with 85:15 dr in 84% yield.

The Schneider group reported a follow-up of this synthesis in 2018 [82]. When intermediate **150** was subjected to a Brønsted acid, such as HCl, dipyrroloquinolines **153** and **154** were formed in a combined yield of 69% and a ratio of 50:28:22 (**153**:**154**:other diastereomers) (Scheme 24). The group proposed that the cylic imine formed from intermediate **150** in an acid catalyzed reaction reacted with its enamine tautomer in a Mannich reaction to form a dimer. This was then followed by a Pictet–Spengler cyclization to form the dipyrroloquinoline scaffold **153** and **154**, with the major diastereomer **153** in endo-configuration.

**Scheme 25:**
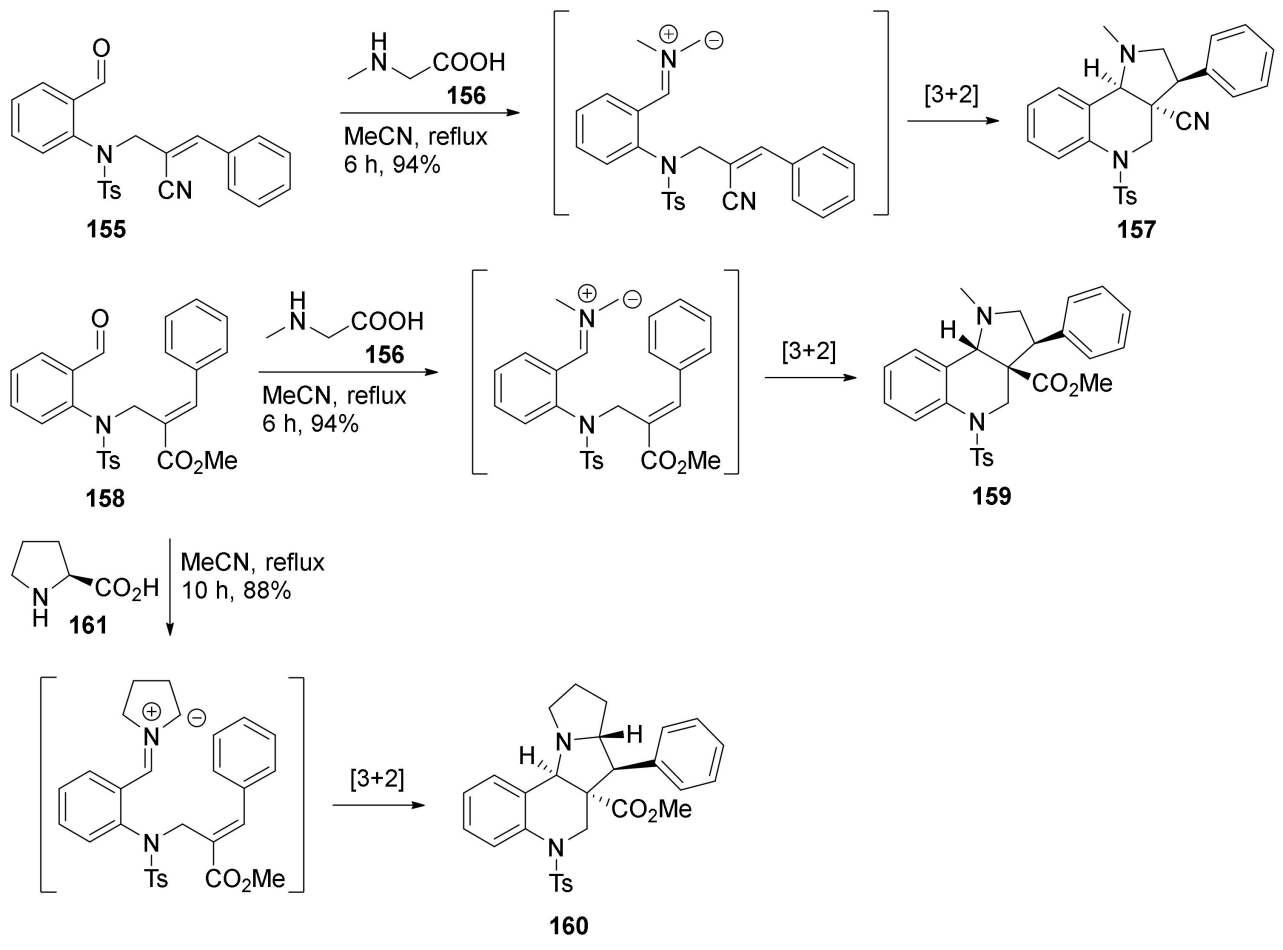
Scaffold synthesis by Bakthadoss and co-workers.

Bakthadoss and co-workers obtained the martinella scaffold from Bayliss Hillman derivatives via a 1,3-dipolar cycloaddition [83]. *N*-tosyl-*N*-allyl-2-aminobenzaldehyde (**155**) was treated with *N*-methyl glycine (**156**) to form pyrroloquinoline **157** in 94% yield through imine formation and decarboxylation followed by a 1,3-dipolar cyclization reaction (Scheme 25).

Pyrroloquinoline **157** comprised a trans-relationship between the nitrile and benzyl functionality. The reaction between *N*-allylated aldehyde **158** and glycine **156** provided pyrroloquinoline **159** with the benzyl moiety in cis-configuration to the methyl ester. The authors remark that the stereoselective outcomes coincide with the nature of the 1,3-dipolar addition reaction, with the *cis*- and *trans*-products **157** and **159** originating from *cis*-alkene **155** and *trans*-alkene **158**, respectively. The group further produced tetracyclic pyrrolizinoquinoline **160** by treating aldehyde **158** with L-proline (**161**), using the same conditions as previously described.

**Scheme 26:**
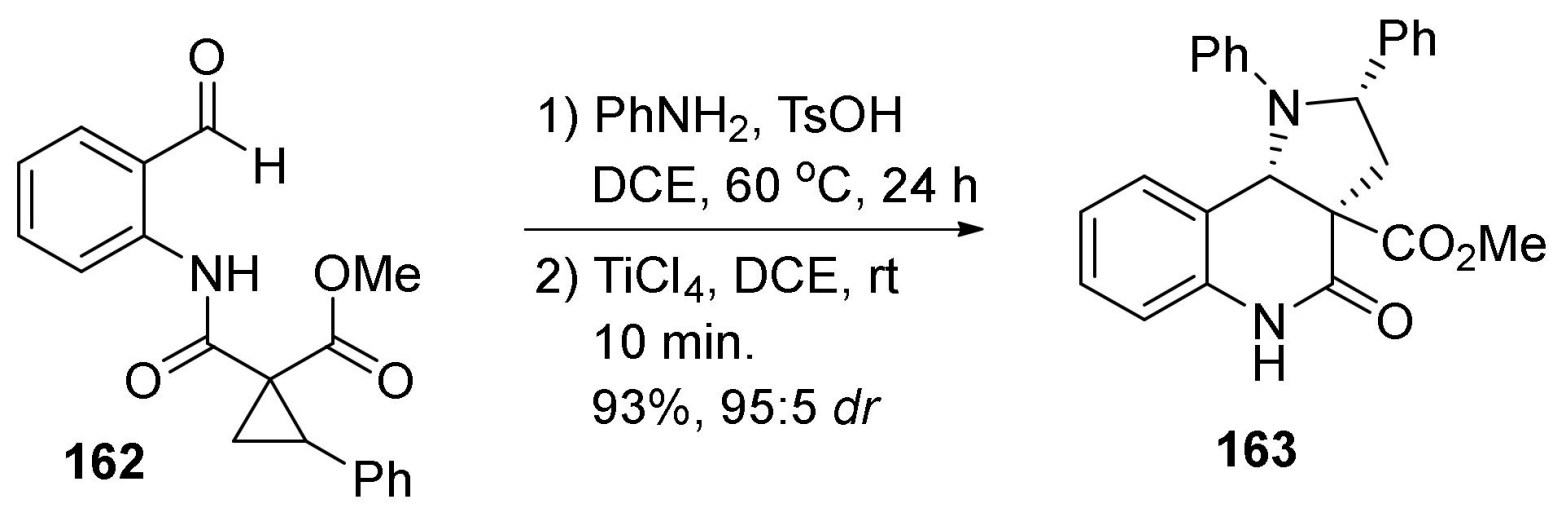
Scaffold synthesis by Xiang and co-workers.

Xiang and co-workers produced the martinella scaffold in an intramolecular [3+2] annulation of an acceptor-donor cyclopropane and an imine formed in situ [84]. Amide functionalized cyclopropane **162** was treated with aniline, initially at room temperature for 48 h, followed by addition of titanium tetrachloride (TiCl_4_) to provide pyrolloquinoline **163** in 40% yield and 8:2 dr (Scheme 26). The product **163** was obtained in 93% yield and 95:5 dr, and reaction time was decreased to 24 h when the reaction temperature was increased to 60 °C and using toluene sulfonic acid (TsOH) as an additive in the first reaction in the sequence.

**Scheme 27:**
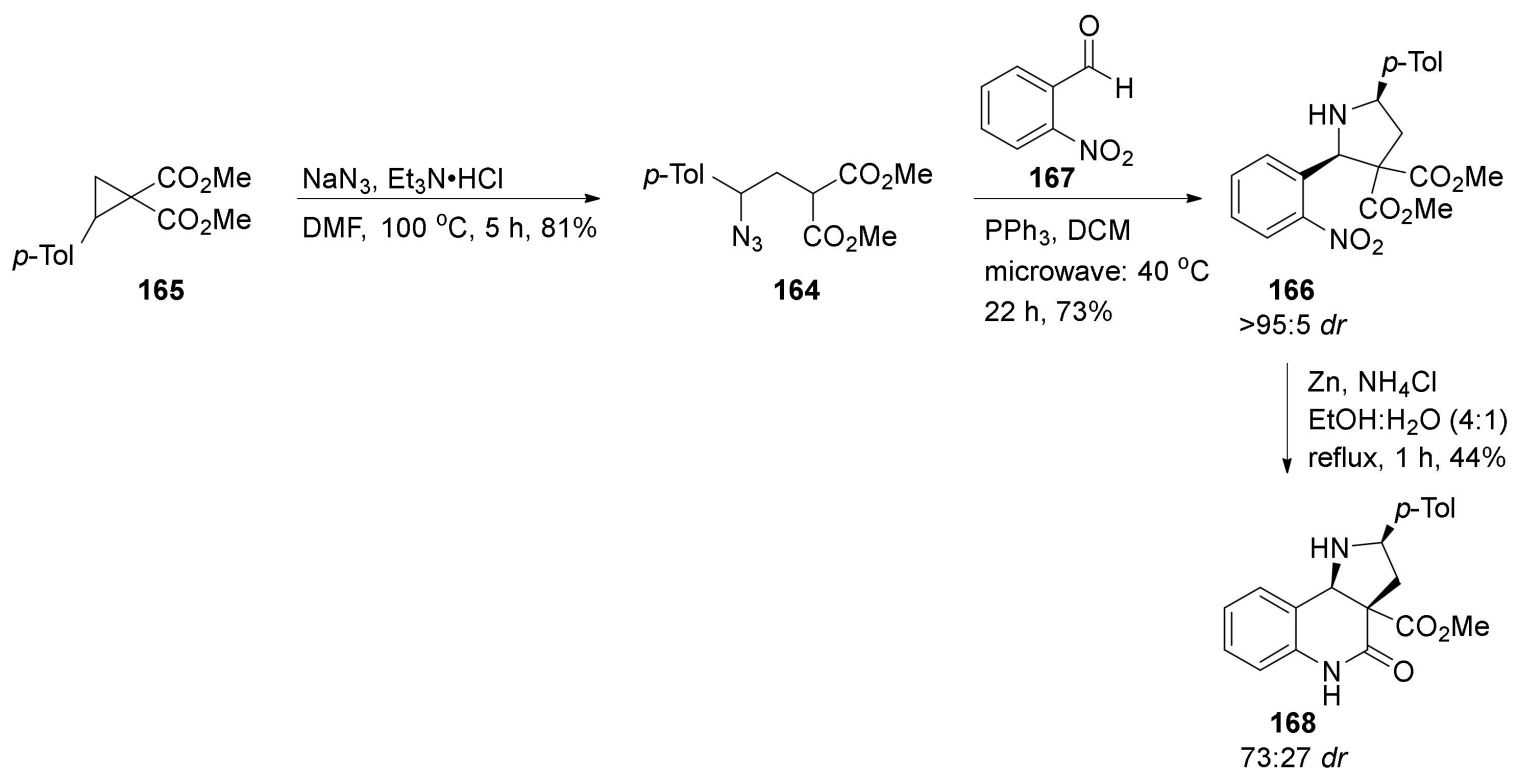
Scaffold synthesis by Trushkov and co-workers.

A similar version of the pyrroloquinoline scaffold **163** presented by Xiang and co-workers was also formed by Trushkov and co-workers as part of their combinatorial synthesis of di- and tetrahydropyrrole scaffolds (Scheme 27) [85]. The formation of the pyrroloquinoline scaffold commenced from azide **164**, a building block formed from cyclopropane **165** by a ring opening procedure developed in the Trushkov group [86]. The pyrroline **166** was further formed from azide **164** in a domino reaction including a Staudinger reduction of the azide functionality in compound **164**, followed by condensation with *p*-nitro benzaldehyde **167** and finally a reduction of the resulting iminium intermediate to the corresponding amine. Zink mediated nitro reduction of the domino reaction product 166 facilitated the amidative ring closure and thus the formation of pyrroloquinoline **168**.

**Scheme 28:**
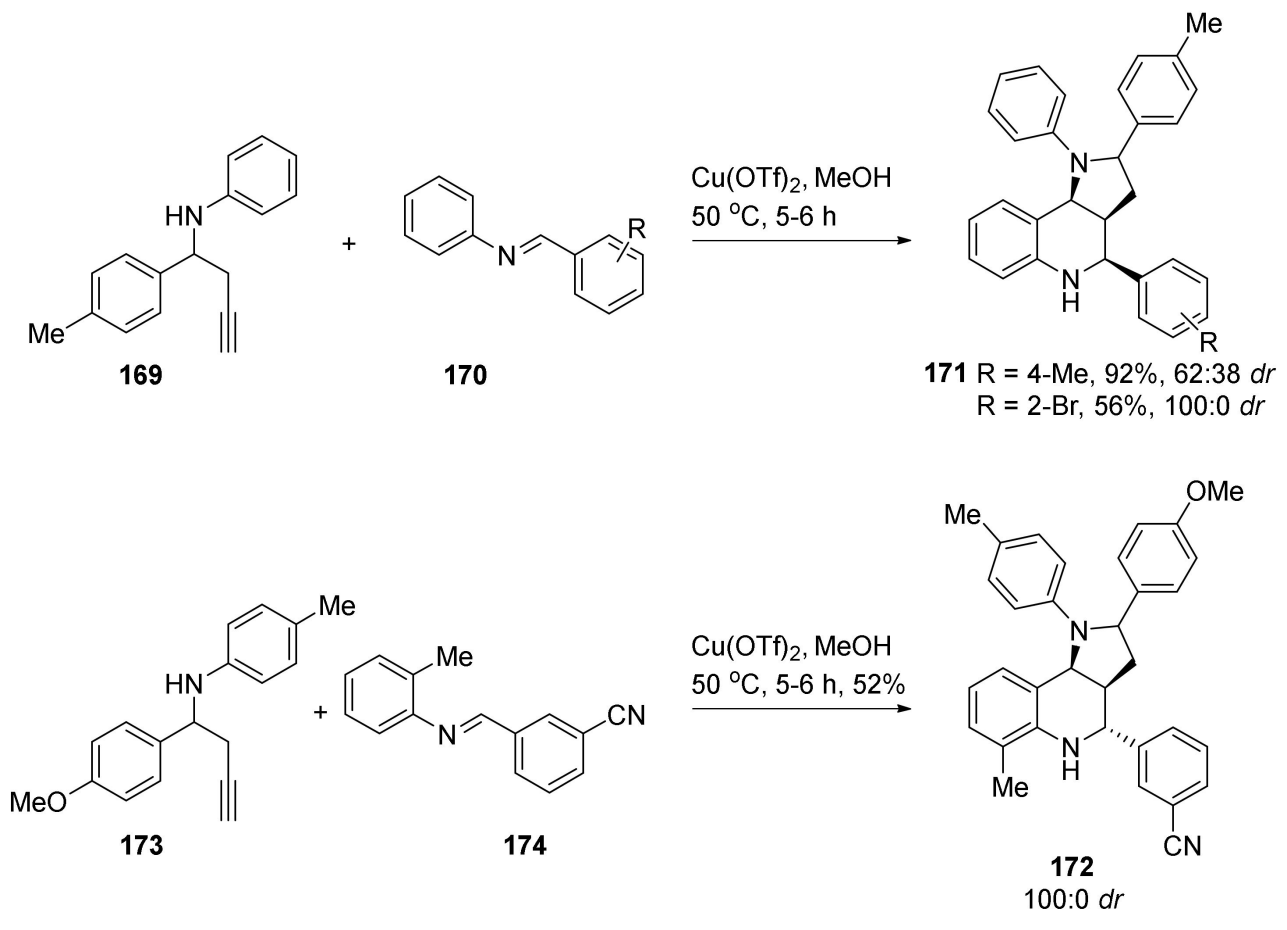
Pyrroloquinoline scaffold synthesis by Li and co-workers.

As part of a study on β-amino alkyne reactivity, Li and co-workers produced the martinella scaffold in a copper catalyzed cascade reaction including hydroamination of a β-amino alkyne followed by a Povarov reaction (Scheme 28) [87]. The β-amino alkyne **169** was activated with copper triflate to form the corresponding cyclic enamine, which in a Povarov reaction with the aromatic imine **170**, formed pyrroloquinoline **171**, with the major diastereomer in endo-configuration. The group further examined the reaction outcome from replacing copper triflate with other catalysts. Comparable results were obtained with copper chloride and iron triflate (Fe(OTf)_3_); however, using zinc triflate or mercury triflate did not provide the desired product. The protic catalysts triflic acid and benzoic acid had no effect on the reaction. In work aimed at broadening the scope of the reaction the group observed that different R-groups on the aromatic rings facilitated variations in the endo/exo preference of the reaction products, exemplified here with the two diastereoselective reactions that generated the endo-**171** and exo-**172** products, respectively.

**Scheme 29:**
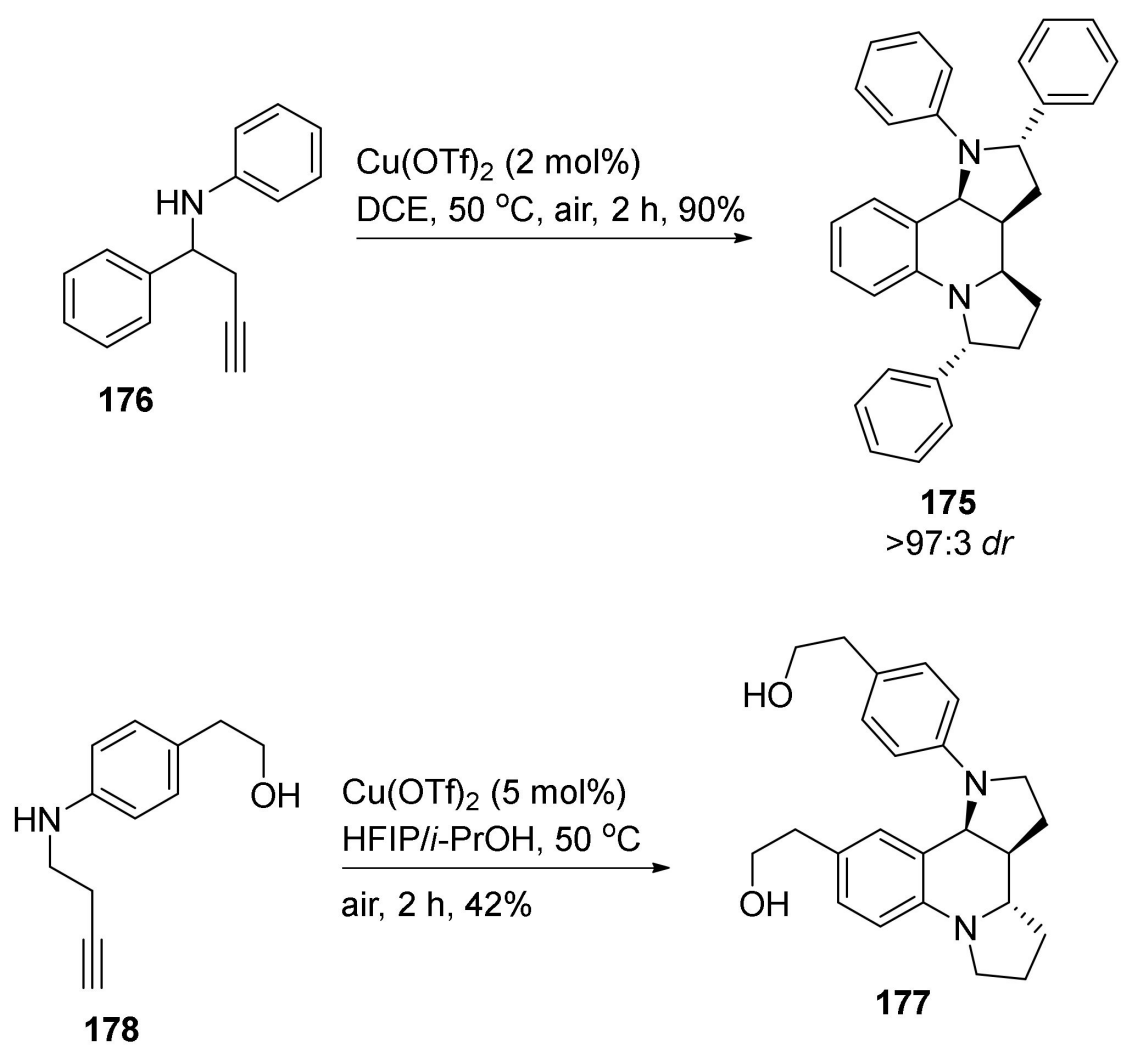
Dipyrroloquinoline scaffold synthesis by Li and co-workers.

Li and co-workers further extended the utility of the copper catalyzed reaction to include synthesis of dipyrroloquinolines (such as **175**) from aminoalkynes (such as **176**) (Scheme 29) [88]. Instead of using an aromatic imine **170** in a Povarov reaction, the dipyrroloquinoline **175** was formed as a dimer. The proposed mechanism for this dimerization was a formal [4+2]-cycloaddition of the enamine formed in situ from the copper catalyzed hydroamination of aminoalkyne **176**, to its imine tautomer. The group also showed that this method could be used to synthesize the aglycon moiety of Incargranine B (**177**) from aminoalkyne **178**.

**Scheme 30:**
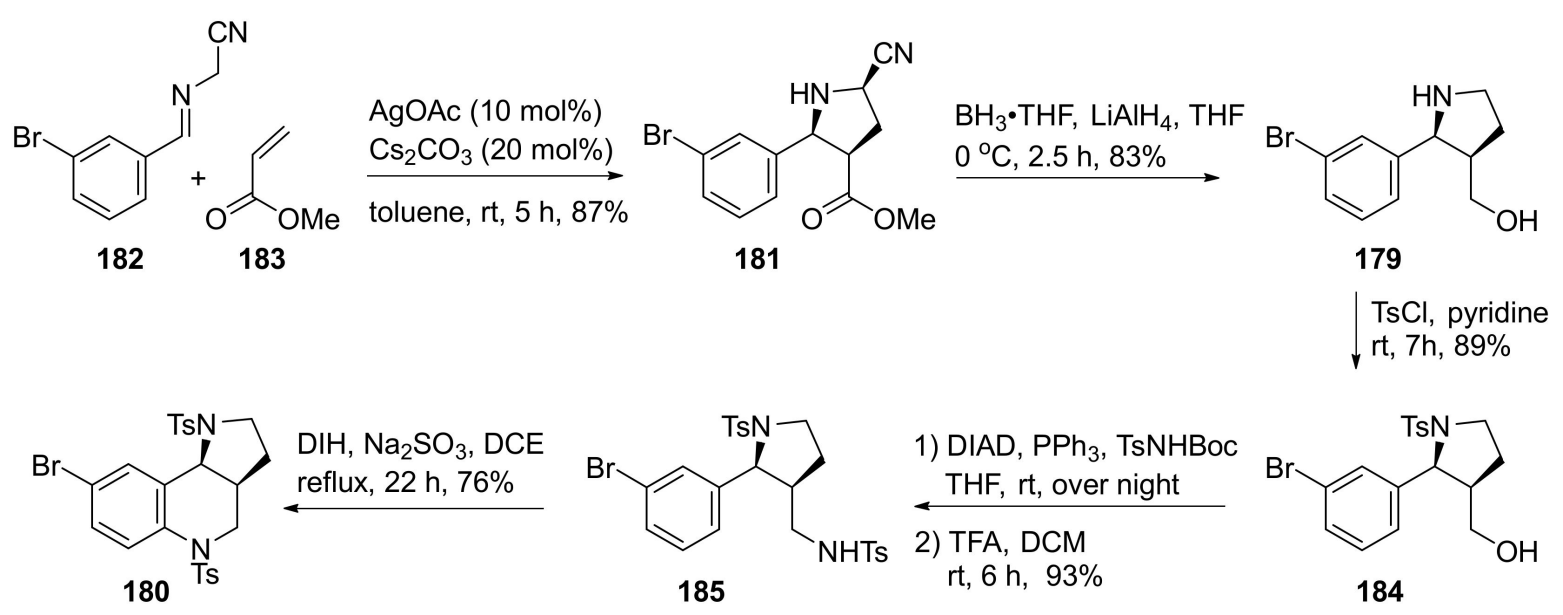
Scaffold synthesis by Zhang and co-workers.

In a scope study of their synthetic route to multisubstituted β-prolinols such as **179**, the Zhang group put together the tricyclic scaffold **180** (Scheme 30) [89]. A [3+2] cycloaddition mediated by silver acetate provided cyanoester **181** from imine 182 and Michael acceptor **183**. Cyanoester **181** was then converted to prolinol **179** via ester reduction and concomitant reductive decyanation. For the reduction reaction, Zhang and co-workers explored a variety of reducing agents, including lithium aluminum hydride (LiAlH_4_), diisobutylaluminium hydride (DIBAL-H), borohydride (BH_3_), sodium borohydride (NaBH_4_), and combinations of these. A combination of lithium aluminum hydride and borane provided prolinol **179** in highest yield. The *N*-tosylated prolinol **184** was obtained upon treatment with tosyl chloride, and further converted to the *N*-tosylated diamine **185** in a substitution reaction with *N*-Boc-toluenesulfonamide. A final aromatic C–H amination facilitated by 1,3-diiodo-5,5-dimethylhydantoin (DIH) furnished pyrroloquinoline **180** in 76% yield.

**Scheme 31:**
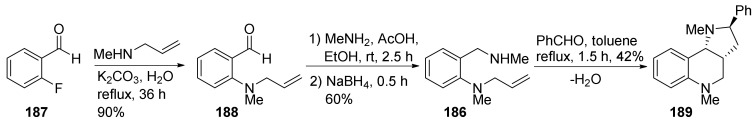
Scaffold synthesis by Xiang and co-workers.

Xiang and co-workers formed the tricyclic scaffold from azomethine ylides (Scheme 31) [90]. Ylide **186** was prepared by coupling of 2-fluotobenzaldehyde **187** with *N*-methyl allylamine followed by condensation of the resulting aldehyde **188** with methylamine. The pyrroloquinoline **189** was formed as a mixture of enantiomers in moderate yield in a 1,3-dipolar cycloaddition between azomethine ylide **186** and benzaldehyde, while removing water by azeotropic distillation. Xiang and co-workers further observed that the yield from the cycloaddition reaction was somewhat enhanced (53–56%) when the aromatic ring in aniline **186** contained electron withdrawing substituents, such as a chloro or nitro group. Conversely, they observed a decreased reaction yield (19%) when applying said reaction conditions to the *m*-methoxy derivative of aniline **186**.

**Scheme 32:**
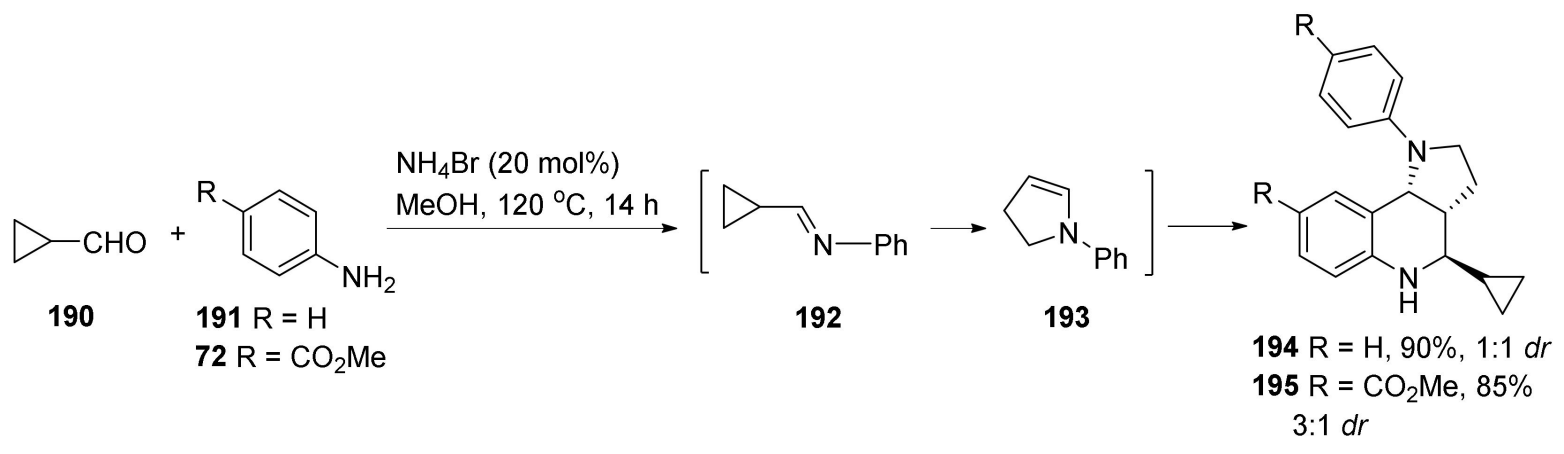
Scaffold synthesis by Huang and co-workers.

Another method for preparation of the tricyclic scaffold was presented by Huang and co-workers [91]. They treated cyclopropyl amine **190** and aniline **191** with ammonium bromide to promote formation of imine **192** in a condensation reaction (Scheme 32). Further catalytic isomerization of the cyclopropylimine **192** to dihydropyrrolidine **193** followed by a Povarov reaction between the imine **192** and the enamine **193** afforded pyrroloquinoline **194**. When conducting the reaction with halide-substituted anilines, the group found that the *p*-anilines provided the pyrroloquinolines in highest yield. Nearly all reactions provided the product in approximately 1:1 diastereomeric mixtures, except in the case of *p*-CO_2_Me-aniline **72**, which afforded the product **195** with the major stereoisomer in exo-configuration.

**Scheme 33:**
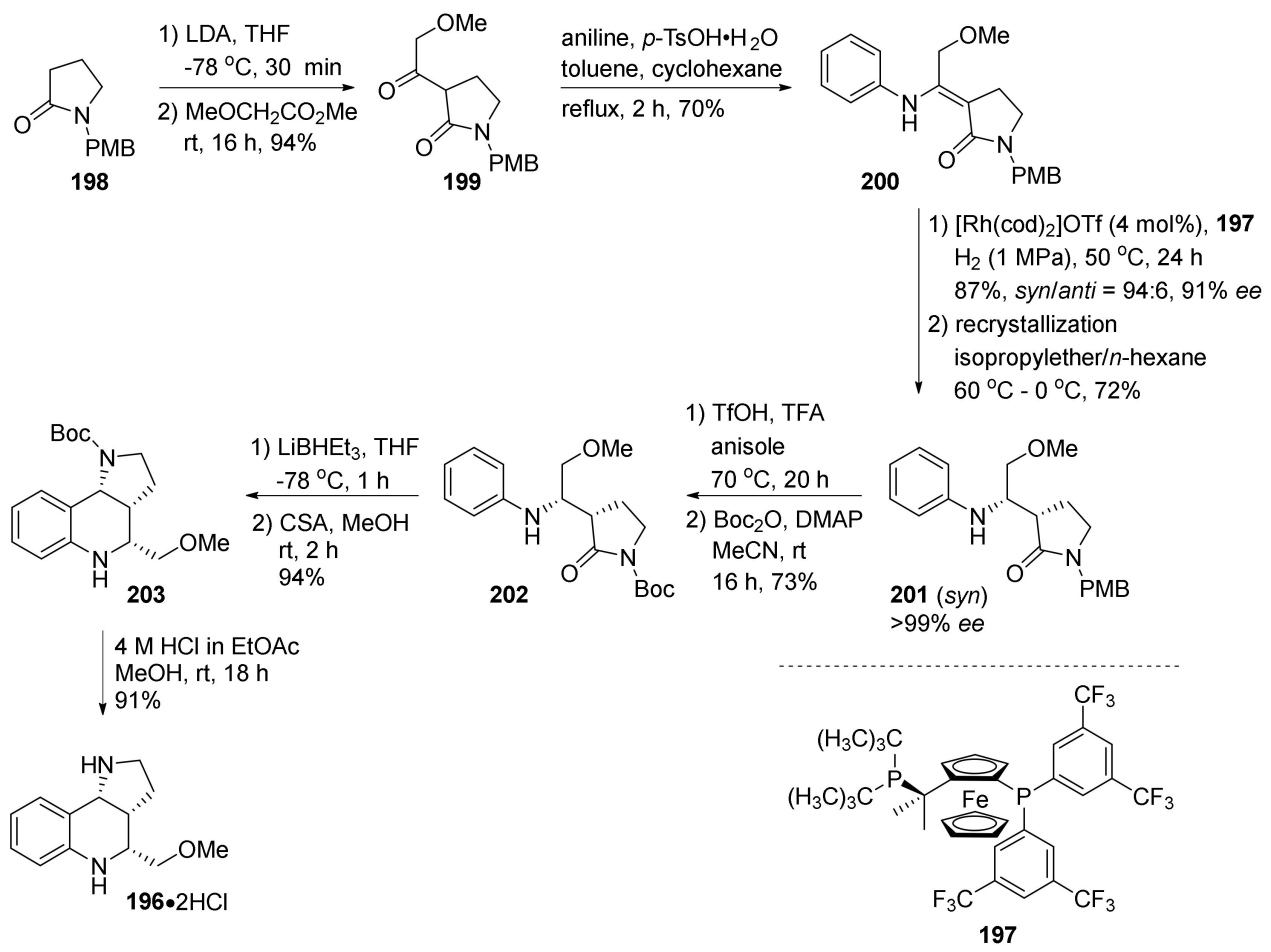
First generation rhodium catalyzed asymmetric synthesis of the pyrroloquinoline scaffold by Tatsuta and co-workers at Waseda University together with Kondo at Takeda Pharmaceutical company.

Tatsuta and co-workers at Waseda University together with Kondo at Takeda Pharmaceutical company developed a method for the synthesis of pyrroloquinoline **196** via an asymmetric hydrogenation reaction using a rhodium catalyst together with the ferro-phosphine ligand **197** (Scheme 33) [92,93]. The synthesis commenced from PMB-protected pyrrolidone **198**, which was alkylated with methyl methoxyacetate to form methoxy ketone **199**. Enamine **200** was obtained from the acid catalyzed condensation of ketone **199** with aniline. Rhodium-catalyzed asymmetric reduction of the enamine **200** followed by recrystallization in isopropylether and *n*-hexane afforded the enantiopure amine **201**. The *N*-protecting group was then converted from *p*-methoxybenzyl (PMB) to *t*-butyloxycarbonyl (Boc) to form compound **202**, which was compatible with the following amide reduction by lithium triethyl borohydride (LiBHEt_3_). Acid catalyzed intramolecular cyclization of the reduced product afforded pyrroloquinoline **203** in 94% yield over two steps. The final enantiopure product **196** was obtained in 21% overall yield as the HCl-salt upon acidic Boc-deprotection of carbamate **203**.

**Scheme 34:**
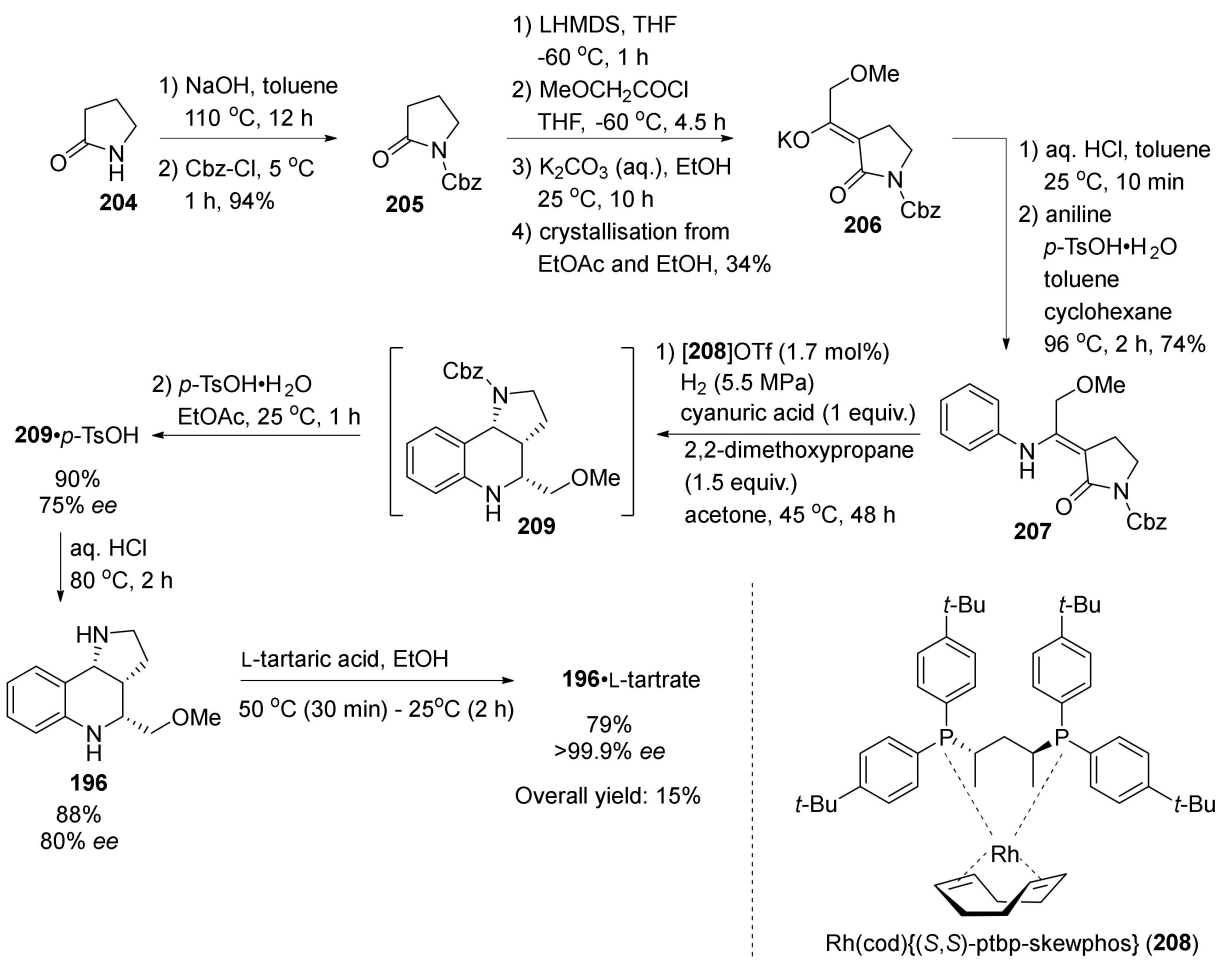
Second generation rhodium catalyzed asymmetric synthesis of the pyrroloquinoline scaffold by Yamada and co-workers at Takeda Pharmaceutical company.

A second generation asymmetric synthesis of the pyrroloquinoline methyl ether **196** (Scheme 33) was reported by Yamada and co-workers at Takeda Pharmaceutical company (Scheme 34) [94]. To avoid the need for changing the protecting group during the synthetic pathway, pyrrolidone **204** was protected with a benzyloxy carbonyl (Cbz) group. The resulting *N*-protected pyrrolidone **205** was acylated with methoxyacetyl chloride and converted to the more stable potassium salt **206** by treatment with potassium carbonate in ethanol followed by a recrystallization step. The salt **206** was liberated to the corresponding ketone by treatment with hydrochloric acid and then condensed with aniline to afford enamine **207** in 74% yield. Asymmetric hydrogenation of enamine **207** with rhodium catalyst **208** in the presence of cyanuric acid and 2,2-dimethoxypropane provided pyrroloquinoline **209**, which was converted into **209**•*p*-TsOH by treatment with *p*-toluenesulfonic acid. The direct conversion of enamine **207** to the cyclized product **209** under said reduction conditions was a fortunate surprise for Yamada and co-workers. They hypothesized that the cyclization process proceeded in a four-step domino cascade. In their proposed mechanism, asymmetric hydrogenation of enamine **207** followed by a carbonyl reduction of the pyrrolidone formed the corresponding *N*,*O*-acetal, which was dehydrated and sulfonated with triflate present in the mixture. Finally, the triflate participated in a cis-selective intramolecular cyclization reaction with the aniline moiety to assemble the pyrroloquinoline scaffold **209**. Amine **196** was obtained in 88% yield and 80% ee upon removal of the *N*-Cbz protecting group. Further chiral resolution with L-tartaric acid afforded the enantiopure tartrate salt **196**•L-tartrate. The reaction which was conducted in large scale gave 62 kg of the final product **196**•L-tartrate.

**Scheme 35:**
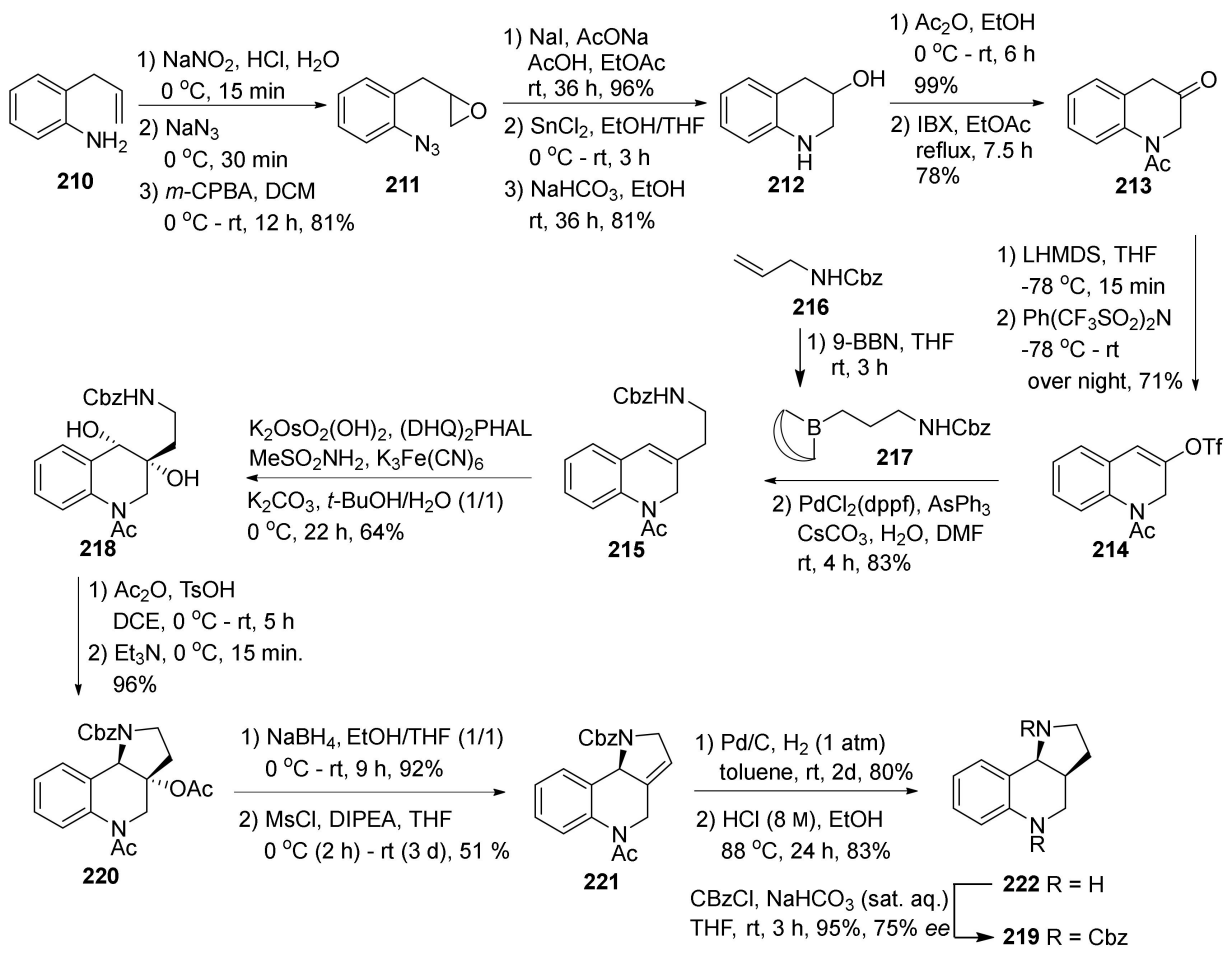
Scaffold synthesis by Lindbäck and Sydnes.

In our group, we investigated the applicability of Sharpless’ asymmetric strategies to obtain the tricyclic scaffold stereoselectively without the use of chiral building blocks or chiral auxiliary (Scheme 35) [95]. Allylamine **210** was converted to cyano-epoxide **211** by treatment with sodium azide followed by *m*-chloroperbenzoic acid (*m*-CPBA) epoxidation. Ring-opening of the epoxide **211** with sodium iodide followed by azide reduction and resulting quinoline cyclization afforded quinoline-3-ol **212**. The quinoline amine **212** was then acetylated and the alcohol functionality was oxidized with 2-iodoxybenzoic acid. By treating the resulting ketone **213** with triflating agent *N*-phenyl-bis(trifluoromethanesulfonimide), using lithium bis(trimethylsilyl)amide as the base, gave triflate **214** in 71% yield. The Sharpless dihydroxylation substrate, namely alkene **215**, was obtained from a sequential reaction entailing the hydroboration of *N*-Cbz-protected allylamine **216** followed by a Suzuki–Miyaura cross-coupling of the resulting boron **217** with triflate **214**. The alkene **215** was further subjected to Sharpless asymmetric dihydroxylation (AD) conditions, using (DHQ)_2_PHAL as the chiral ligand, to enantioselectively afford diol **218** in 64% yield.

The stereochemistry of the diol **218** was assigned by using Sharpless mneumonic device, which is a tool that can rationalize face selectivity of the AD reaction [96]. The stereochemical outcome from the AD reaction was later confirmed by HPLC analysis of *N*-Cbz protected pyrroloquinoline **219** on a chiral column. Acid catalyzed acetylation of diol **218** facilitated formation of the tricyclic structure **220** via an intramolecular substitution reaction. Based on the mechanism proposed by Jagadeessh and Rao [97], we hypothesized that the diacetate first underwent an intramolecular S_N_1 reaction, forming an acetoxonium ion intermediate. The cyclized cis-product **220** was subsequently generated from a nucleophilic attack on the acetoxonium ion by the Cbz-protected amine. Ester **220** was reduced with sodium borohydride to the corresponding alcohol and then dehydrated to alkene **221** by treatment with MsCl and Hunig’s base (DIPEA). Hydrogenation of the alkene **221** followed by acid promoted removal of the *N*-protecting groups, afforded the final product **222**.

**Scheme 36:**
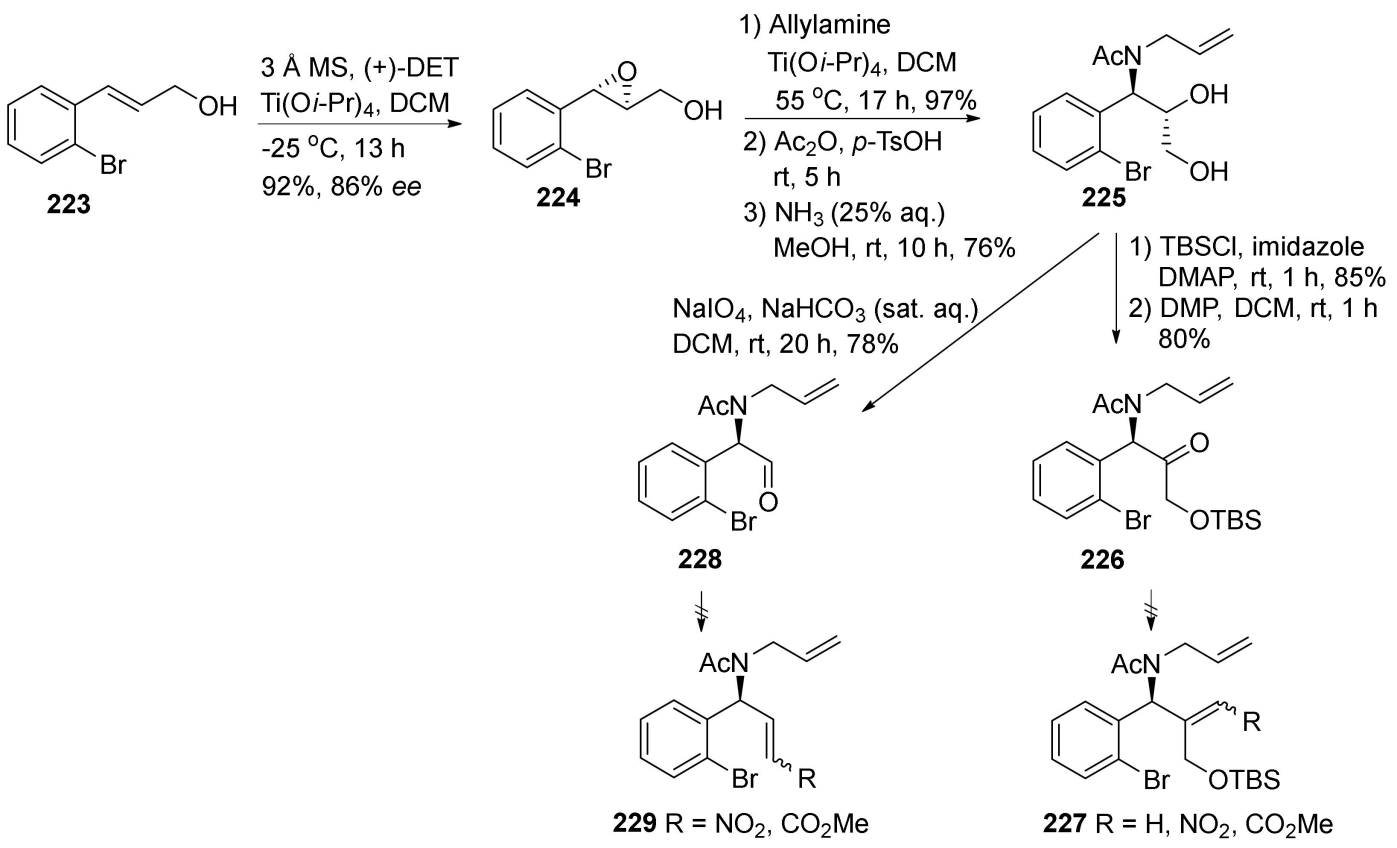
Synthetic approach towards the pyrroloquinoline scaffold by Haarr and Sydnes.

After the first generation of scaffold synthesis, an alternative synthetic strategy to enhance enantioselectivity of the pyrroloquinoline scaffold and at the same time install handles on the scaffold for further elaboration to martinellic acid was initiated by Haarr and Sydnes (Scheme 36) [98]. Cinnamyl alcohol **223** was subjected to the Sharpless epoxidation reaction to afford bromo-epoxide **224** in 86% ee. Epoxide **224** was then regioselectively ring opened with allylamine in the presence of titanium(IV)isopropoxide to afford the corresponding amino-diol, which was subjected to a protection–deprotection sequence to afford the *N*-acetylated diol **225**. In our synthetic design, we envisioned using the two alcohol moieties to first perform ring closing of the pyrrolidine ring followed by ring closing of the hetero-quinoline ring. Attempts to selectively oxidize the primary or the secondary alcohol moieties was unsuccessful. Selective silyl protection of the primary alcohol was thus conducted, followed by oxidation of the secondary alcohol using Dess Martin Periodinane (DMP) to form ketone **226**. In order to accomplish cyclization of the pyrrolidine ring via a ring closing metathesis with the *N*-allyl functionality, we attempted to convert ketone **226** to alkene **227**. No reaction took place under Wittig or Lombardo reaction conditions. Attempts to form the corresponding nitroalkene by reacting ketone **226** with nitromethane followed by a mesylation–elimination sequence was also unsuccessful. We speculated that there could be steric issues that caused the failure to install the alkene. Diol **225** was thus subjected to sodium periodate mediated oxidative cleavage to obtain aldehyde **228**. Attempts of alkene formation were again unsuccessful and no conversion of aldehyde **228** to its corresponding alkene **229** was observed. Other efforts to work around the encountered synthetic challenges were conducted; however, those efforts were in vain.

## 4. Synthesis of Dipyrroloquinolines Towards Natural Products Seneciobipyrrolidine (3) and Incargranine B (4)

**Figure 2:**
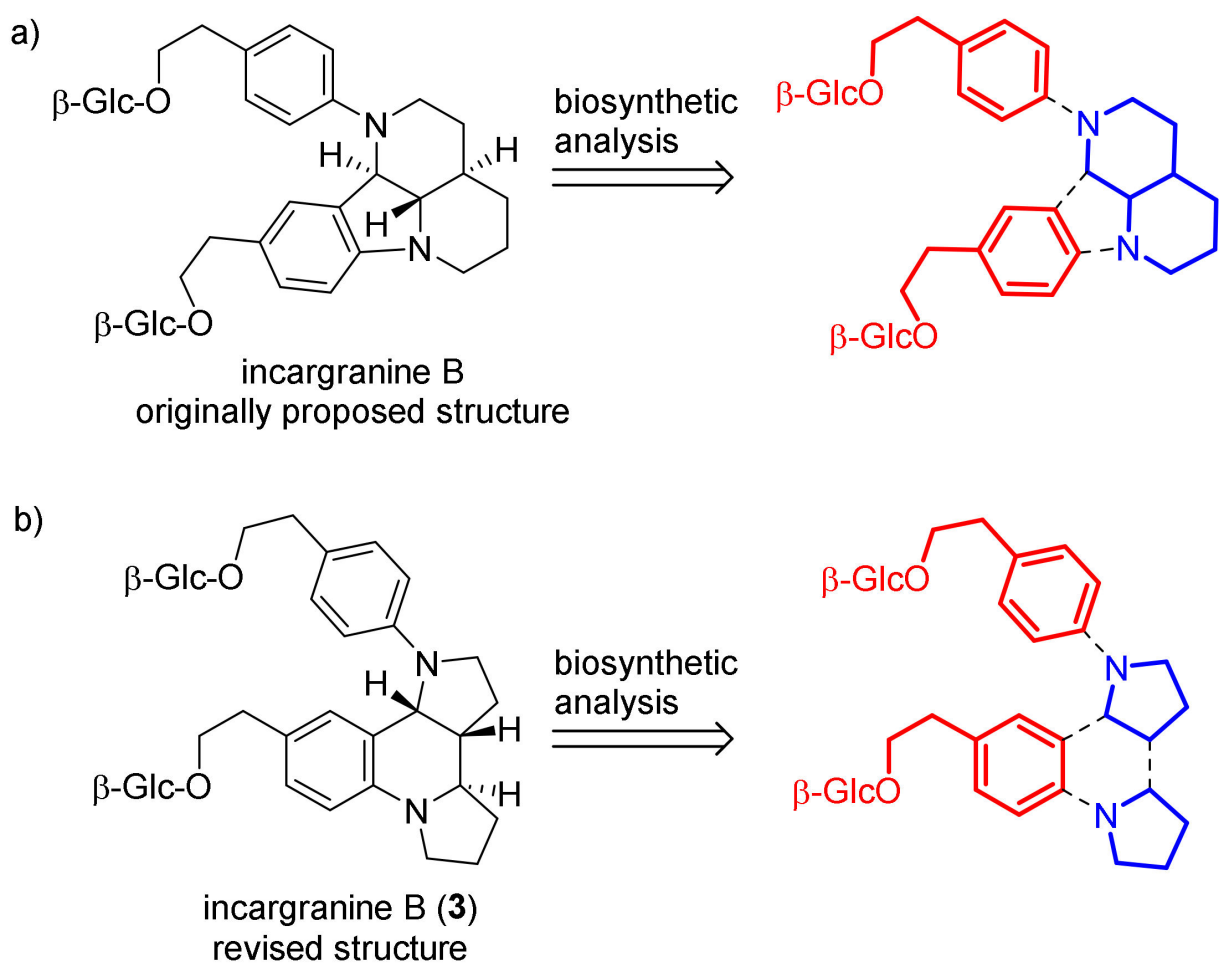
Biosynthetic analysis of incargranine B. (**a**) Originally proposed structure. (**b**) Revised structure.

As mentioned in the introduction, the Zhang group isolated incargranine B (**3**) in 2010 from *Incarvillea mairei* var. *grandiflora* [25]. Based on the spectroscopical data, they proposed an indolo-[1,7]naphthyridine core structure for the alkaloid (Figure 2a). When Lawrence and co-workers later studied the most likely biogenesis route to incargranine B (**3**) from the main sources of alkaloid building blocks, they firstly recognized that incargranine B appeared to be a dimer biosynthesized from the glycosylated tyrosine precursor, salidroside (Figure 2, red part) [26]. However, they struggled to propose a plausible mechanism for the synthesis of the heterocyclic part (Figure 2a, blue part) of incargranine B. This led them to hypothesize that the originally proposed structure was wrongly assigned and that incargranine B in fact was a dipyrroloquinoline (Figure 2b) [26].

**Scheme 37:**
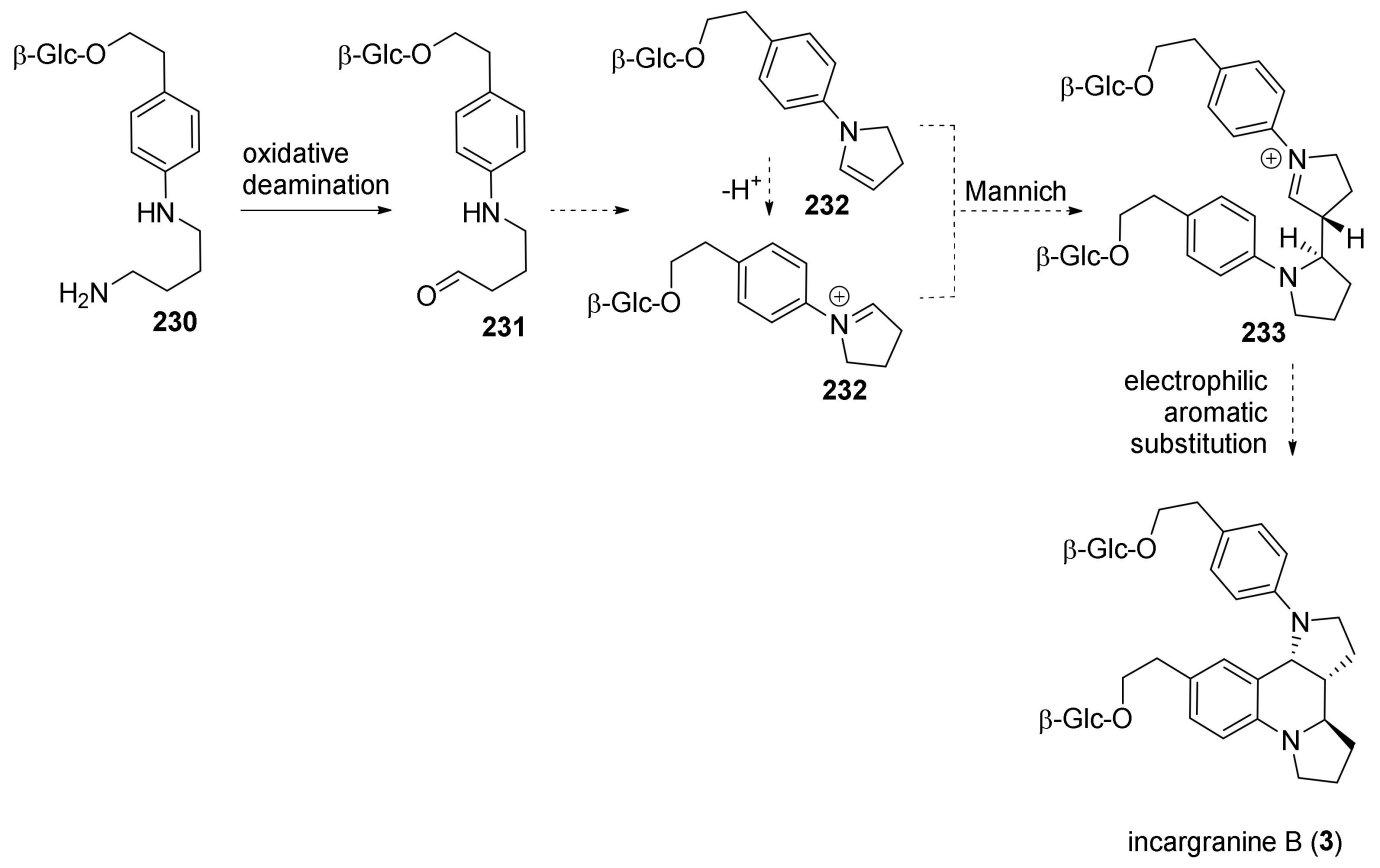
Lawrence and co-workers’ proposed biosynthetic pathway to incargranine B (**3**).

With this hypothesis in mind, Lawrence and co-workers suggested a biosynthetic route towards incargranine B (**3**), where phenylethanoid-diamine **230** was a reasonable precursor (Scheme 37). Diamine **230** could undergo oxidative deamination to form an amino aldehyde **231** that would undergo an intramolecular condensation to give imine/enamine **232**. Dimerization of the imine/enamine **232** through a Mannich reaction to form an imine intermediate **233** that could be trapped in an electrophilic aromatic substitution reaction would provide incargranine B (**3**).

**Scheme 38:**
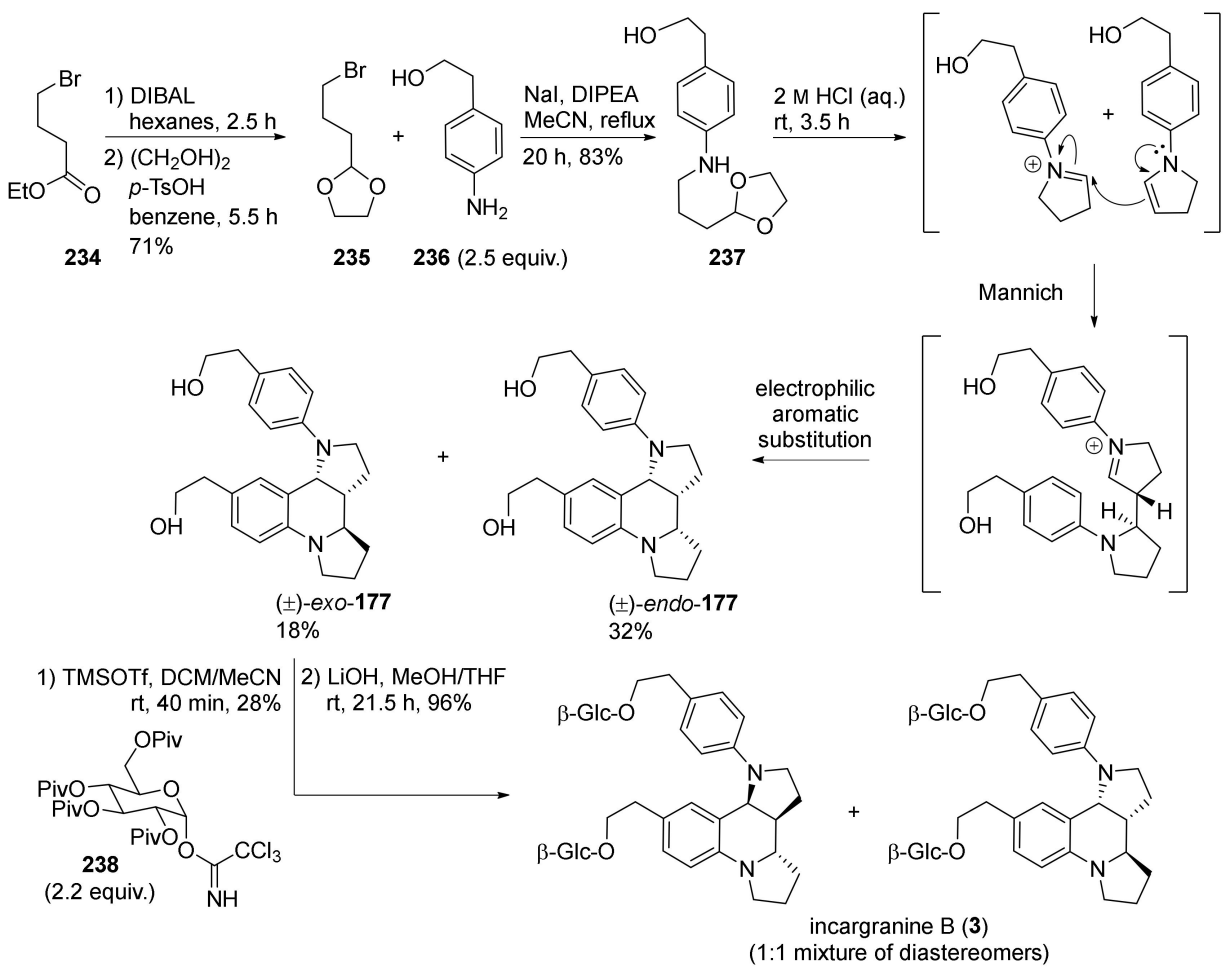
Synthesis of Incargranine B completed in the Lawrence group.

To confirm their structural revision work and suggested biosynthetic pathway, Lawrence and co-workers completed a biomimetic total synthesis of the revised structure for incargranine B (**3**) (Scheme 38). Reduction of ester **234** with DIBAL followed by acetal protection of the resulting aldehyde provided acetal **235** in 71% yield. Alkylation of aniline **236** with acetal **235** gave the dimerization precursor, acetal **237**. Acidic hydrolysis of the acetal to form the corresponding aldehyde facilitated condensation with the amine to form the enamine in equilibration with the iminium ion. Further dimerization in a Mannich reaction followed by electrophilic aromatic substitution provided dipyrroloquinoline **177** as a close to racemic mixture (scalemic mixture). The isolated diastereomer (±)-exo-**177** was further glycosylated with *O*-pivaloyl-protected glucoside **238**. After basic deprotection of pivaloyl-groups with lithium hydroxide, incargranine B (**3**) was obtained as a mixture of diastereomers. The spectroscopic data of the synthesized incargranine B (**3**) were in full accord with the data reported by Zhang for the isolated natural product.

**Figure 3:**
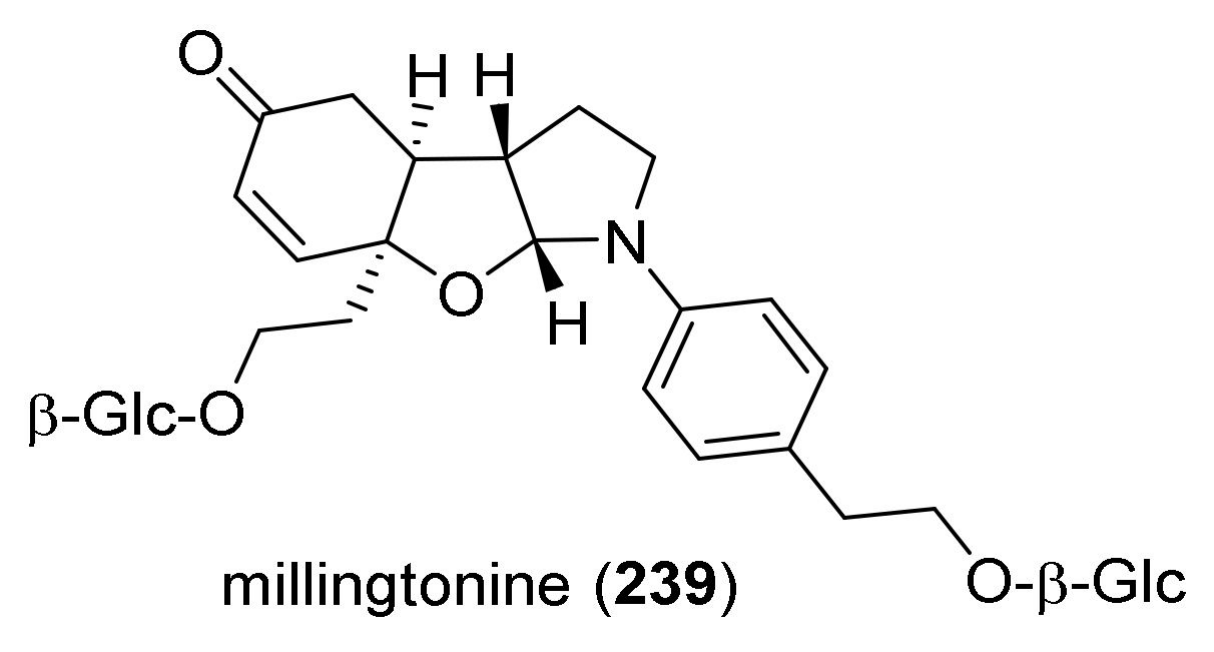
Millingtonine (239).

The optical rotation of the diastereomeric mixture **3** (α = −16.7 (*c* = 0.275, MeOH)) was close to the reported optical rotation of the isolated natural product (α = −12 (*c* = 0.275, MeOH)). Lawrence and co-workers therefore suggested that the isolated natural product **3** also consists of a mixture of diastereomers. They further drew a parallel to the biosynthetically related diglycosidic alkaloid, millingtonine (**239**) (Figure 3), which also naturally occurs as a mixture of diastereomers. The discrepancies of the optical rotation values between the isolated martinellic alkaloids and the synthesized enantiomers have resulted in several groups, suggesting that the martinella alkaloids also most likely are a close to racemic (or scalemic) mixture of enantiomers [30].

**Scheme 39:**
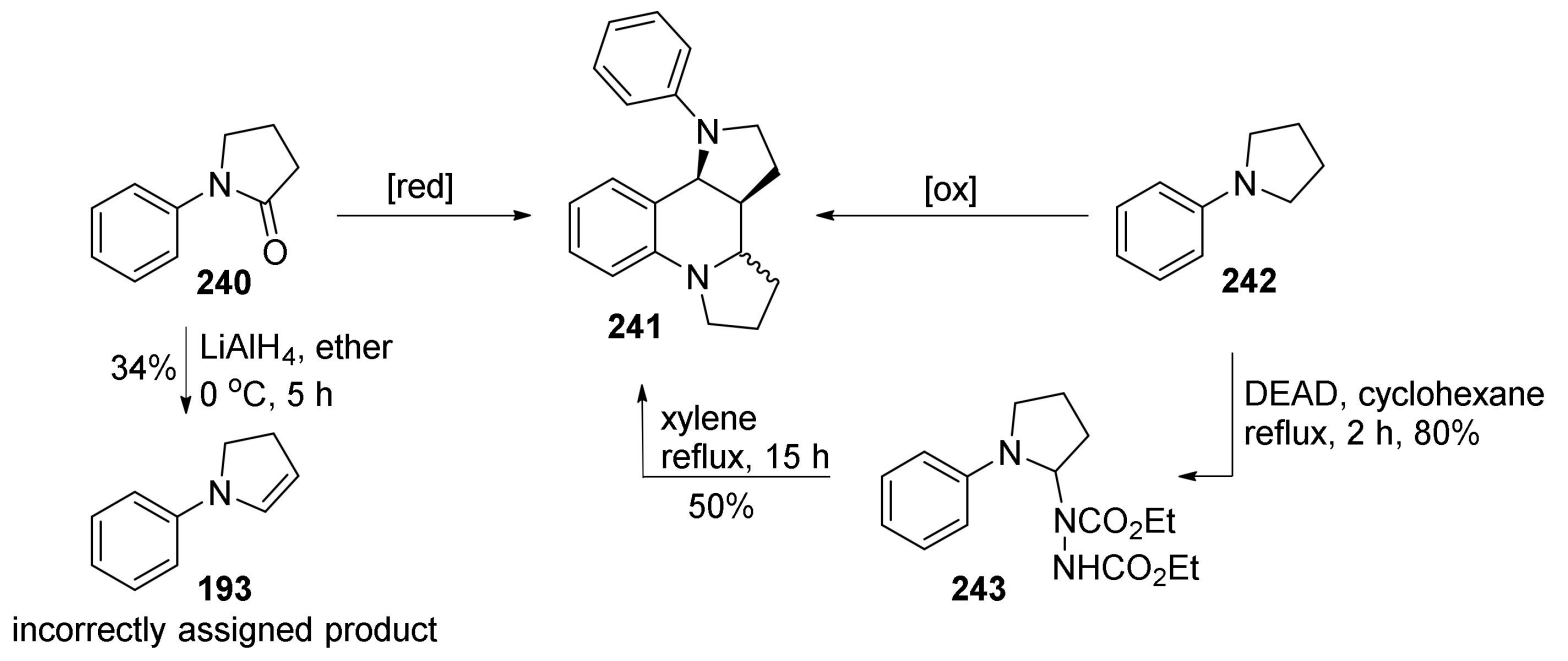
Early work by Wittig and Sommer, followed by structural revision and additional reaction conditions reported by Swan, Wilcock, Kerr, and others to form the dipyrroloquinoline scaffold.

Interestingly, the dipyrroloquinoline scaffold was discovered in the laboratory decades before it was found in nature. The first synthesis of the dipyrroloquinoline was actually conducted in 1955 by Wittig and Sommer, in a lithium aluminium hydride (LiAlH_4_) reduction reaction with lactam **240** (Scheme 39) [99]. The resulting product was incorrectly characterized to be pyrrole **193**, accompanied by a comment on how the properties of this product **193** was remarkably different from its stereoisomer, i.e., the corresponding 2,5-dihydropyrrole. The error was discovered in 1974 by Swan and Wilcock and Kerr and co-workers in two separate studies [100,101]. Both groups described that the product formed under the Wittig and Sommer reaction conditions was in fact the dipyrroloquinoline **241** produced by dimerization of the enamine in equilibrium with the corresponding imine (Table 3, entry 1).

Kerr and co-workers suggested that formation of the endo-product **241** as the major stereoisomer could support a concerted hetero Diels Alder dimerization mechanism [101]. In the same report, they also presented formation of the dimer **241** by oxidation of pyrrolidine **242** with ozone, or by using diethyl azodicarboxylate (DEAD) to form hydrazine **243**, followed by thermal decomposition [101] (Scheme 39, Table 3, entry 2 and 3). Swan and co-workers similarly presented oxidative formation of the dimer 241 from pyrrolidine **242** using gamma-rays or peroxides (Table 3, entry 4–6) [102]. They suggested that the intermediate enamine was, under these conditions, formed from the pyrrolidine radical. When treating pyrrolidine **242** with *t*-butylperoxide, a mixture of stereoisomeric dimers **241** was formed. However, with dibenzoyl peroxide, only the endo-stereoisomer **241** was isolated. This peculiarity was also observed by Rao and Periasamy when they treated pyrrolidine **242** with *t*-butyl hydroperoxide (HYDRO) in the presence of sodium acetate to form the endo-dimer **241** in 72% yield (Table 3, entry 7) [103].

Another approach towards dipyrroloquinoline **241** was presented by Minakata and co-workers in 1997 [104]. They described that oxidation of pyrrolidine **242** with catalytic copper(II) and triethyl amine as an additive under an oxygen atmosphere resulted in the formation of the dimer **241** in 26% yield (Table 3, entry 8). Interestingly, this method favored formation of the exo-stereoisomer **241** in a 15:11 ratio. Buswell and Fleming later showed that the dimer **241** could be made from lactam 240, by treatment with dimethyl phenyl silyl lithium (PhMe_2_SiLi) at −78 °C (Table 3, entry 9) [105]. Due to the high nucleophilicity of PhMe_2_SiLi, Fleming suggested that the dimerization mechanism did not involve formation of an imine intermediate. Instead, he proposed that dimerization occurred between a pyrrolocarbene, formed from α-elimination of dimethyl phenyl silane oxide, and the corresponding dimethyl phenyl silane enolate. Formation of the dipyrroloquinoline **241** as a side product dimer when utilizing *N*-phenyl pyrrolidine in reactions with oxidative conditions is today a well-known issue [106,107,108,109,110,111,112].

**Scheme 40:**
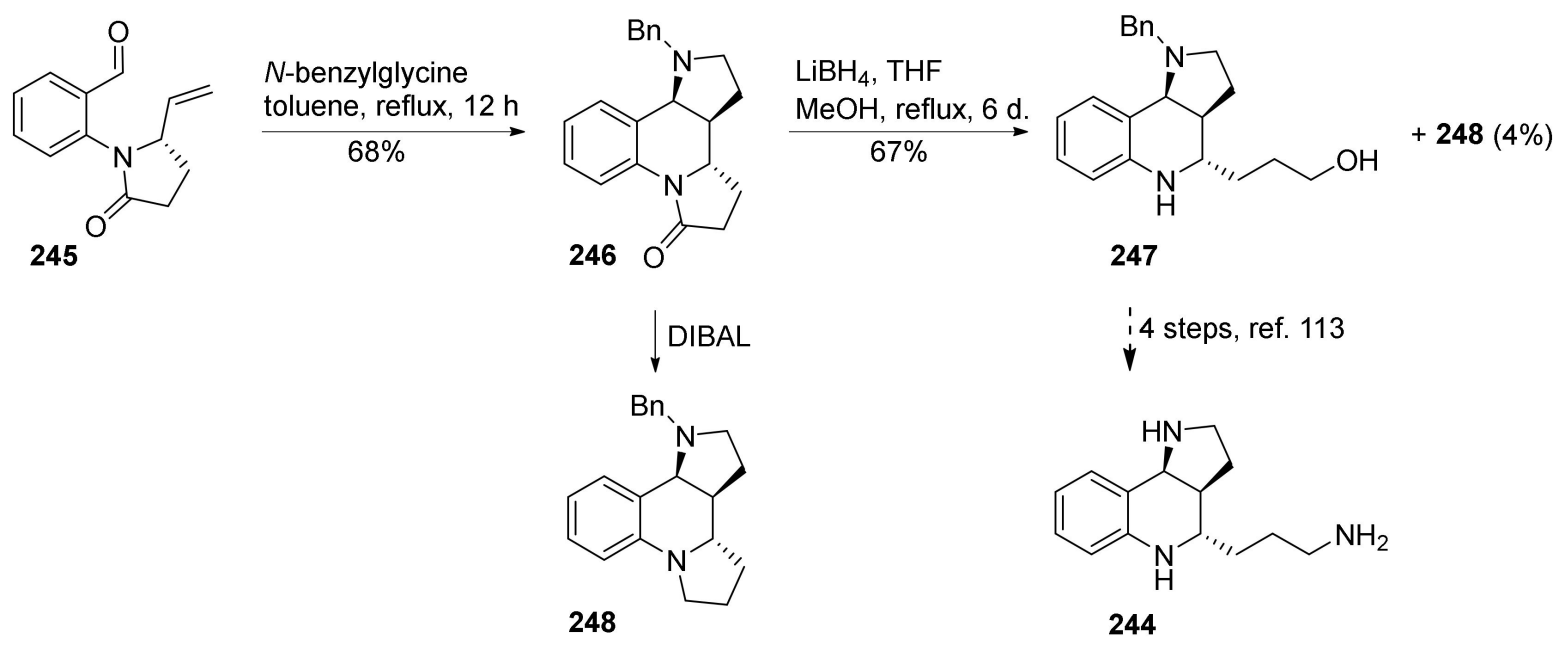
Snider and co-workers’ synthesis of dipyrroloquinoline scaffold.

In their synthesis of triamine **244**, Snider and co-workers prepared dipyrroloquinoline **245** as an intermediate (Scheme 40) [113]. A similar tetracycle **52** was also prepared by the Naito group in the RACE reaction previously mentioned (Scheme 7) [31]. Reduction of amide **246** with lithium borohydride afforded the desired alcohol **247** and minor amounts of pyrroline **248**. Moreover, Snider mentioned that reduction with lithium aluminium hydride gave tricycle **247** and tetracycle **248** in a 1:1 mixture, and that dipyrroloquinoline **248** was formed exclusively upon treatment with DIBAL.

**Scheme 41:**
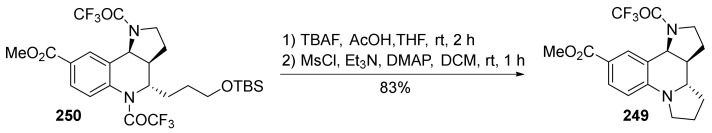
Ma and coworkers’ conversion of pyrroloquinoline **250** to dipyrroloquinoline **249**.

In a similar fashion to Snider, Ma and co-workers synthesized the dipyrroloquinoline **249** as a side product in their asymmetric total synthesis of martinellic acid [29]. *N*-,*O*-deprotection of pyrroloquinoline **250** with TBAF followed by treatment of the corresponding alcohol with methanesulfonyl chloride provided dipyrroloquinoline **249** in a substitution reaction in 83% yield (Scheme 41). The X-ray structure of tetracycle **249** was used to confirm the exo-stereochemistry of the pyrroloquinoline scaffold **250** and consequently also the final product of the synthesis, namely (−)-martinellic acid ((−)-**2**).

**Scheme 42:**
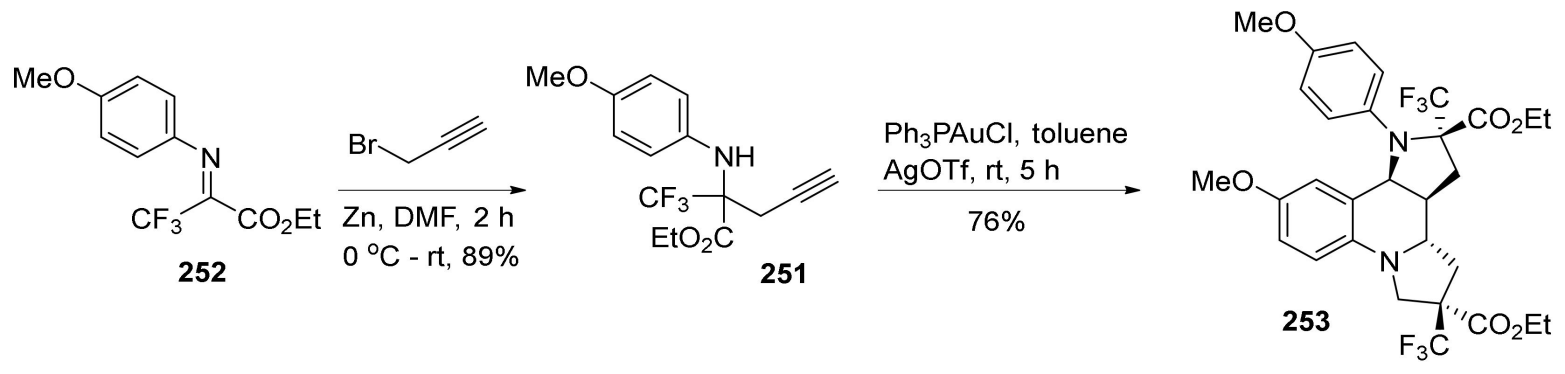
Scaffold synthesis by Fustero and co-workers.

Fustero and co-workers developed a gold catalyzed stereoselective hydroamination of a propargylic amino ester, namely compound **251**, to form the dipyrroloquinoline scaffold as the exo-diastereoisomer [114]. The reaction sequence commenced by treating iminoester **252** with propargylbromide in the presence of zinc providing propargylic amino ester **251** in good yield (Scheme 42). Hydroamination of the propargylic amino ester **251** to form the exo-dimer **253** was catalyzed by triphenylphosphine goldchloride (Ph_3_PAuCl) and silver triflate (AgOTf) as an additive. During the optimization studies the group observed that solvents that were more polar than toluene, such as methanol and acetonitrile, resulted in the formation of more complex product mixtures and consequently lower yields of the desired product. As opposed to the choice of solvent, which had a large effect on the outcome of the reaction, the use of different gold catalysts in the presence of AgOTf, and other additives such as AgNTf_2_, AgSbF_6_, and AgBF_4_ in the presence of Ph_3_PAuCl gave comparable results and yields (68–78%) to the ones obtained under the original conditions.

**Scheme 43:**
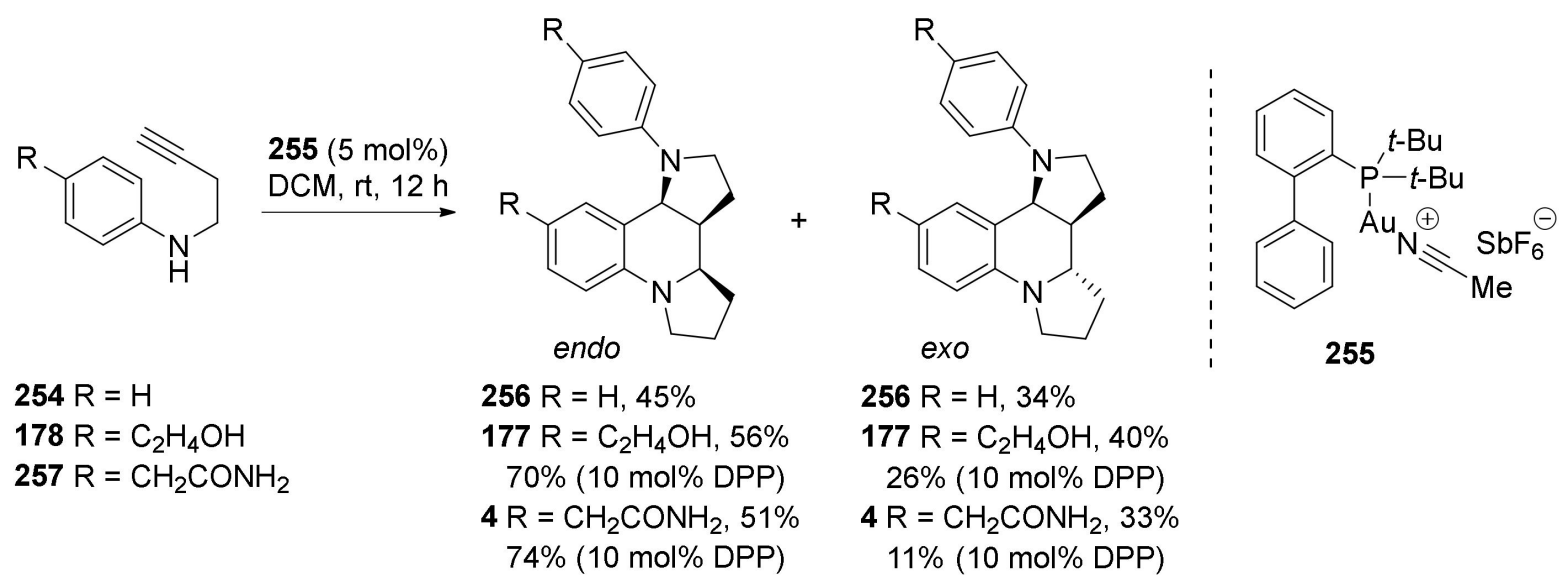
Scaffold synthesis developed in the Liu group.

Another gold-catalyzed hydroamination of an aminoalkyne was described by the Liu group [115]. They presented the synthesis of the dipyrroloquinoline core from a 1,4-aminoalkyne **254** in a gold catalyzed tandem reaction with catalyst **255** (Scheme 43). The reaction sequence started with an intramolecular hydroamination reaction, followed by an aza–Diels Alder reaction, resulting in a 45:34 endo:exo mixture of diastereomer **256**. The reaction scope was further expanded to obtain the Incargranine B aglycon **177** (56:40, endo:exo), along with the natural product seneciobipyrrolidine (**4**, 51:33, endo:exo) from aminoalkynes **178** and **257**, respectively. Endo-diastereoselectivity was enhanced by addition of 10 mol% diphenylphosphate (DPP) to the reaction mixtures.

**Scheme 44:**
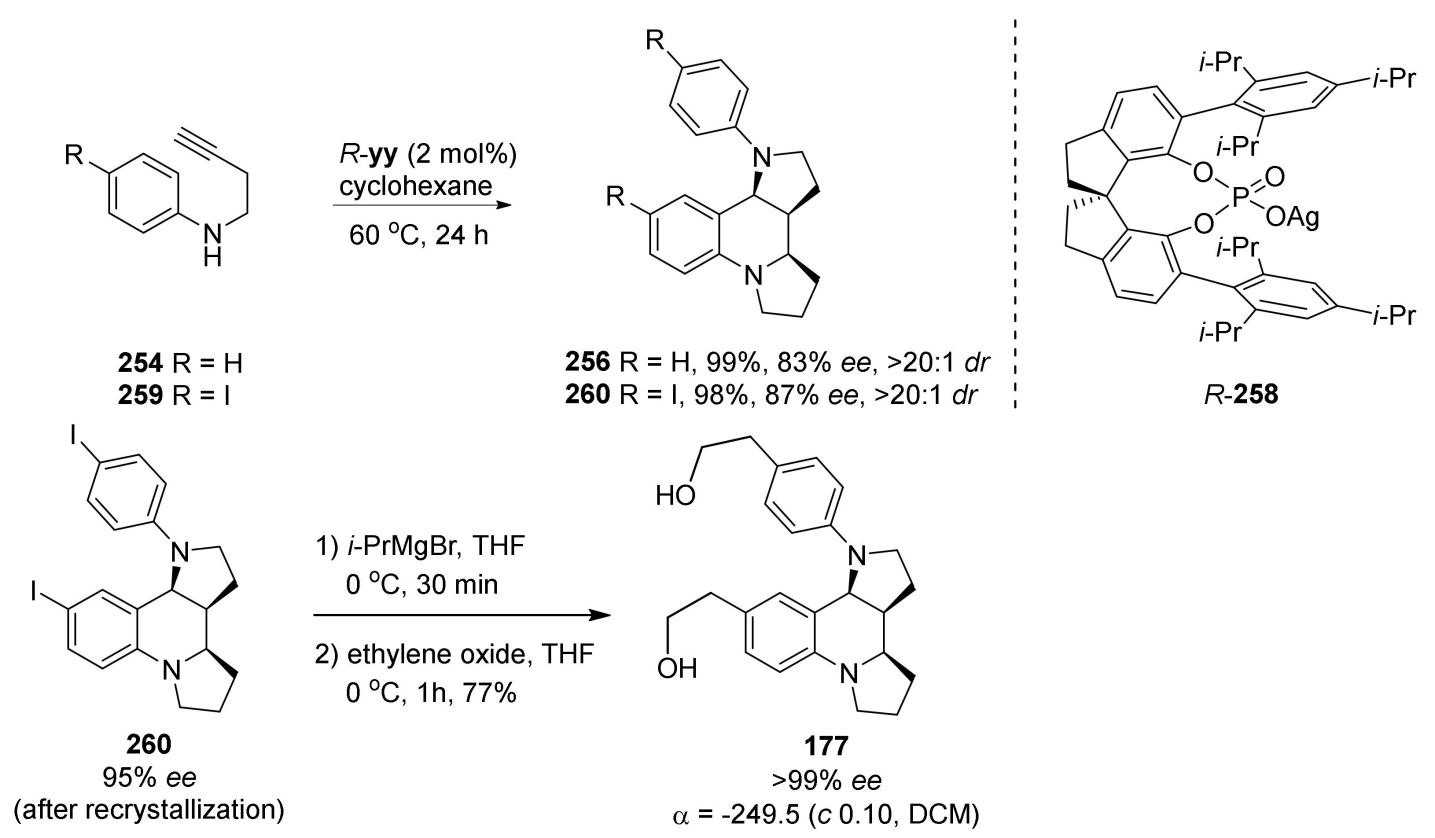
The Liu group’s synthesis of the aglycon moiety of incargranine B.

In their pursuit to enhance the stereochemical selectivity, the Liu group turned to silver catalysis [116]. When using a chiral silver-phosphate catalyst (*R*)-**258** instead of a gold catalyst, the reaction proceeded with a higher enantio- and diastereoselectivities and higher yields on a wide array of aniline–alkyne substrates, exemplified here by alkynes **254** and **259** (Scheme 44). The resulting iodo-endo-product **260** was further converted to the epi-incargranine B aglycon **177** by means of a Grignard reaction with ethylene oxide.

**Scheme 45:**
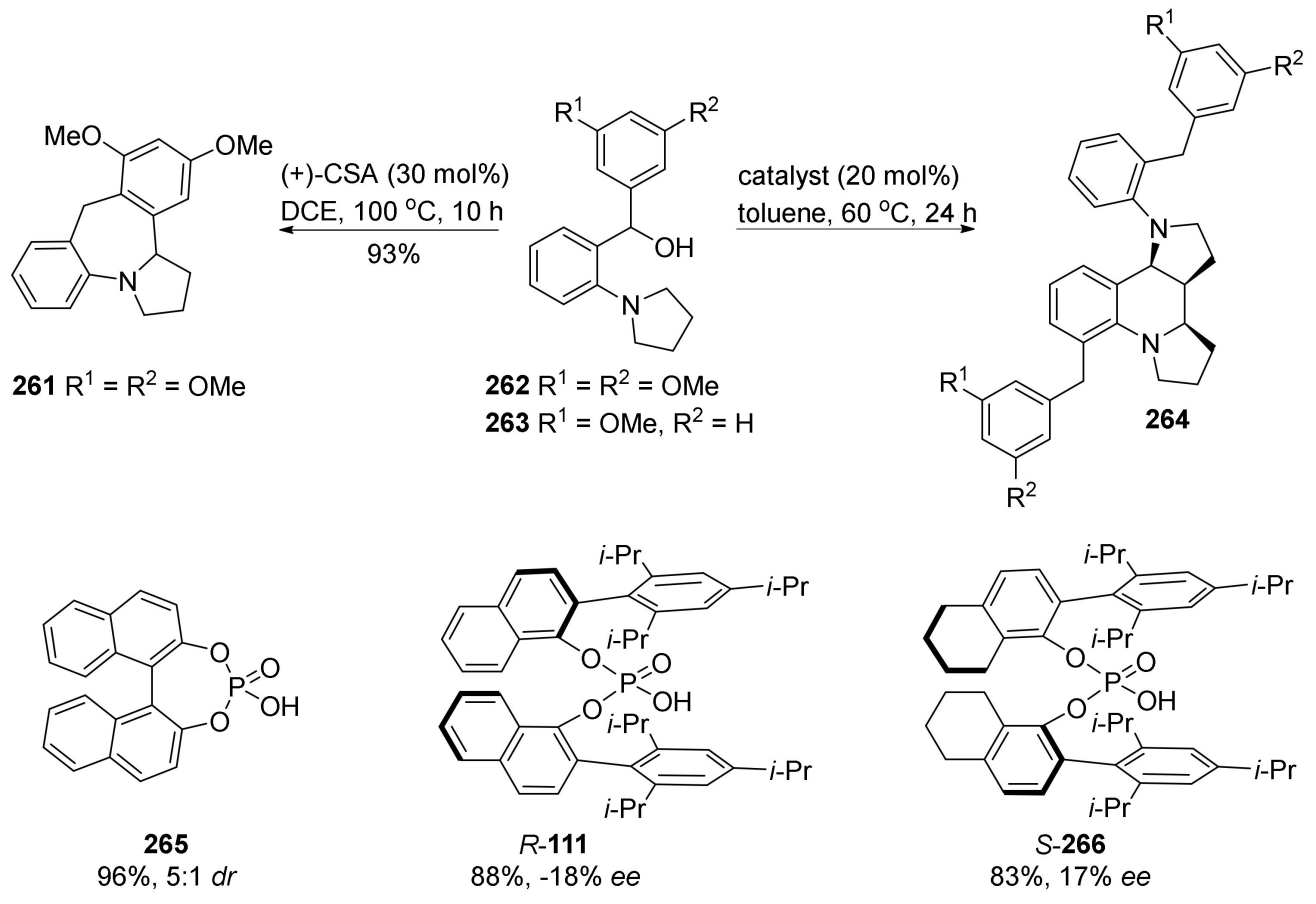
Scaffold synthesis by Xiao and co-workers.

Xiao and co-workers were studying [1,5]-hydride transfer-cyclization reactions for the formation of benazepines **261** from pyrrolidine **262** (Scheme 45) [117]**.** When investigating the scope of this reaction, they discovered that by reducing the number of methoxy substituents on the aromatic ring (the R-groups) from two to one (such as in compound **263**), benzazepines were no longer formed. Instead they obtained dipyrroloquinoline **264** in 20% yield. A study focusing on optimizing reaction conditions showed that by using the acid catalyst phenylphosphate **265** instead of (+)-10-camphorsulfonicacid ((+)-CSA) and conducting the reaction in toluene at 60 °C rather than in DCE at 100 °C gave dipyrroloquinoline **264** in 96% yield and 5:1 diastereomeric ratio, favoring formation of the endo-product (Scheme 45). The group further investigated the catalyst scope for the asymmetric synthesis of dimer **264**. From their collection of acid catalysts, the most efficient were phosphates (*R*)-**111** and (*S*)-**266**; however, these catalysts gave the exo- and endo-dimer **264** in only 18% and 17% ee, respectively.

**Scheme 46:**
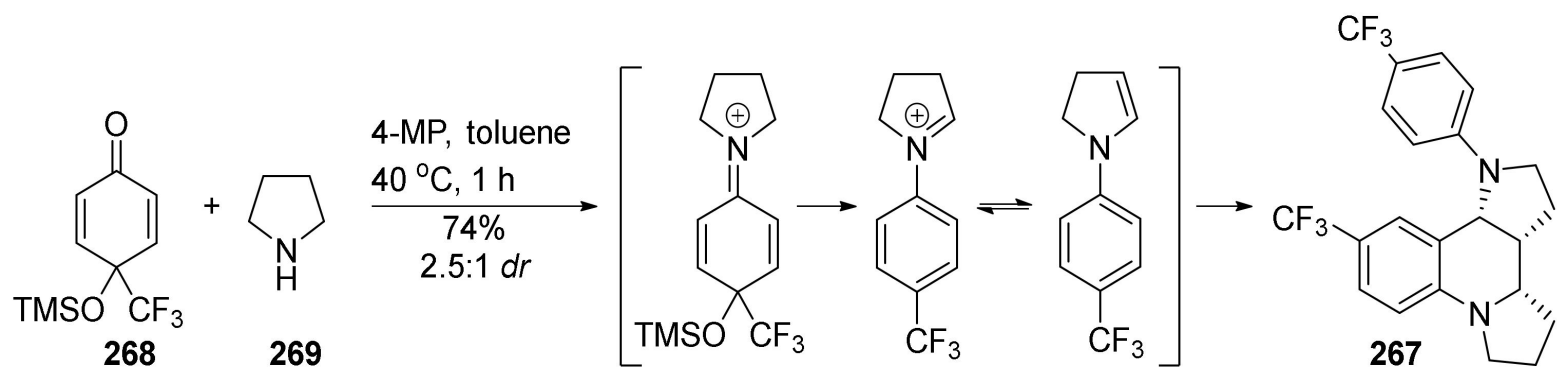
Scaffold synthesis by Liu and co-workers.

Liu and co-workers obtained the dipyrroloquinoline scaffold **267** in their study of in*-*situ formation of iminium ions generated from the condensation of 4-trifluoromethyl-*p*-quinols **268** with cyclic amines, such as pyrrolidine **269** (Scheme 46) [118]. Equal amounts of quinol **268** and amine **269** were stirred in toluene at elevated temperature, in the presence of 4-methoxyphenol (4-MP) as a proton donor, to afford the dipyrroloquinoline **267** in 74% yield and 2.5:1 *dr*.

## 5. Elaborating Biological Properties

**Scheme 47:**
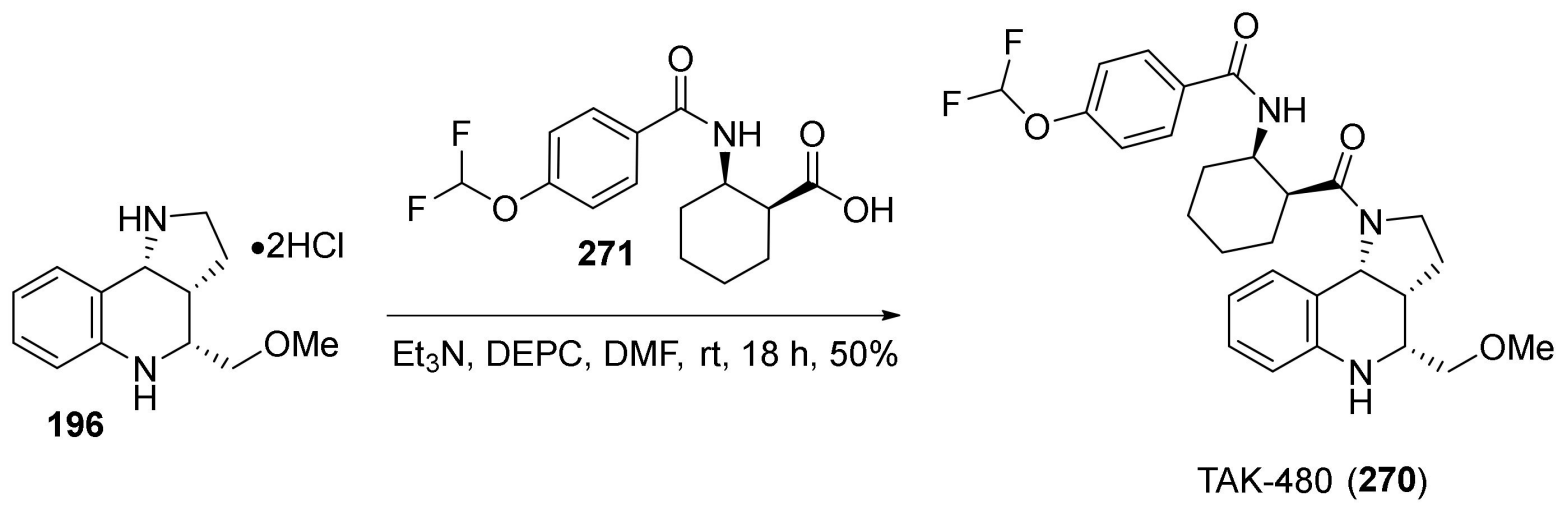
Coupling of pyrroloquinoline **196** and cyclohexane carboxylic acid **271** to form TAK-480 (**270**), a tachykinin NK_2_ receptor antagonist, produced in Takeda Pharmaceutical company.

New and unusual scaffolds are as previously mentioned of great medicinal interest. Synthetic efforts to assemble the pyrroloquinoline scaffold have resulted in discovery of interesting biological activities. In 2005, Takeda Pharmaceutical company patented compounds with properties as NK_2_ receptor antagonist for potential effectiveness in irritable bowel syndrome (IBS) patients [119,120,121]. Compounds in this patent, such as TAK-480 (**270**) (Scheme 47), were constructed around the pyrroloquinoline scaffold, which was assembled using the method developed in Takeda pharmaceutical company (Scheme 33) [92,93]. Drug candidate TAK-480 (**270**) is formed by linking the pyrroloquinoline **196** to the chiral cyclohexane carboxylic acid derivative **271**, using triethyl amine and diethyl cyanophosphonate (DEPC) in DMF (Scheme 47) [122].

**Figure 4:**
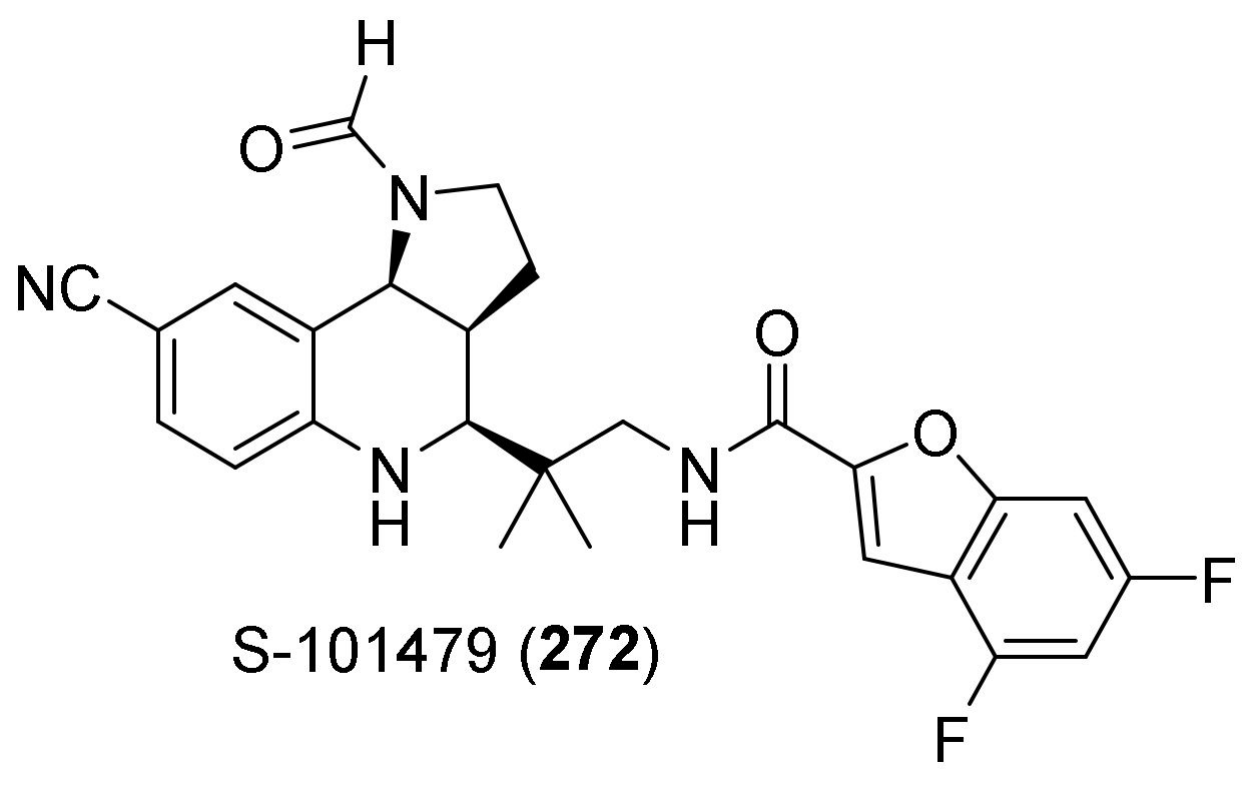
S-101479 (**272**), a drug candidate for treatment of postmenopausal osteoporosis from Kaken Pharmaceuticals.

Another pharmaceutical compound built around the pyrroloquinoline scaffold is S-101479 (**272**), which has been produced by Kaken Pharmaceuticals (Figure 4). According to studies conducted by Furuya and co-workers, compound **272** exhibited a bone-anabolic effect in ovariectomized rats as a non-steroidal selective androgen receptor modulator (SARM) in osteoblastic cells [123,124]. The authors further suggest that, based on their results, the compound **272** could be a drug candidate for treatment of postmenopausal osteoporosis.

**Figure 5:**
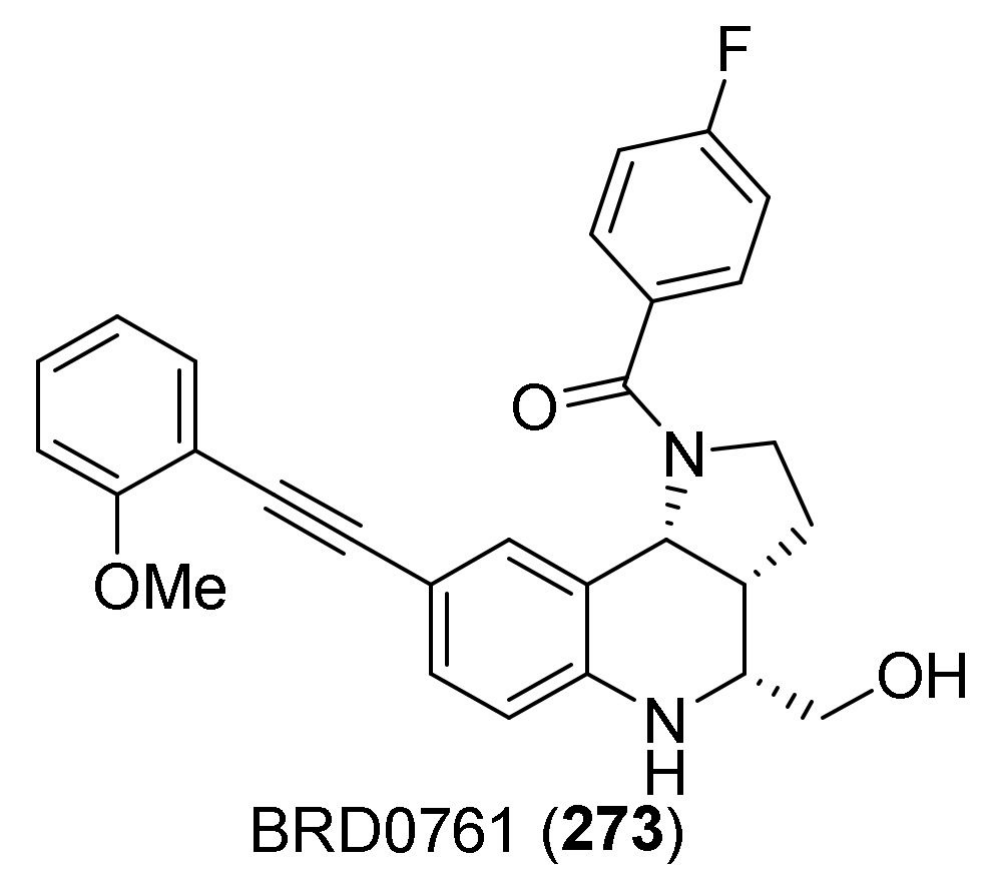
Inhibitor of C. *difficile* specific glutamate racemase.

The Broad institute diversity oriented synthesis (DOS) library, including the pyrroloquinoline-based DOS library developed by Marcaurelle and co-workers, which was discussed previously, has in recent years been used for several high-throughput screenings of small molecules for specific biological activities [65]. Scherer and co-workers conducted a screening of the Broad Institute’s small molecule collection (~100,000 compounds) against the Gram positive, anaerobic, spore-forming, opportunistic pathogen *Clostridium difficile*, a major cause of C. *difficile*-associated diarrhea (CDAD) [125]. With a high-throughput screening of the compound collection against C. *difficile*, a compound containing the pyrroloquinoline scaffold (BRD0761 (**273**)) displayed MIC (µg/mL) = 0.06–1 for a number of clinical C. *difficile* isolates (Figure 5). BRD0761 (**273**) showed greater potency, and more significantly, superior selectivity against C. *difficile* compared to the last resort antibiotic vancomycin, commonly used to treat patients with CDAD. Solubility and permeability (in Caco-2 cells) of compound BRD0761 suggested that oral dosing would be a suitable administration method. Thus, an efficacy study performed on a mouse model of CDAD was conducted with daily oral dosing of 25 mg/kg. BRD0761 (**273**) did reduce mortality rate as well as shedding of C. *difficile* CFU in feces, however not as efficiently as vancomycin.

Investigation of the mode of action for BRD0761 (**273**) via genomic analysis of resistant mutants and in silico studies revealed that the compound most likely targets cell wall biosynthesis via inhibition of glutamate racemase [125]. The enzyme is a known target in other Gram positive bacteria, such as H. *pylori* and M. *tuberculosis* [126,127]. This was, however, a novel target in C. *difficile*. Based on studies from Astra Zeneca that show species specific structure and activity of the glutamate racemase [128], Scherer and co-workers suggested that finding selective C. *difficile* glutamate reductase inhibitors may lead to identification of C. *difficile*-specific antibiotics.

**Figure 6:**
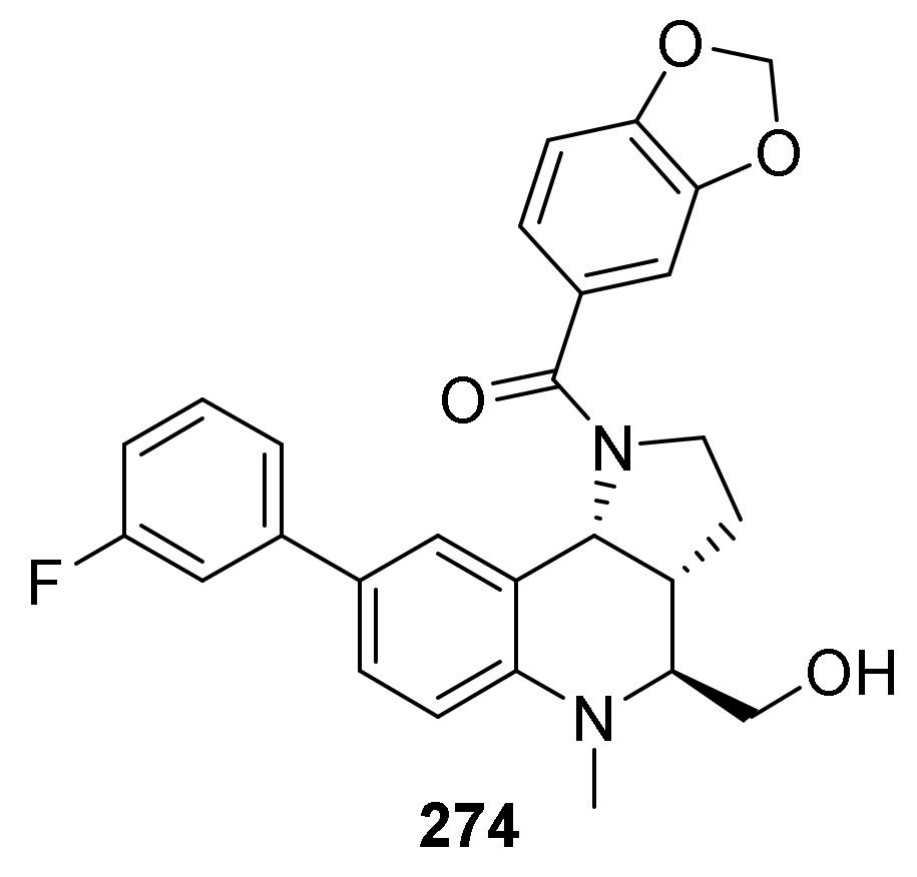
Inhibitor of the aminostransferase BioA.

The Broad institute DOS library was also included in a screening of 256,486 compounds for inhibitors of the aminotransferase BioA, an enzyme in the biotin biosynthetic pathway in *Mycobacterium tuberculosis* [129]. In an initial enzyme inhibition screening of the compound library, the pyrroloquinoline scaffold represented nearly 12% of all hits in the study, including pyrroloquinoline **274** as the screening’s most potent hit (IC_50_ = 75 nM) (Figure 6). However, the activity of compound **274** dropped (MIC_90_ > 100) upon turning to whole cell screening.

**Figure 7:**
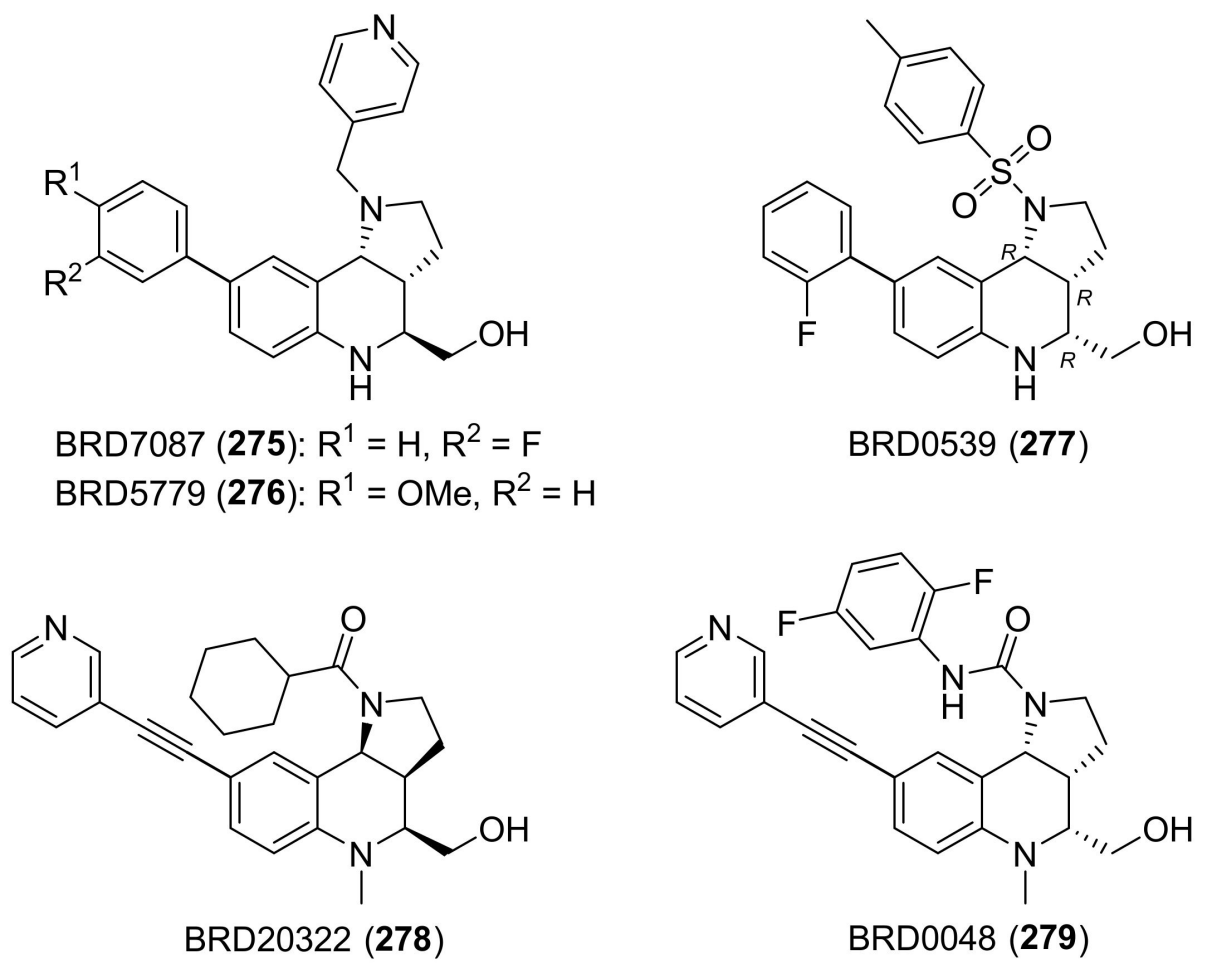
Inhibitors of DNA endonuclease Cas9 in Streptococcus pyogenes (**275**–**277**) and inhibitors of the transcription activation complex, comprised of dCas9:gRNA and transcription-activating SAM domains (**278**–**279**).

As part of genome engineering technologies, control of the CRISPR-Cas9 activity, such as limiting off-target activity of the endonuclease Cas9, is essential. On this note, Choudhary and co-workers conducted a small molecule screen for stable, cell permeable, reversible inhibitors that disrupt the binding of the *Streptococcus pyogenes* endonuclease Cas9 (SpCas9) to DNA [130,131,132]. After an initial screening of representative compounds from the Broad institute DOS library, the “Povarov library”, which included a number of compounds with the pyrroloquinoline scaffold, was selected for further examination. BRD7087 (**275**) and BRD5779 (**276**) (Figure 7) were used to assess the inhibitory activity of the pyrroloquinoline scaffold. Both compounds displayed dose-dependent inhibition of SpCas9, were soluble in phosphate-buffered saline (PBS), and were non-cytotoxic.

After further SpCas9 inhibitory testing of BRD7087 (**275**) analogues, one of the most promising hits, namely BRD0539 (**277**) (Figure 7), was analyzed in a structure–activity relationship (SAR) study of Cas9 inhibition potency. The study included examination of different *N*-linkages, replacement of the 2-fluorophenyl group, and evaluation of the scaffold stereochemistry. Sulfonamides on the pyrrolidine moiety displayed generally higher potency than corresponding amides. Displacement of the para-methyl group on the benzylic sulfonamide with para-fluoro, para-methoxy, or meta-methyl resulted in reduced inhibitory activity, as did introduction of heteroatoms (N and O) onto the aforementioned aromatic ring. Reduced activity was observed upon replacing the 2-fluorophenyl moiety with an alkenyl or a 3-*N*,*N*-dimethyl–carbamoyl–phenyl group, and also by introducing alkynyl spacers (such as seen in compounds **278** and **279**). Finally, elucidation of the four possible stereoisomers confirmed that the pyrroloquinoline 277 with *R,R,R*-stereochemistry was the most potent inhibitor of SpCas9-DNA binding. In an equivalent approach to landing BRD0539 (**277**) as a functional potent SpCas9 inhibitor, BRD20322 (**278**) and BRD0048 (**279**) were identified as potent inhibitors of the transcription activation complex (Figure 7).

## 6. Concluding Remarks

Nature is an inexhaustible source of biologically interesting molecules that have benefitted humanity from its use as herbal medicine to isolation, characterization, and further synthetic manipulation of active ingredients. The biologically active martinella alkaloids in one such example. The chiral core structure found in the alkaloids, namely the hexahydropyrrolo-[3,2-c]-quinoline scaffold, has been an attractive synthetic target since its discovery in 1995. Exploration of the chemically diverse routes to this scaffold has laid the foundation for further elaboration of its potential medicinal benefits, aided by library syntheses and biological screening. As demonstrated by the Lawrence group, synthetic preparation of natural alkaloids also serves to control and revise the characterized structure of isolated natural products.

Though an excessive assembly of synthetic pathways towards the pyrroloquinoline scaffold has been prepared, much is yet to be discovered. The high biological activity found in the examination of the Broad Institute’s DoS Povarov library together with the limited number of biological assays of compounds assembled around the pyrroloquinoline structure shows that this scaffold is still an underexplored source for biologically active compounds. For further exploration of the chemical utility of this scaffold, systematic approaches, such as library screens, are required. On the other hand, synthetically challenging compounds are usually excluded from such libraries and SAR-studies that delimit the selection of compounds to be synthesized follow certain rules, such as the Lipinski’s rule of five. Therefore, fundamental curiosity-driven research is still needed for serendipitous discovery of compounds and their biological properties.

## Figures and Tables

**Table 1:** Reported optical rotation for the isolated and synthetically prepared natural products martinelline (**1**), martinellic acid (**2**), incargranine B (**3**), and seneciobipyrrolidine (**4**).

Compound	Optical Rotation [α_D_]	Concentration (mg/ 10 cm^3^)	Reference
**1** (isolated)	+9.4	0.02	[19]
(−)-**1**	−108.0	0.09	[30]
(+)-**1**	+98.6	0.02	[30]
**2** (isolated)	−8.5	0.01	[19]
(−)-**2**	−122.7	0.37	[29]
(−)-**2**	−118	0.3	[32]
(−)-**2**	−164.3	0.14	[30]
(+)-**2**	+165.5	0.11	[30]
(−)-**2**	−164.8	0.33	[31]
**3** (isolated)	−12	0.275	[25]
(±)-**3**	−16.7	0.275	[26]
**4** (isolated)	−72.9	0.10	[27]

**Table 2:** Reaction conditions and outcome from the Povarov reaction between the aromatic imine **75** and enamide **73a**–**d** (Scheme 11).

Entry	Reaction Conditions	R^1^	R^2^	Enamide	Yield	Endo:Exo	Reference
1	InCl_3_ (2 equiv.), MeCN, rt, 30 min	H	H	**73b**	41%	1:1	[58]
2		H	2-NO_2_	**73b**	50%	2:1	
3	Zn(OTf)_2_ (10 mol%), DCM, rt	H	2-OH	**73c**	42%	>20:1	[59]
4	**76** (10 mol%), 5 Å MS, *n*-hexane −40 °C, 72 h	H	H	**73a**	94%	>20:1	[60]
5	**77** (10 mol%), MeCN, rt, 0.5–1 h	H	H	**73a**	95%	43:57	[61]
6	**78** (10 mol%), MeCN, rt, 27 h	4-OMe	H	**73d**	86%	53:47	[62]
7	Micellar-SO_3_H, H_2_O, 25 °C, 18 h	H	4-O-DNA	**73d**	>90%	NR	[63]

NR = Not reported.

**Table 3:** Oxidative and reductive reaction conditions for formation of dipyrroloquinoline **241**.

Entry	Starting Material	Conditions	Yield ^a^	Endo:Exo	Reference
1	**240**	LiAlH_4_, ether, rt, 3.5 h	27%	38:36	[100]
2	**242**	O_3_, *n*-hexane, 0 °C	41%	NR	[101]
3	**242**	1) DEAD, cyclohexane, reflux, 2 h, 80% 2) xylene, reflux, 15 h	50%	28:22	[101]
4	**242**	γ-irradiation, 17 d	9%	~1:1	[102]
5	**242**	di-*t*-butylperoxide, 140 °C, 44 h	NR	~1:1	[102]
6	**242**	Dibenzoyl peroxide, MeCN, 0 °C, 9 h	28%	1:0	[102]
7	**242**	*t*-BuOOH, NaOAc•3H_2_O, cyclohexane 70 °C, 24 h	72%	1:0	[103]
8	**242**	Cu(OAc)_2_, O_2_, Et_3_N	26%	11:15	[104]
9	**240**	PhMe_2_SiLi, −78 to −20 °C	47%	31:16	[105]

^a^ isolated combined yield of stereoisomers; NR = not reported.

## Data Availability

Supporting data for our results can be obtained by contacting the corresponding author.
